# Synapse-like specializations at dopamine release sites orchestrate efficient and precise neuromodulatory signaling

**DOI:** 10.1126/sciadv.aei0203

**Published:** 2026-10-02

**Authors:** Chandima Bulumulla, Deng Zhang, Deepika Walpita, Nirmala Iyer, Mark Eddison, David Ackerman, Hideo Otsuna, Xianling Zhao, Shuqin Zhang, Shihong M. Gao, Nan Wang, Abraham G. Beyene

**Affiliations:** Janelia Research Campus, Howard Hughes Medical Institute, Ashburn, VA 20147, USA.

## Abstract

Neuromodulators are generally understood to act through expansive projections that broadcast signals relatively indiscriminately, in stark contrast to the precise, synapse-bound organization of fast neurotransmitters like glutamate and GABA. This dichotomy has left the local architecture of neuromodulatory release and receptor engagement largely obscure. Here, using dopamine as a model system, we reveal that neuromodulatory signaling can be guided by precise molecular specificity. We show that dopamine axons exhibit a tropism for neurons expressing D1 or D2 dopamine receptors. Single-bouton–resolved optical imaging of release shows that dopamine is not broadcast stochastically but is highly localized to varicosities that form direct, synapse-like appositions with receptor-expressing somata and dendrites. To map endogenous receptor organization relative to release sites, we generated ALFA-tagged D1 and D2 receptor knock-in mice (ALFADoR mice). In cultures and intact tissue, D1 and D2 receptors formed discrete puncta rather than exhibiting diffuse membrane distributions. Across striatum, amygdala, and prefrontal cortex, receptor puncta were preferentially positioned near dopamine varicosities, and apposed receptor puncta were larger than nonapposed puncta. These findings reveal a synapse-like architecture for dopamine signaling in which release sites and receptor clusters are spatially coupled to support a precise and efficient neuromodulation.

## INTRODUCTION

Two types of chemical signaling facilitate interneuronal communication in the brain. The first type, fast synaptic transmission, occurs between precisely coupled synaptic partners, where the activation of ligand-gated ion channels elicits depolarizing or hyperpolarizing electrical responses. The molecular and cellular mechanisms underlying fast synaptic transmission have been extensively studied (*1*, *2*). The second type of communication involves neuromodulators, which are thought to be released volumetrically and act diffusively on their targets in a paracrine fashion (*3*). Although volumetrically released neuromodulators control the efficacy of fast synaptic transmission and, therefore, play a critical role in neuronal computation that underlies cognition and behavior (*4*, *5*), the precise nature of their release sites and signaling is less well understood and remains an active area of research (*6*, *7*).

Historically, dopamine has served as the model system for neuromodulatory signaling. The volume transmission framework, supported by early histochemical and ultrastructural studies, proposed that dopamine is released from axonal varicosities lacking synaptic specializations, allowing it to diffuse freely through the extracellular space to activate distant receptors (*8*–*11*). However, this interpretation has been contested. While some electron microscopy studies reported that most dopamine axons lack classical synapses (*12*–*15*), others described synapse-like appositions between dopamine terminals and postsynaptic processes (*16*–*19*). Moreover, physiological and behavioral studies have shown that dopamine neuron firing can influence behavior with millisecond-to-second precision (*20*–*24*). These temporal dynamics are difficult to reconcile with purely diffuse signaling. These discrepancies raise a fundamental question: Does dopamine signaling use synaptic mechanisms to achieve spatial and temporal specificity, and, if so, how are these organized at the cellular level?

A key limitation underlying these discrepancies is the lack of functional data correlating dopamine release dynamics with synaptic ultrastructure and receptor localization. Prior work either mapped dopamine varicosities histologically without functional activity data (*12*–*14*, *25*) or measured extracellular dopamine dynamics at low spatial resolution, obscuring single-varicosity mechanisms (*26*, *27*). Consequently, it remains unclear whether dopamine release is stochastic or targeted and whether synaptic architecture enhances neuromodulatory precision. Here, we combine single-bouton optical recording of dopamine release with correlated immunofluorescence, transcriptomics, and expansion and electron microscopy, together with dopamine receptor profiling in newly generated transgenic knock-in (KI) mice, to address these gaps. We show that dopaminergic (DA) axons preferentially form synapse-like contacts with neurons expressing dopamine receptors and that release competence strictly depends on anatomical apposition. Varicosities lacking such appositions or contacting receptor-negative processes remain quiescent. Functionally active varicosities contain canonical presynaptic specializations (Bassoon, Rim, Munc13-1, and others), are juxtaposed against dopamine receptor clusters visualized in our ALFA-tag knock in (ALFADoR) mice, and evoke localized cyclic adenosine 3′,5′-monophosphate (cAMP) responses in their targets. These findings suggest that dopamine neuromodulation uses a hybrid signaling modality, integrating synaptic architecture for precision and efficiency while maintaining the biochemical complexity of G protein–coupled receptor (GPCR) signaling.

## RESULTS

### Dopamine is released from varicosities that are apposed to neuronal dendrites and somata

Our study uses two complementary nanomaterial-based fluorescent dopamine sensors: DopaFilm and nIRCat. DopaFilm consists of optical dopamine nanosensors drop casted onto two-dimensional (2D) glass substrates (*28*), whereas nIRCat is a solution-phase version used in intact tissue slices (*29*). Both nanosensor modalities exhibit rapid, intensiometric fluorescence modulation in response to dopamine. Previous characterization of DopaFilm established its steady-state concentration response to exogenous dopamine, with median effective concentration of ∼268 nM (*28*), and additional calibration experiments using local dopamine puff application confirmed robust responses across a broad range of dopamine concentrations (fig. S1, A to C). For experiments on DopaFilm, we generated primary DA neurons from postnatal day 1 (P1) to P2 pups of *Slc6a3*-Cre (DAT-Cre) mice crossed with Ai9 reporter mice. DA neurons were cocultured with either cortical (DA:C) or striatal (DA:S) neurons. By growing a sparse monolayer of primary neuron cocultures on DopaFilm, we imaged evoked and spontaneous dopamine release from dopamine neurons with single bouton resolution (Fig. 1, A and B). DA hotspots on DopaFilm had a mean size of 2.43 ± 0.91 μm, latency to peak (rise time) of 0.66 ± 0.42 s, a peak Δ*F*/*F* of 14.8 ± 3.2%, and cleared with a first-order time constant (τ) of 10.57 ± 4.85 s (all means ± SD). DopaFilm readily detected electrically evoked, spontaneous circuit-driven, and tetrodotoxin-resistant spontaneous dopamine release events (fig. S1, D and E), indicating that hotspot detection is not restricted to large-amplitude dopamine transients. We corroborated this hotspot-like organization of dopamine release using nIRCat by imaging release from dopamine axons in the dorsomedial striatum (DMS) and basolateral amygdala (BLA) in acute slice preparations (Fig. 1C). Hotspot analysis in the DMS and BLA revealed localized dopamine release with mean sizes of 2.12 ± 0.72 μm and 2.35 ± 0.81 μm, respectively. Because DopaFilm and nIRCat differ in sensor geometry and are deployed in distinct experimental preparations, these values should be interpreted within each assay rather than as direct quantitative comparisons between culture and slice. We observed differences in peak Δ*F*/*F* (DMS, 5.02 ± 2.02%; and BLA, 7.58 ± 4.11%), clearance kinetics (DMS, 7.87 ± 2.42 s; and BLA, 19.61 ± 12.41 s), and latency to peak (DMS, 1.19 ± 0.51 s; and BLA, 2.69 ± 1.31 s) (all means ± SD) (Fig. 1, D and E). Collectively, these data demonstrate that spatially confined, hotspot-like organization is a key hallmark of extracellular dopamine dynamics and that dopamine nanosensors enable the visualization of these dynamics in both culture and intact tissue, capturing subtle spatial and temporal differences that likely reflect distinct local molecular and ultrastructural environments. Additionally, DA:S cocultures exhibited significantly greater axonal arborization, measured by total axonal length, branching points, and other morphometric parameters compared with DA:C cocultures (fig. S2). This morphological distinction is consistent with the known in vivo characteristics of their putative anatomical representations. In contrast to mesocortical projections, the mesostriatal pathway, primarily comprising substantia nigra pars compacta (SNc) neurons, is characterized by fine axonal fibers that form dense and highly branched arbors (*30*). Together, these data show that DopaFilm recapitulates key characteristics of extracellular neurochemical dynamics and axonal morphology while reporting activity with subcellular resolution.

**Fig. 1.**
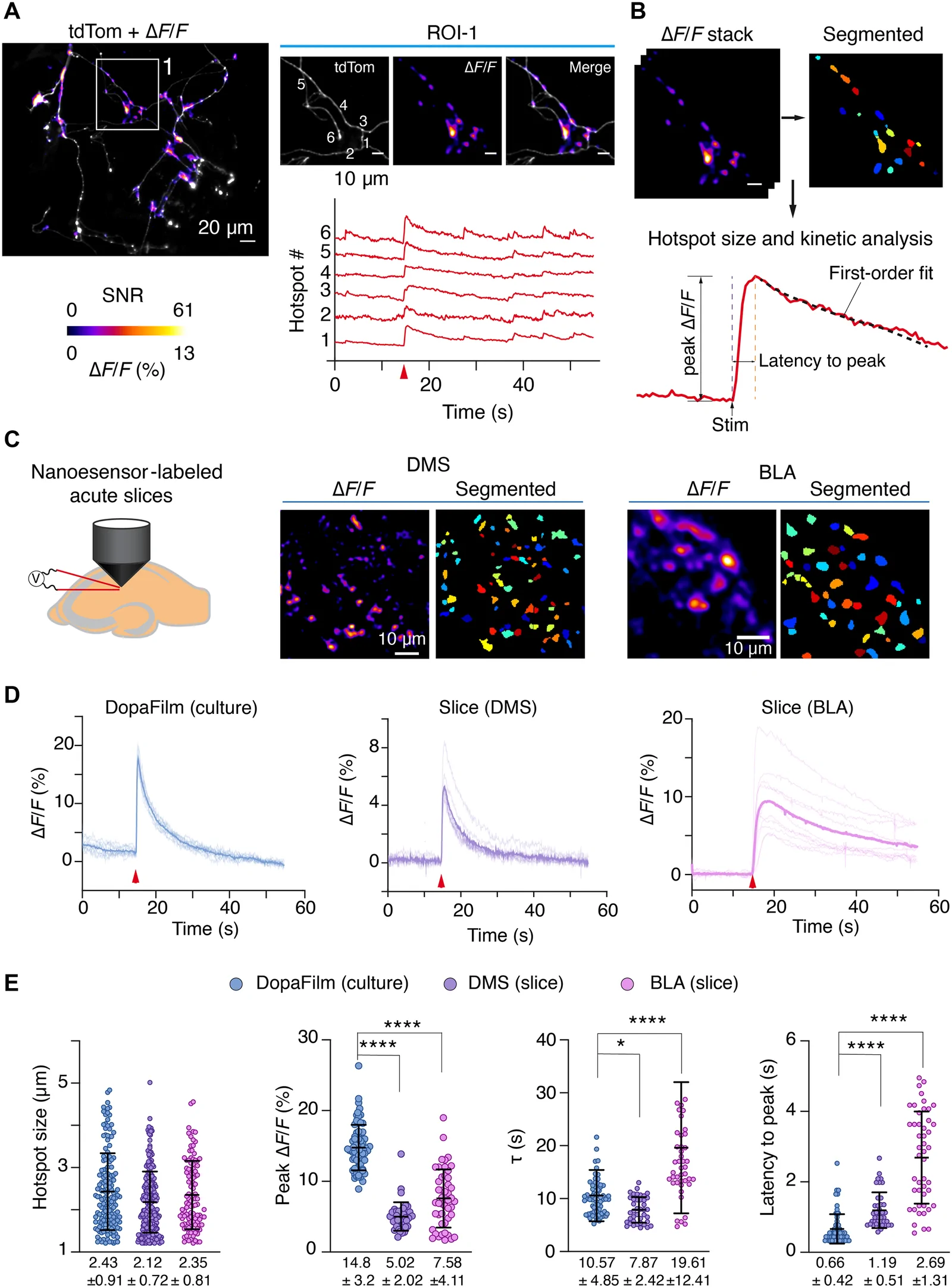
Imaging dopamine release hotspots in primary cultures and acute slices. (**A**) tdTomato (tdTom)–expressing DA axons overlaid with dopamine ∆*F*/*F* hotspots. The ∆*F*/*F* frame is taken ∼300 ms after application of electrical field stimulation (5p/25 Hz, 1-ms pulse width). Right: Close-up of A′ depicted in (A). Scale bars, 10 μm. Bottom: Activity traces corresponding to varicosities 1 to 6 showing a mix of electrically evoked (red arrowhead) and nonevoked (i.e., spontaneous) release events. SNR, signal-to-noise ratio. (**B**) Analyzing extracellular DA dynamics: Hotspots are segmented from ∆*F*/*F* stack, and temporal trace corresponding to each hotspot is extracted for further analysis; peak ∆*F*/*F*, τ from a first-order decay and rise time calculated as “latency to peak” are evaluated. (**C**) Solution phase dopamine nanosensors (nIRCat) enable visualization of dopamine release hotspots in the dorsomedial striatum (DMS) and basolateral amygdala (BLA), which receive axonal projections from the substantia nigra pars compacta (SNc) and ventral tegmental area (VTA), respectively. Activity is electrically evoked (single pulse with bipolar electrodes) in 300-μm-thick slices. (**D**) Representative temporal traces from DopaFilm, DMS, and BLA. Each panel shows mean trace (bold) and individual traces from 10 randomly selected hotspots in the imaging field of view (FOV). Data from one imaging field are shown as an example. (**E**) Comparison of hotspot sizes, peak ∆*F*/*F* (evoked), clearance kinetics (τ), and latency to peak. Each data point represents hotspots pooled from *n* = 10 biological replicates. Means ± SD values are shown at the bottom of each chart. Test of statistical significance: nonparametric Kolmogorov-Smirnov (KS) test. *P < 0.05; ****P < 10^−4^.

Following live-cell imaging on DopaFilm, we conducted post hoc multimodal analyses of dopamine axonal varicosities and their putative targets using immunofluorescence microscopy, spatial transcriptomics, and transmission and volume electron microscopy (Fig. 2). With this approach, we were able to correlate functional activity data (i.e., DA hotspots) with ultrastructural and molecular features with single-bouton precision. Our analyses of DopaFilm data revealed three principal findings regarding dopamine release organization in axonal arbors.

**Fig. 2.**
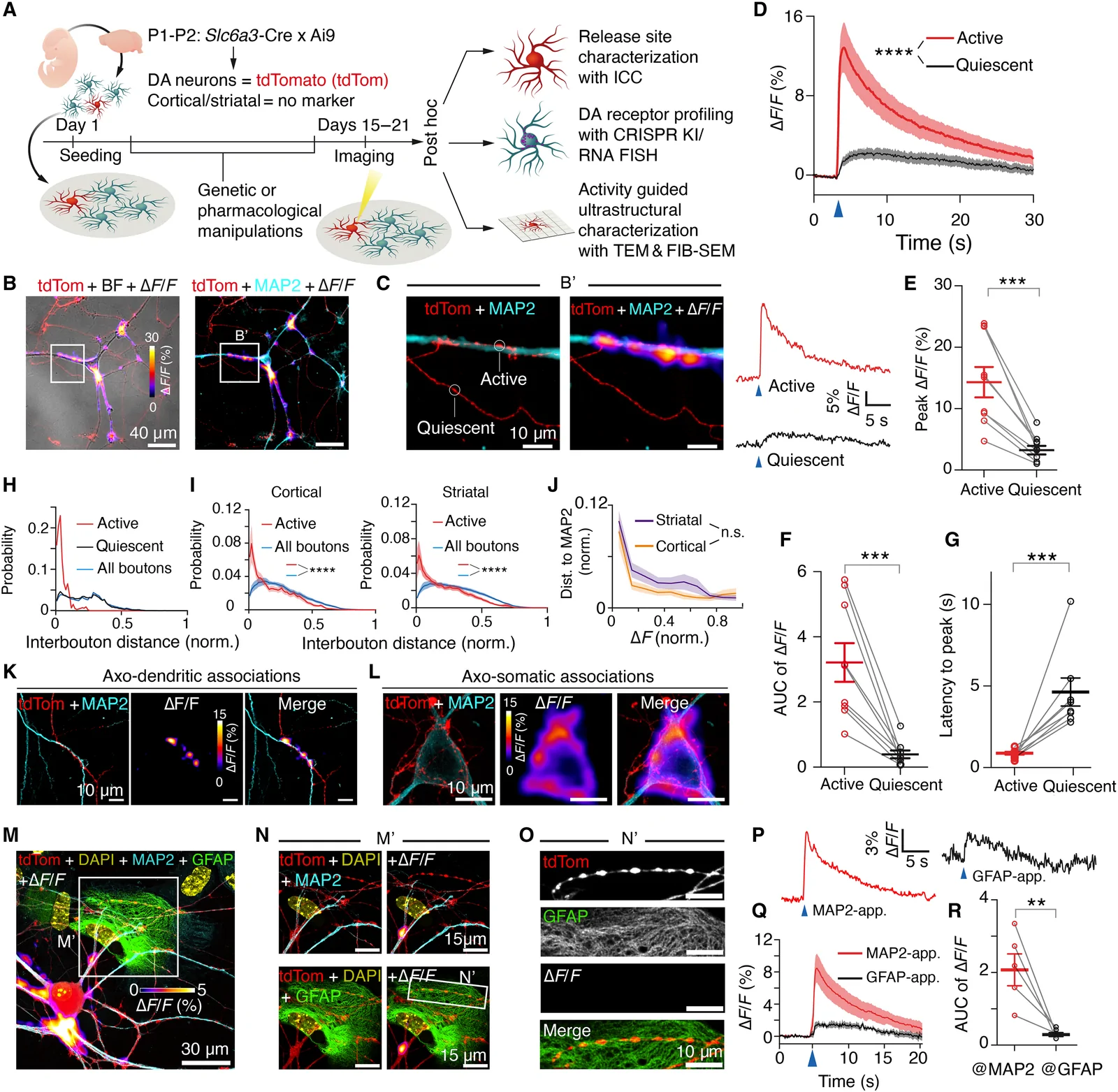
Experimental schematics and DopaFilm activity imaging in dopamine neuron axonal arbors. (**A**) Dopamine and striatal/cortical neurons from P1 to P2 pups are cocultured on DopaFilm for imaging and post hoc analysis. (**B**) A tdTom^+^ dopamine axonal arbor innervating a cortical neuron [bright field (BF)]. Evoked activity (heatmap) is overlaid with BF/tdTom. Right: Post hoc immunocytochemistry against MAP2 (cyan), same FOV. (**C**) Magnified region of interest (ROI) from (B). Active varicosities cluster near MAP2^+^ dendrites; nonapposed ones are quiescent. Right: Representative traces (arrowheads, stimulation time). (**D**) Average traces, active versus quiescent varicosities, *n* = 9 biological replicates (10 each). KS test; ****P < 10^−4^. (**E** to **G**) Peak Δ*F*/*F*, AUC (3-s poststim), and latency to peak, active versus quiescent pooled from *n* = 9 biological replicates; means ± SEM. KS test; ***P < 10^−3^. (**H**) Pairwise-distance distribution, active (red), quiescent (black), and all (blue) boutons, FOV from (B). (**I**) Pooled interbouton-distance distributions, active versus all boutons, DA:C (*n* = 18) and DA:S (*n* = 25) cocultures, normalized. Means ± SEM. KS test; ****P < 10^−4^. (**J**) Normalized Δ*F* versus distance from MAP2^+^ processes; higher Δ*F* occurs closer to MAP2^+^ in both coculture types. Means ± SEM. KS test; n.s., not significant. (**K**) Dopamine axons innervating a cortical dendrite (MAP2); only dendritically apposed varicosities release. (**L**) Airyscan image, dopamine axons innervating a cortical soma; soma-apposed varicosities release, nonapposed ones do not. (**M**) FOV showing tdTom^+^ dopamine neuron, immunostained for MAP2 (cyan), glial fibrillary acidic protein (GFAP; green), and 4′,6-diamidino-2-phenylindole (DAPI; yellow). (**N**) Enlarged ROI from (M): tdTom/DAPI/MAP2/Δ*F*/*F* (top) and tdTom/DAPI/GFAP/Δ*F*/*F* (bottom) overlays. Release occurs at MAP2^+^-apposed, but not GFAP^+^-apposed, varicosities. (**O**) Magnified ROI from (N): Varicosities near glia do not release despite proximity. (**P**) Representative traces, MAP2- versus GFAP-apposed varicosities (arrowheads, stimulation time). (**Q**) Averaged traces, MAP2- versus GFAP-apposed varicosities, *n* = 5 biological replicates. (**R**) AUC, MAP2- versus GFAP-apposed varicosities; points are means of 10 varicosities from *n* = 5 biological replicates. Means ± SEM. KS test; **P < 10^−2^.

First, we observed that dopamine release originates from a subset of varicosities, with considerable segments of dopamine axonal processes remaining release incompetent (quiescent) (Fig. 2, B and C). The release-competent (active) varicosities, which constituted a smaller proportion of varicosities within an imaging field of view (FOV; 34 ± 27% were active, means ± SD), were distinguished from quiescent varicosities because of their higher ∆*F*/*F* (14.7 ± 2.5% versus 3.65 ± 0.01%, means ± SEM; Fig. 2, D and E), larger area under the ∆*F*/*F* curve (3.25 ± 0.59 versus 0.42 ± 0.12, means ± SEM; Fig. 2F), and sharper, stimulation-locked responses (Fig. 2C, right). In contrast, quiescent varicosities exhibited smaller ∆*F*/*F* that often fell below the limit of detection, and, when dopamine signals were measured, they were characterized by long latency to peak (0.81 ± 0.1 s versus 4.55 ± 0.86 s, means ± SEM; Fig. 2G), suggesting that detected signals likely reflect diffusive spillover from neighboring release sites rather than local exocytosis. These characteristic differences were also observed during spontaneous (cell-autonomous) release events (fig. S3) and persisted even under vigorous stimulation conditions (fig. S3, B and C), indicating that the observed differences reflect intrinsic release competency differences between active and quiescent processes.

Second, active varicosities were spatially clustered, resulting in a distinctly nonuniform dopamine release profile across imaging fields (Fig. 2B and fig. S3A). To quantify this organization, we analyzed the distribution of pairwise distances between DA [i.e., tdTomato (tdTom)^+^] varicosities (boutons). The distribution of pairwise-distances of active boutons displayed a prominent peak at small interbouton distances (10.5 ± 7.9 μm, means ± SD) contrasting with the broad, featureless distribution of quiescent boutons and all DA varicosities (Fig. 2H). This clustering was consistent across both DA:C and DA:S cocultures (Fig. 2I). If release competence in dopamine axons is stochastically distributed, as is now understood, then the pairwise distribution of active boutons would be expected to resemble the overall distribution of interbouton distances. However, this is not observed in our data. Thus, this analysis suggested a nonarbitrary and structured arrangement of release competence in axonal varicosities of dopamine neurons.

Third, release competence strongly correlated with anatomical apposition to neuronal processes. Immunostaining for the somatodendritic marker MAP2 revealed that active boutons were preferentially localized near dendrites or soma of cocultured non-DA neurons (Fig. 2, K and L, respectively). Normalized ∆*F*/*F* amplitudes decayed with increasing distance from MAP2^+^ processes, with the highest release amplitudes occurring at sites of direct juxtaposition between varicosities and dendrites/soma in both DA:C and DA:S cultures (Fig. 2J).

We identified two predominant configurations of release-competent axons: those that were dendritically apposed (Fig. 2K) and those that are soma-apposed (Fig. 2L). Axosomatic appositions were more frequently observed in DA:C cocultures (72%, 36 of the 50 innervated cortical neurons had DA axons terminating on cell bodies and proximal dendrites), and we found that these innervations were often high-efficacy dopamine release sites (Fig. 2L and movie S1), whereas, in DA:S cocultures, the innervations were predominantly dendritic (70%, 35 of the 50 innervated striatal cells preferentially received dendritic DA input). Inspection of dopamine secretion along the contours of individual axonal processes revealed that proximity to and appositional contact with neuronal processes is a prerequisite for release competence (fig. S4, A and B). Notably, this spatial coupling was specific to neuronal processes; proximity to glial fibrillary acidic protein (GFAP)^+^ glia did not enhance release competence (Fig. 2, M to O, and fig. S4C), and MAP2-apposed boutons exhibited significantly stronger release than GFAP-associated sites (Fig. 2, P to R). Collectively, these data demonstrate that dopamine release is tightly coupled to structural apposition with neuronal targets, exhibiting an all-or-nothing propensity in which active varicosities display high release probability while nonapposed quiescent sites remain refractory.

### Dopamine axons selectively target dopamine receptor–expressing neurons to form release-competent appositions

Neurons in culture are highly synaptogenic, readily forming synapses with protein-coated beads or nonneuronal cells heterologously expressing postsynaptic molecules (*31*–*34*). We therefore asked whether the appositional contacts made by active dopamine axons occur nonspecifically with all neuronal types or are preferentially targeted toward processes expressing dopamine receptors. Unlike receptors for fast ionotropic ligands, which are broadly expressed, dopamine receptors are expressed in specific neuronal subtypes. Thus, the stereotyped and sparsely distributed axosomatic associations (e.g., Fig. 2L) observed between release-competent DA axons and discrete cortical neurons in our DA:C cocultures provided a suitable substrate to probe the specificity of appositional contacts formed by active DA varicosities.

To assess specificity, we first performed post hoc RNA fluorescence in situ hybridization (FISH) to profile dopamine receptor expression in DA:C cocultures. Dopamine signaling is primarily mediated by D1- or D2-type receptors, encoded by *Drd1* and *Drd2* genes, respectively. Using a modified RNAscope protocol (see Materials and Methods), we probed Drd1/2 transcripts and found that dopamine axons preferentially associate with soma containing Drd1 and/or Drd2 mRNA (Fig. 3, A to C and fig. S5). Specifically, we observed that dopamine axons exhibit a conspicuous tropism toward nuclei that were positive for *Drd1* mRNA and/or *Drd2* mRNA (fig. S5, A to C). To show that this innervation pattern was not random, we segmented all 4′,6-diamidino-2-phenylindole (DAPI)–stained nuclei that were colocated in imaging fields containing well arborized dopamine axons and measured distances between nuclei and the nearest DA axons. Here, DAPI-labeled nuclei were dilated to approximate somatic location (see Materials and Methods); this approximation was applied uniformly across all cell classes and was used to quantify cell-type–specific axonal tropism. Distribution analysis of these distances revealed that *Drd1*^+^ nuclei were significantly closer to dopamine axons than is observed in the overall population (Fig. 3D). Nearly all of the *Drd1*^+^ DAPI signals (97%) were found within 2 μm of DA axonal processes versus 56% for the overall population (Fig. 3D). Shuffling analysis confirmed that this correlation was unlikely to arise randomly (Fig. 3D). Furthermore, quantifying tdTom intensity relative to distance from DAPI-stained nuclei showed that *Drd1*^+^ cells exhibited stronger axonal innervation than the overall population (Fig. 3E). Notably, axosomatic innervations were more frequently observed at *Drd1*^+^ soma (16 of 27) compared with *Drd2*^+^-only soma (5 of 27), and *Drd1*^+^ nuclei received more intense axonal innervation than *Drd2*^+^-only nuclei. Together, these results indicate a clear bias in appositional contacts, with active varicosities targeting receptor-mRNA–expressing cells and further showed subtle distinctions in innervation pattern between *Drd1*- and -*Drd2* mRNA–expressing cell populations.

**Fig. 3.**
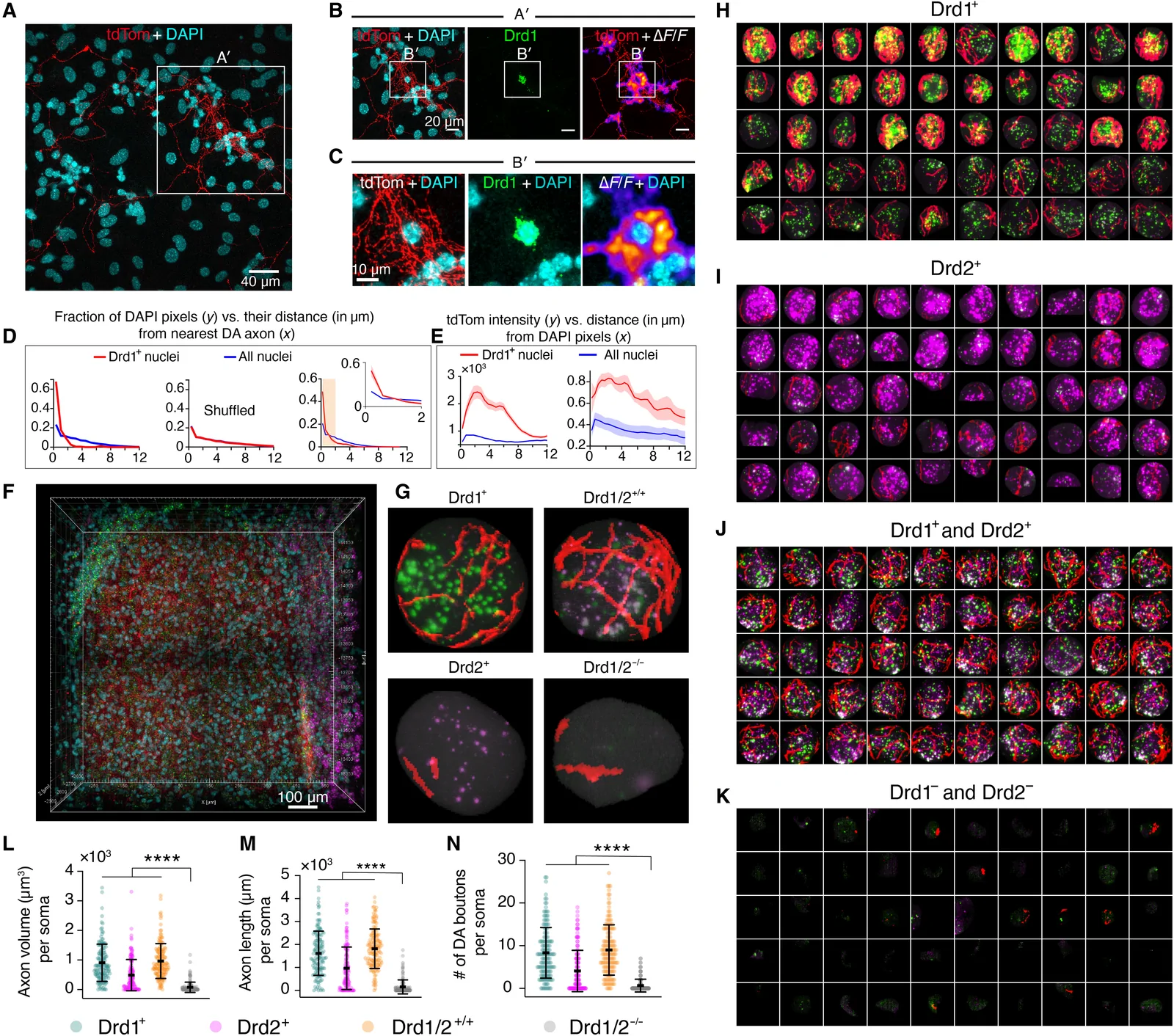
Dopamine axons target dopamine receptor–mRNA–expressing neurons to form release-competent appositional contacts. (**A**) A FOV showing DA axons (red, tdTom) and DAPI (cyan), showing axon convergence toward the cell at the center of the ROI (white box, A′). (**B**) ROI (A′) expanded as a three-panel figure: tdTom^+^ DAPI, *Drd1* signal (RNAscope), and tdTom^+^ Δ*F*/*F* showing dopamine release, highlighting tropism of tdTom processes (DA axons) toward the *Drd1*^+^ soma and robust dopamine secretion around it. (**C**) Close-up of ROI (B′), highlighting a “corona” of extracellular dopamine around the *Drd1*^+^ soma. (**D**) Left: Distance analysis shows that convergence of dopamine axons onto *Drd1*^+^ soma is unlikely due to chance. Fraction of DAPI^+^ pixels plotted versus distance from the nearest tdTom pixel shows that DAPI pixels from *Drd1*^+^ cells (red) differ markedly from the overall population (blue), with 97% of *Drd1*^+^ signal within 2 μm of a tdTom process versus 56% overall (*P* < 10^−4^, two-sample KS test). Middle: Shuffling analysis (tdTom randomly translated over 100 trials, DAPI static) shows no difference between *Drd1*^+^ and all cells. Right: Proximity analysis pooled from *n* = 6 biological replicates (inset: first 2 μm on the *x* axis). (**E**) tdTom intensity analysis shows that Drd1^+^ nuclei receive stronger DA innervation. Left: FOV in (A). Right: Normalized and pooled from *n* = 6 replicates. (**F**) Representative EASI-FISH volume in amygdala: dopamine axons (red), DAPI (blue), and *Drd1*/*Drd2* mRNA puncta (green/magenta). (**G**) DA axonal innervation of representative nuclei from each class. (**H** to **K**) Machine learning–based classification of all nuclei in (F) into *Drd1*^+^, *Drd2*^+^, *Drd1/2*^+/+^, and *Drd1/2*^−/−^ classes (*n* = 150 per class; 50 shown here). (**L** to **N**) Quantification of dopamine axon innervation across the four classes (*n* = 150 nuclei). Each point is a nucleus, dilated to approximate soma location (see Materials and Methods): total innervation parameters calculated per nucleus. KS test; ****P < 10^−4^, *Drd1/2*^−/−^ versus each other class. Means ± SD are shown.

We next asked whether DA axons can release transmitter when they contact somata lacking dopamine receptor transcripts. In fields where dopamine axons formed discrete axosomatic “coronas” around individual neurons, RNAscope showed that dopamine release was restricted to somata-expressing *Drd1* and *Drd2* transcripts (fig. S5, D and E). Within the same FOVs, nearby axons that contacted receptor-negative soma or processes showed no detectable release and remained quiescent (fig. S5F). Thus, physical proximity or comingling of dopamine axons with neuronal membranes is not sufficient to confer release competence; instead, release competence emerges selectively at contacts onto receptor-expressing neuronal processes.

While transcript-level mapping defines the molecular identity of target neurons, it does not establish receptor functionality or membrane localization. To verify that dopamine receptors are not only transcribed but also present and active at the membrane, we directly measured receptor-mediated signaling in “postsynaptic” partners. Fast synapses are defined by rapid ionic currents that confirm their functional coupling; by analogy, we asked whether appositionally engaged active dopamine varicosities evoke a biochemical counterpart (that is, a receptor-dependent cAMP response) in their targets.

To obtain spatiotemporal coupling between dopamine release and cAMP modulation, we performed dual-color imaging of dopamine and cAMP using viral transduction of the intensity-based cAMP sensor G-Flamp1 (*35*). We then examined the degree of colocalization between dopamine release events and cAMP dynamics. We found that cAMP activity in postsynaptic partners was spatiotemporally correlated with dopamine release (fig. S6). We observed clear dopamine receptor–mediated increases in cAMP, with cAMP signals lagging dopamine release by 4.3 ± 2.3 s (means ± SD) in *Drd1*^+^ neurons (fig. S6, A to C). In *Drd2*-expressing cortical neurons receiving axosomatic dopamine input, we detected a temporally correlated decrease in cAMP across soma and proximal dendrites (fig. S6, D to G). Receptor identity determined by post hoc RNAscope was consistent with the direction of cAMP modulation (negative) measured by G-Flamp1 (fig. S6E). While cAMP activity in soma and primary dendrites was largely synchronized, distal dendritic branches displayed localized hotspots of cAMP activity that aligned spatially with dopamine release sites visualized on DopaFilm (fig. S6H and I).

We next asked whether receptor-specific targeting of DA axons is also observed in intact brain tissue. Because axosomatic innervations were observed more frequently in DA:C cocultures than in DA:S, we first examined mesocorticolimbic axons from VTA dopamine neurons projecting to the amygdala (including BLA and central amygdala). We combined transcriptomic profiling (HCR) of dopamine receptor expression with expansion microscopy (ExM) for high-resolution imaging (EASI-FISH) (*36*). EASI-FISH couples tissue expansion with multiplexed in situ hybridization to map gene-expression–defined cell types and their spatial relationships to axons in intact tissue, making it well suited to relate receptor transcript identity to local DA innervation (Fig. 3F). The amygdala provided robust VTA-derived DA innervation and a balanced representation of *Drd1/2*-positive and *Drd1/2*-negative neurons, enabling a sensitive test of receptor-associated wiring specificity in relatively sparse projections. In parallel, we extended this approach to the dorsal striatum, where dense, fine DA fibers arising largely from SNc neurons course through a neuropil rich in *Drd1/2*-expressing neurons. Together, these two anatomical regions, with moderately innervated amygdala with mixed receptor expression and densely innervated striatum with high *Drd1/2* cell density, allowed us to assess receptor-specific targeting across distinct DA projection systems.

Using EASI-FISH, we segmented all DAPI-stained nuclei in amygdala and used machine learning to classify them into *Drd1*^+^, *Drd2*^+^, *Drd1*^+^, and *Drd2*^+^ double-positive *Drd1/2*^+/+^, or receptor-negative *Drd1/2*^−/−^ subgroups (Fig. 3G). As in the coculture analysis, DAPI volumes were uniformly dilated to approximate soma location for quantification of dopamine axon innervation. In line with previous studies, a substantial fraction (73%, 1840 of the 2521 neurons) of amygdala neurons expressed *Drd1* and/or *Drd2* (*37*). Soma-expressing *Drd1* alone (12%) or both *Drd1* and *Drd2* (13%) received the strongest axonal innervation, with dopamine axons forming pericellular basket innervations around such soma (Fig. 3, H and J). These innervations were less intense in *Drd2* neurons (Fig. 3I) and, critically, were not observed in neurons lacking *Drd1* and *Drd2* (27%, Fig. 3K). We quantified these observations by measuring total axonal volume per soma, contour length of soma-associated axons, and ramification (number of boutons). Across all metrics, *Drd1*- and *Drd2*-expressing cells were more heavily innervated than receptor-negative neurons (Fig. 3, L to N). Specifically, total axonal volume was highest around *Drd1*^+^ soma (905 ± 51 μm^3^) and *Drd1/2*^+/+^ soma (964 ± 48 μm^3^), remained robust but reduced around *Drd2*^+^ soma (490 ± 43 μm^3^), and was sharply lower around *Drd1/2*^−/−^ soma (78 ± 14 μm^3^) (all means ± SEM). Similarly, total length of soma-associated axons followed the same pattern, with 1616 ± 79 μm around *Drd1*^+^ soma, 1814 ± 70 μm around *Drd1/2*^+/+^ soma, 964 ± 75 μm around *Drd2*^+^ soma, and 154 ± 25 μm around *Drd1/2*^−/−^ soma (all means ± SEM). We also segmented axonal processes to identify boutons: *Drd1*^+^ and *Drd1/2*^+/+^ soma exhibited more boutons (8.3 ± 0.48 and 9.0 ± 0.48, respectively), compared with 4.1 ± 0.40 around *Drd2*^+^ soma and 0.7 ± 0.12 around *Drd1/2*^−/−^ soma (all means ± SEM). Consistent with our observations in culture, the density of innervation was markedly lower in *Drd2*^+^ soma compared with *Drd1*^+^ soma. 

Extending this analysis to the dorsal striatum, we applied EASI-FISH to segment all DAPI-stained nuclei and similarly classified neurons into *Drd1*^+^, *Drd2*^+^, *Drd1/2*^+/+^, or receptor-negative (*Drd1/2*^−/−^) subgroups (fig. S7). In this region, dopamine axons formed pervasive fine-caliber arborizations throughout the neuropil, yet receptor specificity was still preserved: Soma classified as *Drd1*^+^ or *Drd2*^+^ showed the strongest soma-associated innervation, whereas receptor-negative neurons exhibited markedly reduced soma-associated axonal coverage (fig. S7). Quantification of perisomatic axonal volume, contour length, and bouton number confirmed significantly higher innervation of *Drd1/2*-expressing soma relative to *Drd1/2*^−/−^ cells (fig. S7), indicating that receptor-driven tropism of DA axons generalizes from the sparser VTA-to-amygdala projection to the dense SNc-to-striatal projection system. Notably, when comparing EASI-FISH datasets across the two regions, two differences stand out. First, unlike the amygdala, *Drd1/2* double-positive soma was rarely observed in the striatum (Fig. 3, L to N versus fig. S7). Second, whereas *Drd2*^+^ cells in the amygdala were less robustly innervated than *Drd1*^+^ cells, *Drd2*^+^ cells in the striatum received innervation comparable to *Drd1*^+^ cells. This regional difference may reflect anatomy-, receptor-, and cell-type–specific mechanisms that differentially shape DA innervation patterns across terminal fields. In summary, dopamine axons exhibit strong tropism toward receptor-expressing neurons with which they form appositional contacts. This receptor-dependent innervation is evident in both dissociated cultures and intact tissue and is tightly linked to release competence in vitro. These findings suggest that DA varicosities align their output with target neurons whose transcriptomic profiles support dopamine receptor expression.

### Visualizing receptor nanoscale organization with ALFADoR mice

While transcriptomic profiling revealed axonal tropism and wiring specificity in dopamine axonal outgrowth, and cAMP activity imaging suggests the presence of compartmentalized signaling at the subcellular level, it does not directly address the spatial relationship between dopamine receptors and dopamine release sites. To resolve this, it is necessary to map dopamine receptor distribution with subcellular resolution. However, the subcellular distribution of dopamine receptors, which are G protein coupled, is poorly characterized, partly due to the lack of reliable reagents compatible with immunolabeling, a known and vexing issue for the field (*38*). To overcome this limitation, we focused on the two predominant dopamine receptors, D1 and D2, and generated transgenic mice in which one copy of the ALFA-tag (*39*) was knocked into the N terminus of either receptor using CRISPR-mediated gene editing (Fig. 4A) (Materials and Methods). We confirmed precise ALFA-tag insertion at the target gene loci by polymerase chain reaction (PCR) genotyping and Sanger sequencing. KI animals showed the expected 5′ and 3′ junction amplicon sizes [wild-type (WT) versus KI fragments], confirming correct homology-directed integration (figs. S8 and S9). Sanger chromatograms spanning both junctions showed single, high-quality reads with good peak resolution and complete continuity across the genome-to-ALFA and ALFA-to-genome boundaries on both ends (figs. S8 and S9). Alignment of the reads to the reference sequence showed an in-frame fusion without insertions, deletions, or other sequence deviations, verifying an error-free ALFA-tag KI at the intended locus. In vitro, mRNA for each gene corresponded with ALFA-tag positive cells, confirming specificity (figs. S8 and S9). To determine whether ALFA-tag insertion altered receptor abundance, we performed quantitative liquid chromatography–tandem mass spectrometry (LC-MS/MS) proteomic analysis of striatal lysates from WT, ALFADoR1, and ALFADoR2 mice. D1 receptor abundance was comparable across genotypes after normalization to a panel of striatal control proteins spanning surface-associated and cytoplasmic compartments, indicating that ALFA-tag insertion does not measurably alter total D1 receptor expression (fig. S10). D2 peptides were not detected with sufficient confidence in these whole-striatal lysates to permit equivalent quantitative analysis. We refer to these animals as ALFADoR mice (ALFA-tagged dopamine receptor mice; ALFADoR1 for *Drd1* KI and ALFADoR2 for *Drd2* KI).

**Fig. 4.**
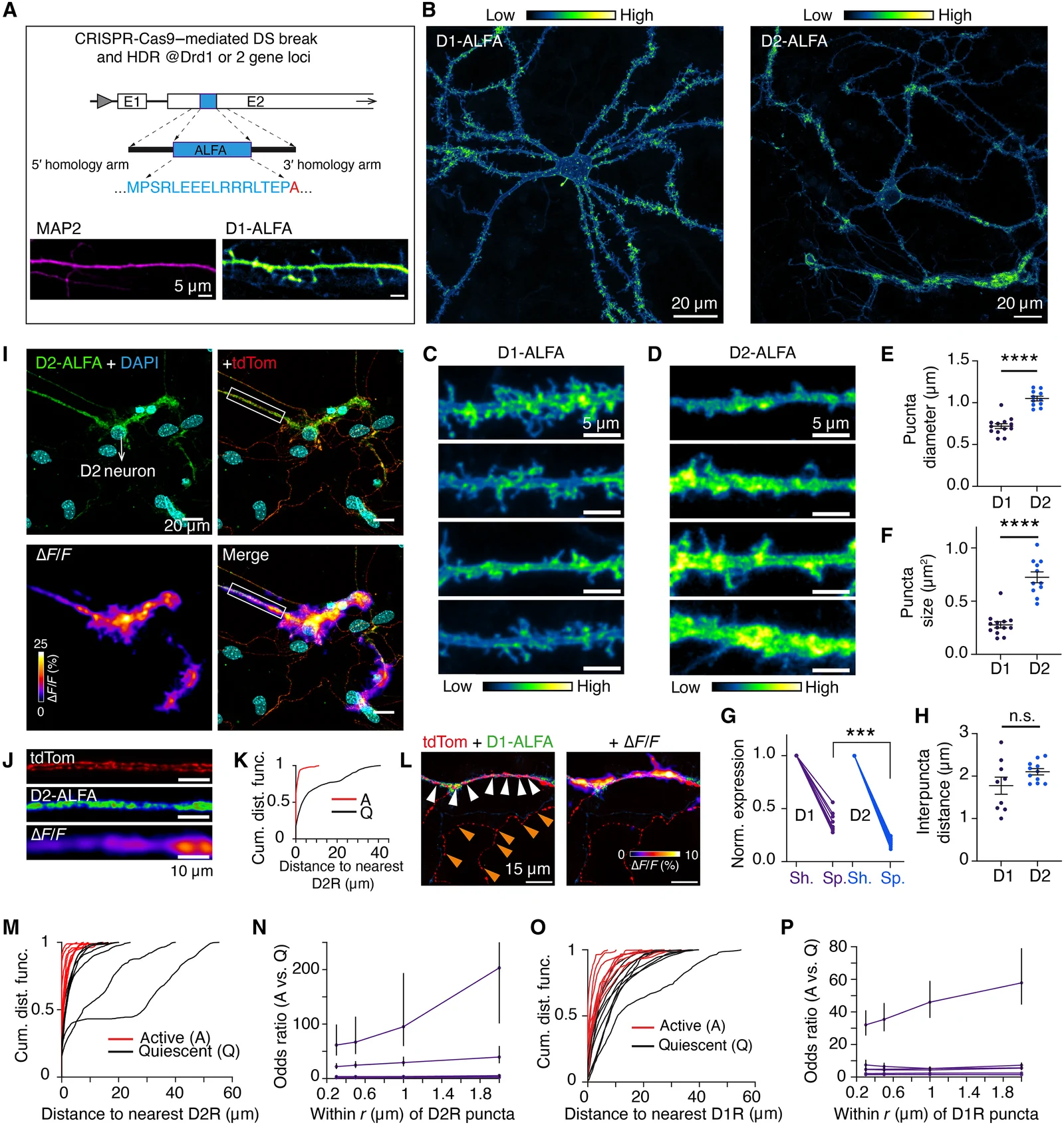
The subcellular distribution of D1 and D2 dopamine receptors, visualized with ALFADoR KI mice. (**A**) Design of ALFADoR mice. Bottom: MAP2^+^ dendritic process of a *Drd1*^+^ neuron and its D1-ALFA readout, showing receptor enrichment in shaft and spines. (**B**) Pan-neuronal live-cell visualization of D1-ALFA and D2-ALFA expression at subcellular resolution (anti–ALFA-tag nanobodies, Atto643). (**C** and **D**) Representative D1-ALFA and D2-ALFA expression, four dendritic segments. (**E** to **F**) D1-ALFA and D2-ALFA puncta size (area and characteristic linear scale reported as “diameter”); each point is mean size per neuron; means ± SEM. KS test; ****P < 10^−4^. (**G**) Expression intensity in dendritic shafts (Sh.) versus spines (Sp.), normalized to shaft; each point is a segment average. D2-ALFA spine intensity is low relative to D1-ALFA (18.5% versus 38% of shaft; KS test; ***P < 10^−3^). (**H**) Interpuncta distance of D1- and D2-ALFA signals; each point is a neuron mean; means ± SEM. KS test; n.s., not significant. (**I**) DA axons (tdTom) arborizing in a FOV with a D2-ALFA^+^ neuron. Dopamine Δ*F*/*F* tracks D2-ALFA expression; only receptor-apposed axons release dopamine. (**J**) Close-up of the ROI in (I): apposition of active varicosities and D2-ALFA puncta. Scale bars, 10 μm. (**K**) Nearest-receptor distance distribution of active (A) and quiescent (Q) varicosities from (I): Active varicosities lie much closer to D2-ALFA puncta. (**L**) Dopamine release at D1-ALFA-apposed axons (tdTom, red): D1 puncta are apposed to active varicosities (white arrowheads), and not quiescent ones (orange arrowheads). (**M** to **P**) Distances between active (A) and quiescent (Q) varicosities and receptor puncta. Cumulative distributions for A and Q are shown in (M) and (O); *n* = 7 (D2) and *n* = 9 (D1) paired traces. (N and P) OR: Odds of finding A versus Q within “*r*” μm of a receptor punctum; *y* > 1 indicates enrichment near receptors. All traces lie above *y* = 1; bars are 95% bootstrap confidence intervals (CIs; see Materials and Methods).

Using ALFADoR mice, we visualized the subcellular distribution of dopamine receptors in cortical and striatal cultures. In both cultures, dopamine receptors exhibited a punctate distribution (Fig. 4, B to D). D1 receptors formed puncta with mean characteristic length scales of 0.72 ± 0.03 μm and average area of 0.28 ± 0.03 μm^2^, whereas D2 receptor puncta were larger, averaging 1.05 ± 0.03 μm in linear scale and 0.72 ± 0.05 μm^2^ in area (means ± SEM for all) (Fig. 4, E and F). Dopamine receptor puncta were observed in both spiny neurons (predominantly striatal) and aspiny neuron types (predominantly cortical) (fig. S11). D1 puncta were observed in both dendritic shafts and dendritic spines, with puncta intensity in spines being ∼38 ± 3% (means ± SEM) of those in shafts (Fig. 4G and fig. S11, A and B). In contrast, D2 puncta were primarily localized in dendritic shafts, with intensity of D2 expression in spines being ∼18.5 ± 1.1% (means ± SEM) of those in shafts, markedly lower compared with D1 intensity in spines (Fig. 4G). The mean interpuncta distance in neuronal subregions that express dopamine receptors was 1.78 ± 0.2 μm for D1 and 2.1 ± 0.07 μm for D2 (Fig. 4H and fig. S12). We were able to visualize dopamine receptors in live-cell imaging using fluorescent anti–ALFA-tag nanobodies, confirming the N terminus locus and extracellular accessibility of the tag (fig. S11, C and D) and performed cAMP imaging to confirm receptor functionality (fig. S11, E and F). Collectively, these data indicate that dopamine receptors exhibit a structured and heterogeneous subcellular distribution rather than uniform membrane dispersion. This patterning suggests that GPCR-mediated neuromodulation may be organized at a finer subcellular scale than previously recognized.

With this context, we examined how release-competent (active) varicosities are organized relative to dopamine receptor puncta. Here, DA neurons were cultured from the standard *Slc6a3*-Cre x Ai9 line, and the striatal and cortical cocultures were produced from ALFADoR1 or ALFADoR2 mice. In mixed cocultures and consistent with the tropism observed with RNA FISH data, we found that dopamine axons preferentially innervate dopamine receptor–expressing processes, forming close appositional contact between active varicosities and receptor puncta (Fig. 4, I to L, and fig. S13).

To quantify these associations, we calculated the distance between all dopamine axonal varicosities (active and quiescent) and the nearest D1 or D2 receptor puncta. We performed two analyses on these distances: (i) the cumulative distribution function (CDF) of the distances between varicosities and receptor puncta and (ii) the odds ratio (OR) of finding an active or quiescent varicosity within the vicinity of dopamine receptor puncta (an OR > 1 indicates enrichment of active varicosities near receptor puncta relative to quiescent ones). Using the first metric, we found that active varicosities, as a population, lie closer to dopamine receptor puncta than inactive processes (Fig. 4, M and O). For example, the CDF showed that 75 ± 6% of all active varicosities were within 1 μm of a D2 receptor punctum, compared with 41 ± 4% of quiescent varicosities (Kolmogorov-Smirnov test, *P* = 0.0006; means ± SEM; Fig. 4M). Similarly, 45 ± 6% of all active varicosities were within 1 μm of a D1 receptor punctum, compared with 20 ± 4% of quiescent varicosities (Kolmogorov-Smirnov test, *P* = 0.034; means ± SEM; Fig. 4O).

Using the second metric, we similarly found that the OR of finding an active varicosity within “*x*” (*x* = 0.2, 0.3, 1, and 2 μm) radius of a receptor puncta was well above 1 (Fig. 4, N and P); (at *x* = 1 μm, OR = 7.85 ± 4.8 for D1-ALFA and 15.7 ± 10.4 for D2-ALFA; means ± SEM for both). These evaluations demonstrate a spatial alignment between dopamine release sites and receptor-rich domains that cannot be accounted for by chance alone. Integrating these proximity data with RNA-FISH mapping of receptor expression and EASI-FISH analyses in the amygdala and striatum, our results indicate that dopamine axons are biased toward receptor-expressing targets, and release sites are anatomically apposed to receptor clusters.

### Dopamine-releasing varicosities are enriched with specializations that facilitate temporally precise neurotransmitter release

Given the proximity between dopamine release sites and receptor clusters, we next asked whether release-competent varicosities have molecular specialization characteristics of classical presynaptic terminals. Using immunofluorescence microscopy, we compared active and quiescent varicosities to determine whether active sites exhibit a molecular architecture consistent with synaptic organization. Synaptophysin (Syp) staining revealed that appositional contacts formed by active varicosities were enriched in Syp, consistent with vesicle clustering at the interface and suggesting the presence of a synapse-like presynaptic specialization at these sites (Fig. 5, A and B). Encouraged by this observation and by previous reports showing that dopamine release sites contain cytomatrix proteins found in fast synapses (*26*, *40*), we examined additional presynaptic proteins.

**Fig. 5.**
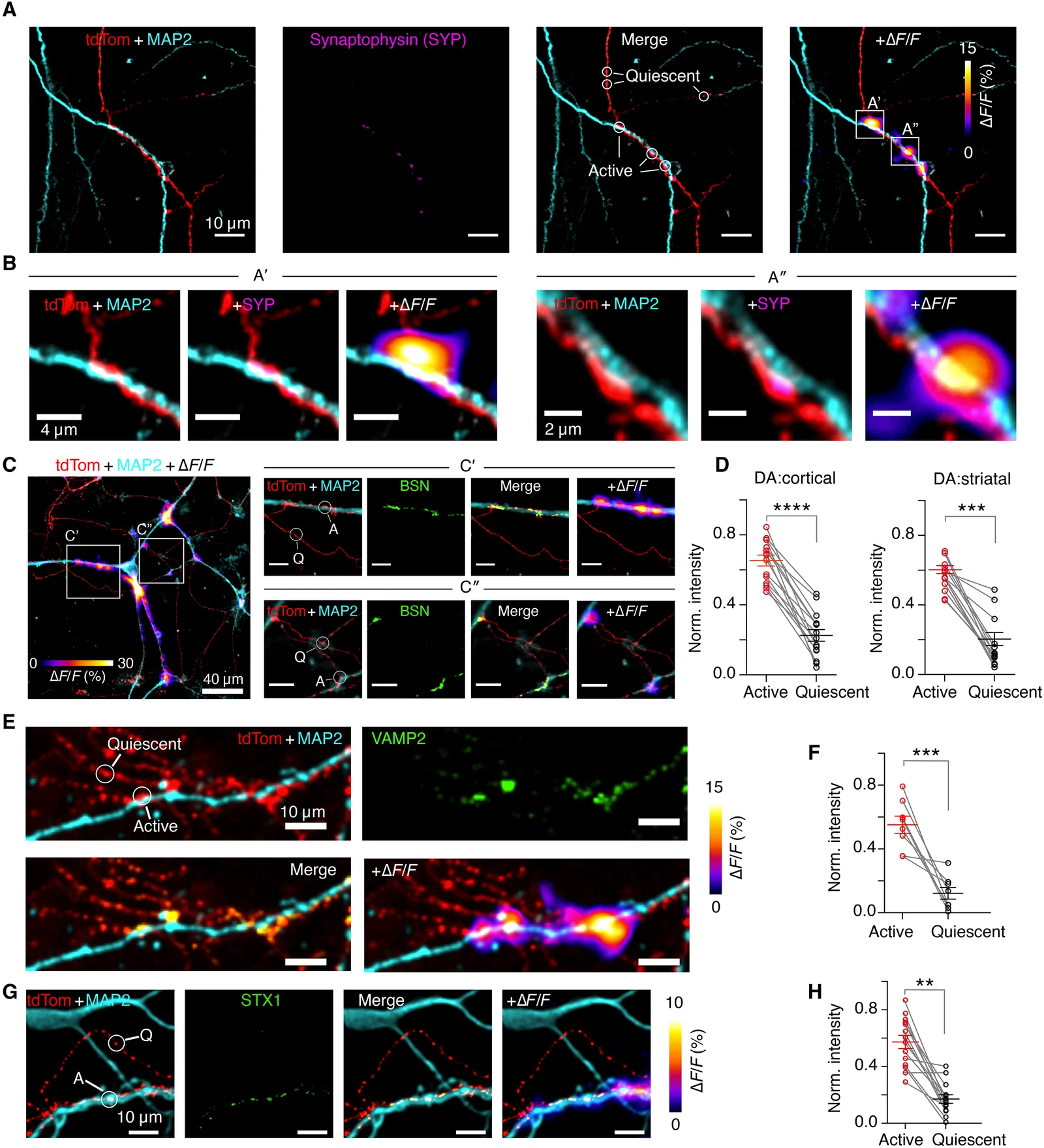
Assessment of synaptic release machinery at active versus quiescent varicosities. (**A**) Dopamine axonal process (tdTom) innervating a dendrite (MAP2), synaptophysin (SYP) readout from the same FOV, overlaid with dopamine release activity measured on DopaFilm (Δ*F*/*F*). (**B**) Close-up of ROIs A′ and A″, showing enrichment in SYP (magenta) at the interface between the red (tdTom, DA axon) and cyan (MAP2, dendrite) channels. (**C**) Dopamine neuron axonal processes (red, tdTom) innervating a MAP2-immunostained cortical neuron (cyan), overlaid with dopamine release activity measured on DopaFilm (Δ*F*/*F*). Right: The ROIs shown in white boxes (C′ and C″) are expanded; bassoon (Bsn) expression is shown in green, overlaid with dopamine release (Δ*F*/*F*). Representative active (A) and quiescent (Q) varicosities are depicted. (**D**) Bsn expression assayed at active and quiescent varicosities. Each point is the mean from a biological replicate, averaging Bsn expression at 10 randomly selected active and quiescent varicosities from the same sample. Data are normalized against the highest-intensity varicosity and averaged; means ± SEM bars are shown. Significance evaluated with a nonparametric two-sample KS test; ****P < 10^−4^; ***P < 10^−3^. (**E**) Dopamine release from an axonal arbor innervating a dendritic process (MAP2, cyan), and synaptobrevin 2 immunofluorescence readout (Vamp2, green). Representative active and quiescent varicosities are depicted. (**F**) Synaptobrevin 2 (Vamp2) expression at active versus quiescent varicosities, quantified as in (D). Significance evaluated with a nonparametric two-sample KS test; ***P < 10^−3^. (**G**) Dopamine release from an axonal process innervating dendritic processes (MAP2), and syntaxin 1 immunofluorescence readout (Stx1, green). Representative active (A) and quiescent (Q) varicosities are depicted. (**H**) Stx1 expression at active versus quiescent varicosities, quantified as in (D). Significance evaluated with a nonparametric two-sample KS test; **P < 10^−2^.

At conventional synapses, the scaffolding protein bassoon (Bsn) is concentrated at the active zone and is among the first molecules recruited during presynaptic assembly (*41*, *42*). Using immunofluorescence microscopy, we found that Bsn expression strongly correlated with release competency in dopamine varicosities (Fig. 5C). Bsn levels were significantly higher in active compared to quiescent varicosities across both DA:C (0.65 ± 0.03 versus 0.22 ± 0.03, means ± SEM on normalized data) and DA:S cocultures (0.57 ± 0.02 versus 0.17 ± 0.04, means ± SEM on normalized data) (Fig. 5D), and Bsn expression correlated with dopamine release amplitude (∆*F*) (fig. S14, A and B). The correspondence between DopaFilm hotspots and Bsn-enriched varicosities was precise, especially at nonbranching axons (Fig. 5C). At branch points or where dopamine axons innervate dendritic shafts, varicosities were ramified and produced merged hotspots in DopaFilm recordings [compare fig. S14 (C and D) and fig. S14 (C and E)]. Although individual hotspots were not visually resolvable in such clusters, nonnegative matrix factorization (NNMF) analysis (see Materials and Methods) successfully separated overlapping signals, and the computationally resolved hotspots colocalized with individual or clustered Bsn puncta in immunofluorescence images (fig. S14E).

Other presynaptic proteins essential for exocytosis were similarly enriched at active varicosities. The v-SNARE protein synaptobrevin 2 (Vamp2) and the t-SNARE Syntaxin 1 (Stx1) were both enriched relative to quiescent varicosities (0.55 ± 0.05 versus 0.12 ± 0.04, means ± SEM on normalized data) (Fig. 5, E to H). The vesicle-priming protein Munc13-1 showed preferential enrichment in active varicosities (fig. S14, F and G). Rims1/2 (RIM), a scaffolding protein previously shown to be critical for dopamine release (*26*, *43*), was likewise enriched at active sites (fig. S14, H and I).

To test whether these molecular specializations are required for dopamine release, we performed RNA interference–mediated knockdown (KD) of Bsn, Rim, Stx1, Syt1, Vamp2, and Munc13-1 in our coculture assays (fig. S15, A and B). Using quantitative proteomics, we confirmed efficient depletion of each protein target (fig. S15, C and D). Munc13-1 and RIM KD efficiencies were the highest, showing near complete depletion, whereas the remaining targets were depleted with efficiencies ranging from 68% (Bsn) to 98% (Vamp2) (fig. S15, C and D). In each KD preparation, we measured evoked dopamine release and showed that it was nearly completely abolished for Munc13-1, RIM, Stx1, and Syt1 KD (fig. S15, E and F). Bsn KD reduced the extent of dopamine release but did not completely eliminate it (fig. S15F). In contrast, we did not observe any diminution in evoked dopamine release in cells transduced with scrambled control short hairpin RNA (shRNA; fig. S15F).

Because previous studies have reported that DA terminals in the striatum are preferentially apposed to neuroligin-2 (NL-2)–positive postsynaptic specializations (*17*, *19*), we asked whether functionally active dopamine varicosities identified in our assay exhibited a similar molecular organization. Immunostaining of DA:S cocultures revealed that many Bsn-positive DA varicosities were closely apposed to NL-2 puncta (fig. S16, A to C). Further analysis showed that 26 of 50 (52%) and 30 of 50 (60%) active varicosities in DA:C and DA:S cocultures, respectively, were located within ∼300 nm of the nearest NL-2 punctum in diffraction limited images, indicating that a substantial fraction of release-competent dopamine boutons form appositions onto NL-2–positive postsynaptic domains (fig. S16D). These observations are in agreement with previous studies and further support the notion that functionally active dopamine varicosities exhibit synapse-like molecular organization. A substantial fraction of active varicosities did not exhibit close association with NL-2 puncta, suggesting that additional postsynaptic adhesion molecules or scaffolding complexes may participate in organizing dopamine release sites.

Together, these findings show that release-competent dopamine varicosities harbor a canonical presynaptic molecular architecture. Their enrichment in Bassoon, Munc13-1, Rim, Vamp2, Stx1, and Syt1 mirrors that of fast chemical synapses, and targeted disruption of these proteins functionally silences dopamine release. Together with receptor organization and FISH-based axonal tropism, these results reveal that dopamine axons have synapse-like structural and molecular specializations and wiring specificity that confer precision and efficiency to dopamine signaling.

### Ultrastructural assessment of dopamine-releasing varicosities in contact with dendrites and soma

To further characterize the structural basis of dopamine release, we examined the ultrastructure of release-competent (active) varicosities that formed appositional contacts with neuronal targets. Because dopamine neurons express Cre recombinase in our coculture system (*Slc6a3*-Cre x Ai9), we used a cytosol-localized pAAV–EF1a-DIO-APEX2.NES construct (*44*) to label DA varicosities and correlate their ultrastructure with Δ*F*/*F* activity (Fig. 6). Viral expression levels were titrated such that DA processes exhibited enhanced electron density under transmission electron microscopy (TEM) while preserving ultrastructural detail (Fig. 6C and fig. S17A). Cocultures were grown on gridded DopaFilm substrates, enabling us to identify the same imaging fields in live-cell imaging and electron microscopy sessions (Fig. 6A). Following live-cell imaging, cultures were fixed and processed for serial-section TEM (section thickness, 50 nm). Low-magnification electron micrographs were first used to align light and TEM datasets, and DAB reaction product provided positive identification of DA (DAB^+^) varicosities.

**Fig. 6.**
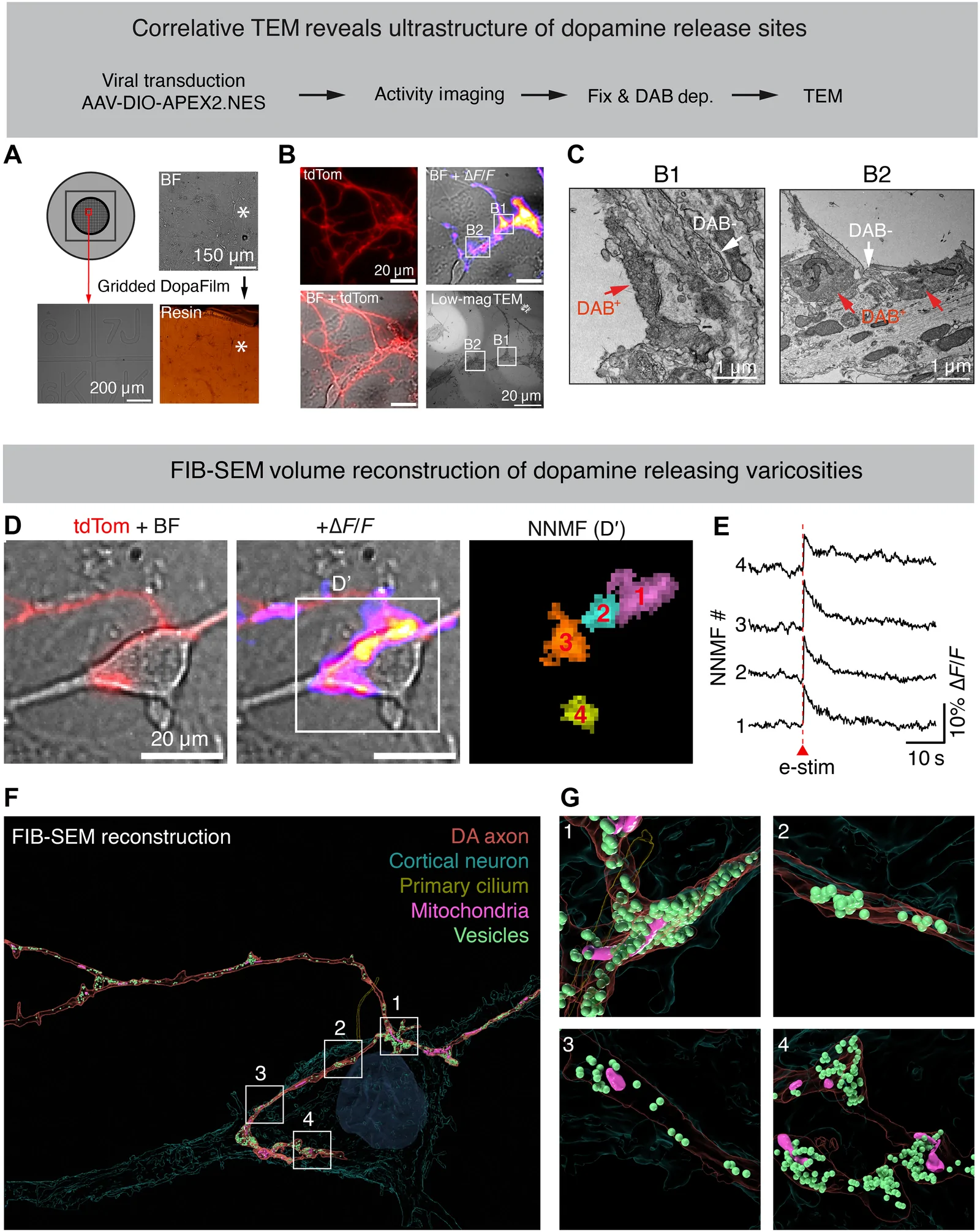
Activity-guided ultrastructural characterization of dopamine release sites near dendrites and soma of target neurons. (**A**) Schematic of a gridded (alphanumerically patterned) cell culture dish, where neurons are sparsely cocultured on DopaFilm. After dopamine release sites are recorded in live-cell imaging, the specimen is fixed and processed for EM (resin) (see Materials and Methods). The ROI is identified in the resin and trimmed. Thin serial sections collected from the resin blocks are imaged using TEM. To identify DA varicosities, virally transduced APEX2-based DAB deposition is used. (**B**) DA axonal processes innervating a dendrite of a cortical neuron shown in bright field (BF). Dopamine ∆*F*/*F* is from live-cell imaging on DopaFilm, and the corresponding low-magnification (low-mag) TEM of the same FOV is also shown. (**C**) Close-up of ROIs B1 and B2 depicted in (B), showing DAB^+^ varicosities lying in apposition against a dendritic process. DAB^−^ varicosities are also observed in the same field. (**D** and **E**) Dopamine axons innervating a soma, with NNMF-segmented dopamine release hotspots and corresponding Δ*F*/*F* activity traces. (**F** and **G**) FIB-SEM volume reconstruction of the FOV shown in (D), highlighting dopamine axons, varicosities, and mitochondria.

We first surveyed DA and non-DA boutons within the same imaging fields and performed morphometric analysis using a previously published method (fig. S17B) (*45*). DA (DAB^+^) varicosities contained numerous small, clear vesicles spread out over an average area of 0.14 μm^2^ per varicosity, compared with 0.07 μm^2^ in non-DA (DAB^−^) boutons. Dopamine varicosities harbored more vesicles compared with non-DA boutons (90 ± 52 versus 48 ± 26 per bouton, *n* = 15; means ± SD) while retaining similar mean intervesicle distances (center to center, 56 ± 9 nm versus 56 ± 7 nm per bouton, *n* = 15; means ± SD), indicative of similar vesicle clustering (fig. S17C). Consistent with this observation, vesicles occupied 19.5 ± 6.5% of total varicosity volume in DA boutons compared with 17.0 ± 5.4% (means ± SD for both) in non-DA boutons (fig. S17C). Classical synaptic ultrastructure defined by parallel pre- and postsynaptic membranes separated by a prominent cleft was observed in 60% of non-DA varicosities but only 40% of dopamine varicosities. Instead, most dopamine varicosity appositional contacts were characterized by narrow, undulating interfaces lacking a well-defined cleft (Fig. 6C and fig. S17D). Using correlated light and electron microscopy, we confirmed that DAB^+^ varicosities colocalized with Δ*F*/*F* release hotspots, linking this morphology directly to functional activity (Fig. 6, B and C). These observations indicate that dopamine varicosities form contacts whose ultrastructure somewhat deviates from canonical synapses but retain key synaptic hallmarks such as vesicle clustering and apposition against soma or dendrites.

We further characterized the appositional partners of DA varicosities across DA:C and DA:S cocultures (fig. S17D). Survey of 58 serial sections from DA:C and 68 sections from DA:S cocultures revealed that spine appositions were more frequent in DA:S than in DA:C (fig. S17E). Notably, active DA boutons contacting dendritic spines exhibited classical synaptic ultrastructure in 56% of cases, compared with 47% for shaft and somatic contacts. Last, comparison of active and quiescent varicosities revealed ultrastructural differences: Quiescent boutons contained fewer, more loosely packed vesicles and showed greater vesicle pleomorphism (fig. S17, F to H), although their overall varicosity size was similar (fig. S17I).

To extend our EM analysis to three dimensions, we performed volumetric reconstruction of a dopamine axonal process apposed to a soma in a DA:C coculture via focused ion beam scanning electron microscopy (FIB-SEM) (Fig. 6, D to G). This reconstruction enabled visualization of vesicle organization throughout the axonal process and within individual varicosities, rather than in isolated ultrathin sections. In reconstructed axons, vesicles appeared sparsely entrained along much of the dopamine axon. However, vesicle distribution was not uniform and instead formed local clusters at discrete regions. These vesicle clusters colocalized with dopamine hotspots identified in activity recording sessions (Fig. 6, D to G). Mitochondria were also colocated with vesicle-rich regions; however, this observation was based on a single reconstructed volume and could not be used for quantification. The FIB-SEM volume further allowed us to follow the continuity of the DA axon and examine the 3D geometry of its apposition to the target soma (movie S2). These data provide volumetric view of active varicosities and their appositional contacts with target processes and are consistent with the notion that release-competent sites are organized as discrete vesicle-rich appositional contacts along DA processes.

Together, EM analyses define active dopamine varicosities as structurally specialized release sites, with vesicle-rich axonal domains that engage neuronal targets through appositional contacts, vary in ultrastructure according to the target and release-competence, and retain key presynaptic features despite often lacking the stereotyped architecture of classical synapses.

### Spatial organization of dopamine receptor microdomains in intact brain tissue

Based on the evidence that dopamine axons contain presynaptic specializations consistent with synapse-like release sites and that dopamine axons exhibit a tropism for receptor-expressing processes, we next asked whether endogenous dopamine receptors are organized in intact tissue in a manner that enables spatially restricted, local neuromodulation. To do so, we focused on three predominant terminal fields of midbrain dopamine neurons, namely, the striatum (Str), amygdala (Amg), and prefrontal cortex (PFC; abbreviated here as Ctx), which together span dense and sparse innervation regimes and distinct dopamine neuronal subtypes (*46*–*48*). At the macroscopic level, conventional, nonexpanded immunohistochemistry against the AFLA-tag confirms that both D1 and D2 receptors are strongly enriched in the striatum (Fig. 7A), consistent with the high density of D1- and D2-expressing neurons in Str. However, this spatial scale does not address how receptor signaling machinery is spatially organized relative to dopamine release sites.

**Fig. 7.**
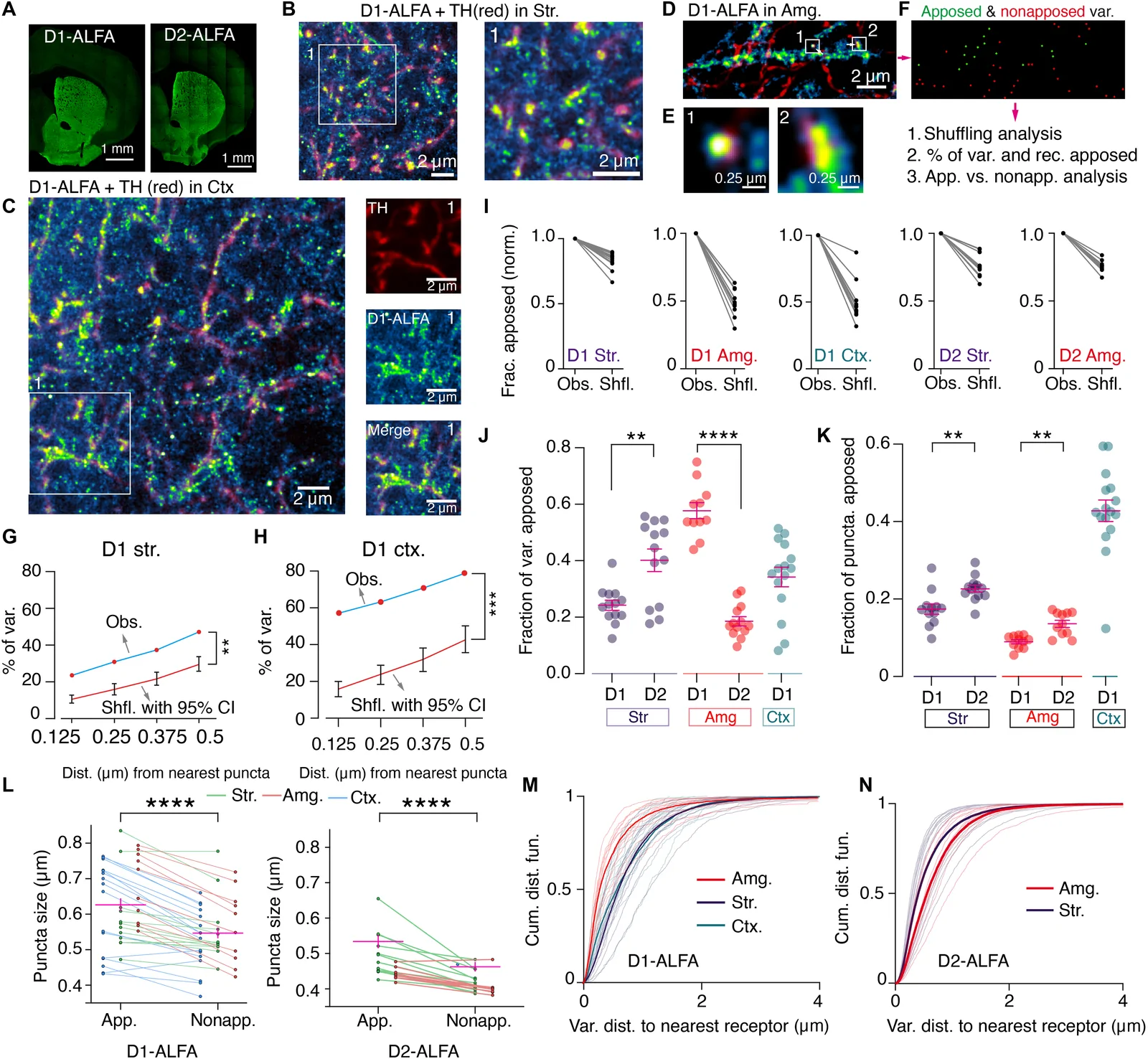
Visualizing the spatial organization of dopamine receptor microdomains in intact brain tissue using ExM. (**A**) Immunostained 50-μm coronal sections from ALFADoR mice, showing expected enrichment of D1 and D2 receptors in the striatum. (**B**) Two-color composite of D1-ALFA and dopamine axons (TH, red) in striatum, 4× expanded tissue. Right: Close-up of ROI 1 (white box). Scale bars represent real biological scale. (**C**) ExM images (4×) of D1-ALFA and TH^+^ axons in prefrontal cortex. Right: Close-up of ROI 1 (white box). (**D**) Expression in BLA (ExM, 4×); TH (red) marks DA varicosities. (**E**) Close-up of DA varicosities (red) in tight apposition with D1-ALFA clusters, corresponding to ROIs 1 and 2 in (D). (**F**) Varicosities are classified apposed or nonapposed via proximity analysis (see Materials and Methods); toroidal shuffling null models and nearest-distance analysis then quantify ExM volumes. (**G** and **H**) Toroidal shuffling of ExM data for D1-ALFA in striatum (str.) and prefrontal cortex (ctx.). Percentage of varicosities within 0.125, 0.25, 0.375, and 0.5 μm is evaluated from the original data (Obs., blue), then reevaluated after shuffling receptor puncta randomly and uniformly in the *xy* plane (*n* = 1000 runs), yielding mean and 95% CI (red). One-sided permutation test; **P = 0.02; ***P = 0.001. (**I**) Shuffling analysis as in (G) and (H) for three regions and both receptor subtypes, at the 0.125-μm appositional-contact point; each shuffled point (Shfl.) is normalized to observed data (Obs.). (**J** and **K**) Fraction of apposed varicosities and apposed puncta; means ± SEM are shown. Pairwise comparisons; two-sample KS test; **P < 10^−2^; ****P < 10^−4^. (**L**) Apposed (App.) versus nonapposed (Nonapp.) receptor puncta size differ significantly (KS test; ****P < 10^−4^; unpaired *t* test on pooled data). Magenta bars show means ± SEM of pooled data; points for the three regions are broken out to show regional differences, each a replicate’s mean puncta size. (**M** and **N**) CDFs of varicosity-to-nearest-receptor-puncta distance, three regions, and both receptor subtypes.

To analyze receptor organization at the scale relevant to putative release-receptor coupling implied by our earlier findings, we used ALFADoR mice and performed ExM in Str, Ctx, and Amg (*49*). In expanded tissue (expansion factor of ≈4), ALFA-tagged receptors appeared as discrete puncta rather than exhibiting diffuse membrane labeling, while DA axons visualized with a TH-stain (red) formed an arbor with typical varicosities (Fig. 7, B to D). In Str, the vast majority of TH-positive terminals are expected to be DA, whereas, in Ctx and Amg, TH can also label noradrenergic fibers. To address this potential confound, we validated TH labeling in Amg tissue containing genetically labeled DA axons and found that 65.9% of TH-positive varicosities were also tdTom^+^ (fig. S18), indicating that most of TH-positive varicosities analyzed in these fields are DA. Moreover, any residual contribution from non-DA catecholaminergic fibers would be expected to dilute, rather than inflate, estimates of dopamine varicosity coupling to D1/D2 receptor puncta. Qualitatively, these images frequently revealed dopamine varicosities positioned immediately adjacent to receptor puncta, including examples in the Str (Fig. 7C) and Amg (Fig. 7, D and E) where close apposition is particularly clear in magnified views. This suggested that the punctate receptor architecture and appositional coupling may provide a structural substrate for receptor microdomains that support locally constrained (i.e., synapse-like) dopamine signaling.

To quantify our ExM data, we implemented an analysis framework (schematized in Fig. 7F) that systematically extracts varicosities and receptor puncta from single or multiplane ExM images and quantified their spatial relationships. Briefly, TH axons were segmented and varicosities were identified using an ExM-optimized, watershed-based approach (Materials and Methods), and receptor puncta were segmented as discrete clusters, again using watershed-based algorithms. We then quantified spatial coupling by evaluating (i) minimum distance from each varicosity to the nearest receptor cluster, (ii) CDFs of varicosity-to-receptor, and receptor-to-varicosity distances, and (iii) analysis of receptor puncta stratified by whether they are classified as apposed versus nonapposed. Critically, we compared the observed spatial coupling against a conservative null model using toroidal shuffling in which receptor masks are shifted randomly in (*x*, *y*) with wraparound, preserving receptor number, size, and global density structure while disrupting their putative alignment relative to dopamine axons (see Materials and Methods for more). This provided anatomical region-specific “chance apposition” baseline without artificially redistributing puncta or changing their shapes.

Across all three regions, our data show that varicosity to receptor proximity cannot be explained by receptor density/chance-contact alone. In Str and Ctx (D1-ALFA), the fraction of varicosities within each of four increasingly permissive distance thresholds is substantially higher in the experimental data than in shuffled controls (Fig. 7, G and H), with highly significant differences (comparing mean of experimental data versus means ± 95% confidence interval (CI) evaluated from *n* = 1000 shuffling trials). A complementary “normalized” view (Fig. 7I) shows that, in all three anatomical regions, shuffling consistently reduces apparent coupling across all tested conditions (D1 Str/Amg/Ctx and D2 Str/Amg, D2 in Ctx was not included in our analysis because of low D2 expression), with particularly marked reductions for D1 coupling in Amg and Ctx, indicating strong spatially coupled organization in those regions. Specifically, shuffling reduced appositional contact to 49.8 ± 0.02% of that observed in nonshuffled data (Obs.) in D1-Amg and to 48.6 ± 0.03% in D1-Ctx, but only reduced it to 83.2 ± 0.02% in D1-Str (means ± SEM for all).

When we summarized coupling by receptor subtype and region (Fig. 7, J and K), we observed two complementary trends that help clarify the architecture of dopamine signaling microdomains. First, from a varicosity-centric perspective (Fig. 7J), coupling was observed to be both region and receptor-dependent: In Str, for example, D2-apposed varicosities exceed D1 (40.1 ± 4% versus 24 ± 1.8%, means ± SEM, *P* < 10^−2^), whereas, in Amg, the pattern reverses, with D1-apposed varicosities being notably higher than D2 (57.7 ± 2.8% versus 18.6 ± 1.6%, means ± SEM, *P* < 10^−4^). In Ctx, where only D1 expression was analyzed, we observed an intermediate level of coupling compared with that observed in Str. and Amg (34.2 ± 3.4%, means ± SEM). Second, from a receptor-centric perspective (Fig. 7K), the pattern differs in a way that is informative. Str and Amg show modest but significantly higher fractions of receptor puncta that are apposed for D2 relative to D1 (22.6 ± 0.9% versus 17.4 ± 1.3% in Str. and 13.6 ± 0.9% versus 8.9 ± 0.5% in Amg, means ± SEM), whereas, in Ctx, D1 exhibits a relatively high fraction of puncta apposed (42.8 ± 2.8%, means ± SEM). Considering the relatively low percentage of receptor puncta that are appositionally engaged (Fig. 7K), we asked whether apposed receptor clusters differ structurally from nonapposed clusters. Across all three regions, receptor puncta classified as apposed were systematically larger than nonapposed puncta, for both D1-ALFA and D2-ALFA (*P* < 10^−4^) (Fig. 7L). This result suggested that apposition is not simply a geometric coincidence but is associated with distinct receptor microdomain properties and is consistent with the idea that apposed clusters may represent functionally specialized sites with enhanced receptor content that elicit local signaling.

Last, we performed varicosity-to-receptor and receptor-to-varicosity distance distribution analysis. CDF analyses of varicosity-to-nearest-receptor distance (Fig. 7M) showed clear regional structure: For D1-ALFA, Amg distances are most left-shifted (closest coupling) relative to Str and Ctx, consistent with a gradient of nanoscale receptor proximity across all three terminal fields. For D2-ALFA, Str exhibits a notably left-shifted distribution relative to Amg, suggesting stronger D2 coupling in striatum (Fig. 7N). The presence of distance distributions that vary based on receptor type and anatomical region, combined with shuffle sensitivity, supports the premise that dopamine receptors are not merely stochastically distributed in axonal terminal fields but are organized into spatially biased nanoclusters relative to dopamine varicosities.

Together, these findings provide convergent evidence that endogenous dopamine receptors form punctate nanoclusters that are nonrandomly positioned relative to dopamine varicosities across major DA axonal terminal fields. The combination of strong shuffle sensitivity, region- and receptor-specific coupling patterns, puncta size differences at apposed versus nonapposed receptors, and distance-CDF structure supports a model in which dopamine neuromodulation engages in synapse-like, spatially restricted microdomains, with the balance between targeted appositional signaling and broader extrasynaptic modulation varying by anatomical context and receptor subtype.

## DISCUSSION

A prevailing framework for neuromodulatory communication is volume transmission, in which signaling molecules released from axons diffuse through extracellular space to engage receptors at variable distances while competing with uptake, enzymatic degradation, and bulk clearance. This model captures the fact that neuromodulators can influence many targets over extended spatial scales. However, the model also leaves unresolved how such a comparatively imprecise, one-to-many mode of communication supports the highly specific computations attributed to dopamine circuits (*20*, *21*). In particular, key mechanistic questions remain unanswered, namely, (i) how neuromodulatory axons identify and stabilize within their terminal fields, (ii) whether they preferentially engage particular cellular targets in their terminal fields, and (iii) what spatiotemporal constraints govern receptor activation in densely innervated neuropil where receptors are broadly distributed. Even with a mature understanding of GPCR signaling cascades, the rules by which neuromodulatory axons find “where to signal” and how release events are translated into organized receptor engagement remain incompletely defined.

In this study, we converge on a model in which dopamine transmission frequently exhibits synapse-like organization, providing an anchoring principle for spatial precision that may be broadly applicable to monoaminergic neuromodulatory signaling. This model was motivated initially by functional observations that are difficult to reconcile with purely stochastic diffusion. In acute slices and cultured preparations, dopamine release is not spatially uniform but instead forms reproducible hotspots, suggesting that dopamine output is organized into discrete release-competent sites rather than being a stochastic property of all varicosities. Extending this to a mechanistic test, single-bouton optical recordings in cocultures indicated that release competence is tightly linked to appositional coupling: Dopamine boutons become release competent specifically when they contact dopamine receptor–positive soma and dendrites, implying that the local postsynaptic environment is not merely a recipient of diffusing transmitter but participates in specifying where release occurs. These functional data helped us establish a key premise that dopamine signaling can be spatially and structurally constrained and motivated a search for the anatomical substrates that support such constraints.

Our anatomical and molecular analyses in intact tissue provided convergent evidence for those substrates. Using EASI-FISH in amygdala and the striatum and in coculture, we observed that dopamine axons exhibit pronounced tropism for receptor-expressing processes, consistent with a model in which neuromodulatory projections may be guided by molecular features of their targets. This addresses a long-standing conceptual gap in volume transmission models: If synaptic coupling is dispensable for release of neuromodulators, it is unclear what rules govern how neuromodulatory axons navigate to and stabilize within appropriate terminal fields, often millimeters away from their midbrain origins. Tropism for receptor-positive cellular microenvironments provides a plausible mechanism for both targeting and local stabilization, and it predicts that synapse-like, contact-dependent mechanisms could influence bouton maturation and release competency.

Consistent with these observations, the ExM analyses presented here provide nanoscale resolution of how dopamine axons and receptors are arranged once axons reach their targets. Across striatum, amygdala, and prefrontal cortex, endogenous D1 and D2 receptors are organized into discrete nanoclusters and are preferentially positioned near dopamine varicosities at distances significantly smaller than expected from receptor density/chance encounter alone. Conservative shuffle controls that preserve receptor geometry and spatial heterogeneity while disrupting alignment markedly reduce apparent coupling, indicating that the observed proximity reflects structured organization rather than random overlap. Moreover, receptor puncta that are apposed to dopamine varicosities are larger than nonapposed puncta, suggesting that apposition is associated with a clustering similar to those observed in classical synapses. Together, these results place subcellular receptor clustering and varicosity apposition at the center of a synapse-like model in which local microdomain structure helps define where dopamine signaling is most efficient and potentially most functionally consequential. One possible mechanism by which this happens is dopamine receptor–enriched somatodendritic microdomains act as molecular hubs that locally concentrate adhesion molecules, scaffolding proteins, or extracellular matrix components, thereby stabilizing apposed DA boutons and promoting recruitment of presynaptic release machinery. This potential mechanism is consistent with prior studies implicating transsynaptic cell-adhesion molecules, including neurexin/neuroligin family proteins, in the formation and maintenance of DA contacts (*17*, *19*, *50*). Future work combining unbiased proteomics with targeted perturbations will be needed to establish a causal link between synapse-like appositions with presynaptic release maturation.

The synapse-like model also helps reconcile why only a subset of dopamine varicosities appear release competent, an observation that is consistent with previous literature (*51*). Under a purely volumetric model, heterogeneity is often treated as stochastic sampling within dense arbors or as an intrinsic presynaptic property. In contrast, our combined functional and anatomical results support a scenario in which contact-dependent release-maturation is a key determinant of functional release sites. In this model, appositional coupling may serve as the anatomical “selection” step that stabilizes and concentrates release machinery at particular varicosities while leaving others comparatively silent. Consistent with this, we find that presynaptic components classically associated with rapid neurotransmitter release are enriched at dopamine-releasing varicosities [Bsn, Rim (*26*), Stx1, and others] and that ultrastructural analysis by TEM reveals synapse-like contact at a subset of dopamine boutons. The ultrastructure is not simply a copy of canonical fast synapses; instead, it exhibits nuanced differences consistent with a specialized form of coupling optimized for neuromodulatory signaling. These molecular and ultrastructural data provide a mechanistic bridge between bouton-level release hotspots and the receptor-varicosity architecture revealed by ExM.

Last, our findings do not argue that diffusion is irrelevant per se; rather, they suggest that stochastic release and diffusion is unlikely to be the primary organizing principle for dopamine signaling. Dopamine released at synapse-like microdomains may still spill over to influence peri-synaptic or extrasynaptic receptors, particularly under high activity or in fields with reduced clearance. However, anchoring release to receptor-proximal sites provides an efficient strategy for achieving the high local concentrations and rapid kinetics needed to reliably engage GPCR pathways under physiological constraints. This is consistent with prior slice-based observations that certain forms of dopamine-dependent GPCR responses, including D2-mediated GIRK currents, are preferentially driven by rapidly rising, high-amplitude dopamine transients rather than by slow, low-level tone (*52*, *53*). In our model, synapse-like release-receptor appositions represent a fundamental unit of neuromodulatory organization that create spatial precision and biochemical efficiency while still permitting broader modulation through spillover and network-level integration. Thus, we propose that dopamine transmission is best understood as a hybrid system in which synapse-like microdomains provide the core substrate for precise signaling, embedded within a context where diffusion can extend influence beyond the immediate site subject to activity vigor and local molecular landscape.

## MATERIALS AND METHODS

All chemical reagents and antibodies are listed in tables S1 to S3. Synthesis of the nanosensors (nIRCat) and fabrication of DopaFilm have been published previously (*28*, *29*). Plasmid for hSyn-G-Flamp1 (Addgene, no. 188569) was purchased from Addgene, and pAAV–EF1a-DIO-APEX2.NES construct was subcloned into a lenti backbone by VectorBuilder. Lentiviruses containing shRNA KD sequences were sourced from VectorBuilder. Virus packaging was performed at the Janelia Research Campus of the Howard Hughes Medical Institute (HHMI). All data analysis was performed using MATLAB_R2024a unless otherwise noted.

### Ethical considerations and mouse strains

All experiments were conducted according to the Institutional Animal Care and Use Committee guidelines of Janelia Research Campus of the HHMI under protocol number 25-0287.03. For culture and tissue experiments, we used *Slc6a3*-Cre x Ai9 (*54*, *55*), ALFADoR1 mice (this work), ALFADoR2 mice (this work) transgenic P1 to P2 pups and adult (P50 to P60) mice.

### ALFADoR1/2 KI mouse generation and validation

Both transgenic mouse lines were generated by CRISPR-cas9 pronuclear microinjection by the Transgenic Facility at Janelia Research Campus of the HHMI. Male mice older than 8 weeks were mated with 24- to 27-day-old superovulated C57Bl/6J females (from the Jackson Laboratory) to obtain zygotes for pronuclear injection. CD-1 Elite females (Charles River Laboratories) were used as embryo transfer recipients. Pronuclear-stage embryos were collected in M2 (Tribioscience, TBS8070; 50 ml), washed three times in KSOM (Tribioscience, TBS8071; 50 ml) to remove cumulus cells, and incubated in KSOM at 37°C and 5% CO_2_. The mix of Cas9 ribonucleoprotein and donor double-stranded DNA was injected into the pronuclei of the zygotes. Following injection, embryos were incubated in KSOM until all injections were complete. Approximately 10 to 15 embryos were transferred into the oviducts of pseudopregnant CD-1 Elite recipient females on the same day. Cas9 protein (TRUECUT CAS9 PROTEIN V2, 100UG) was purchased from Life Tech Corp (A36498). The single guide RNA (sgRNA; 5′-GTTAGGAGCCATCTTCCAGAAGG-3′) was generated by in vitro transcription using MEGAshortscript T7 kit (LIFE TECH CORP, AM1354) and purified with MEGAclear Transcription Clean-Up Kit (LIFE TECH CORP, AM1908). The following donor DNA [500–base pair (bp) double-stranded fragment] was synthesized by Integrated DNA Technologies.

*Drd1* ALFA-tag donor sequence: GGTGGAAAGCAAGCGGCTCTTCTTCCTGGTATGGCTTGGATTGCTATGGAGATGCTCCTGATGGAACACCATTGTGCTTTTGTCCAGACAGCAACTGGGGCTGGAGAAGGGGCTGGGTGGTGAGTGATTGGGGGAAGTCTGGCTAAGCCTGGCCAAGAACGTGAGGGCTAAGCCACCGGAAGTGCTTTCCTTCTGGAAGGCCACCATGccgagccgcctggaagaagaactgcgccgccgcctgaccgaaccgGCTCCTAACACTTCTACCATGGATGAGACTGGCCTGCCAGTGGAGAGGGACTTCTCCTTTCGCATCCTCACAGCCTGTTTTCTGTCCCTGCTTATCCTGTCCACTCTCTTAGGGAATACCCTTGTCTGTGCCGCTGTCATCAGGTTTCGACACCTGCGGTCCAAGGTGACCAACTTCTTTGTCATCTCTTTAGCTGTGTCAGATCTCTTGGTGGCTGTCTTGGTCATGCCCTGGAAAGCTGTGGCTG

*Drd2* ALFA-tag donor sequence: GCTAGAAGAACAGAAGCTTGTCCTAACATTATAAGATCGCTAGAACCAGGAATAAAAAAAACATAGTTTGGGGAATTCTCAGCTCTGCTAGCTCTTGGTTTTTCTGCAGGGAATCCTCTTTAGGAGGAAGCATGCCTTGAAAACACTCCTGCTCACTCCTTGTATTCATTTCTCCCGGCCAGAGCCGTGCCACCCAGTGGCCCCACTGCCCCAGCCACCATGccgagccgcctggaagaagaactgcgccgccgcctgaccgaaccgGATCCACTGAACCTGTCCTGGTACGATGATGATCTGGAGAGGCAGAACTGGAGCCGGCCCTTCAATGGGTCCGAAGGGAAGCCAGACAGGCCCCACTACAACTACTATGCCATGCTGCTCACCCTCCTCATCTTTATCATCGTCTTTGGCAATGTGCTGGTGTGCATGGCTGTATCCAGAGAGAAGGCTTTGCAGACCACCACCAACTACCTGATAGTCAGCCTCGCTGTGGC

The ALFA tag sequence (PSRLEEELRRRLTEP) was inserted into the second exon (E2) of either *Drd1* or *Drd2* genes after the start codon, resulting in a fusion with the endogenous gene. For *Drd1* and *Drd2*, the inserts were flanked by left and right homology arms of 199 bp/247 bp and 213 bp/233 bp, respectively. For microinjections, the Cas9 protein/sgRNA/double-stranded DNA donor mix was suspended in 0.22-μm-filtered 10 mM tris-Cl buffer (pH 7.5) at 30 ng/30 ng/8 ng for Drd1 and 5 ng for Drd2, respectively. F_0_ mice were genotyped using PCR with primers as listed in table S4.

Targeted insertion was further confirmed by Sanger sequencing using the following primers:

*Drd1* 5F: 5′-TGGAGCACTGAACCCAGAAG-3′, and 3R: 5′-CGTGGAGCACATGATGTCAA-3′ (WT, 543 bp; and mut, 594 bp).

*Drd2* 5F: 5′-GAAAGAATGGATGAGTTAGCTC-3′, and 3R: 5′- AGTGTGGCCACCAGAAGAT C-3′ (WT, 549 bp; and mut, 600 bp).

### Preparation of primary mouse neuronal cultures

Primary mouse neuronal culture work was carried out as previously published (*28*) with some modifications. Briefly, *Slc6a3-cre* x Ai9 transgenic neonatal pups (P1 to P2) were euthanized, and tissue sections from the cortex, striatum, and SNc and ventral tegmental area (VTA) from midbrain region were dissected. We used a fluorescence-guided dissection microscope (Zeiss SteREO Discovery.V20) to identify and maximize the extraction of tdTom-expressing cells in SNC and VTA regions. Mixed cocultures were generated by extracting dopamine neurons in the SNC + VTA regions from *Slc6a3*-cre x Ai9 transgenic mice and cortical or striatal regions from ALFADoR1/2 mice. SNC + VTA cells were seeded as cocultures with either cortical cells or striatal cells at a ratio of 3:7, and density of 30,000 cells per 35-mm MatTek dish (14-mm insert), in attachment medium (1:1 v/v plating medium to NbActiv4) on top of DopaFilm substrates. Cultures were initially maintained in attachment medium for 3 hours in a 5% CO_2_ humid incubator at 37°C. Next, attachment medium was replaced with growing medium (plating medium: NbActiv4, 1:20 v/v), and cells were allowed to grow for 1 week. After 1 week, NbActiv medium (500 μl) was added on top of growing medium, and cultures were fed twice a week. Viral transductions with hSyn-G-Flamp1 (AAV2), pAAV–EF1a-DIO-APEX2.NES, and KD constructs (lentis) for presynaptic proteins were carried out around 5 to 7 days in vitro (DIV 5 to 7). shRNA KD constructs were designed using the Genetic Perturbation Platform (Broad Institute). We chose the top three-scoring KD constructs per gene, on the basis of KD scores reported on the web portal. Each gene perturbation experiment used equal titers of the three constructs simultaneously.

### Data acquisition and analysis for neuronal activity imaging

We carried out activity imaging in neuronal cocultures (DIV 14 to 21) using a custom-built broad spectrum (400 to 1400 nm) epifluorescence microscope with a 25× water immersion objective (Olympus) and two cameras [SWIR/NIR on NiNox 640 II, Raptor photonics, and visible on Hamamatsu ORCA FusionBT (C15440)] built on a Thorlabs Bergamo microscope body. Before activity imaging experiments, we aspirated the NbActiv4 (growth medium) and washed cells 2× with fresh artificial cerebrospinal fluid (ACSF) and incubated them with 5-part-per-million nanosensors in ACSF for 1 hour inside an incubator (5% CO_2_, 37°C). The ACSF composition was as follows: 124 mM NaCl, 2.5 mM KCl, 1.25 mM NaH_2_PO_4_, 24 mM NaHCO_3_, 12.5 mM glucose, 5 mM Hepes, 2 mM CaCl_2_, and 2 mM MgSO_4_. We then washed cells 2× and mounted the dish on the microscope stage for imaging. Before imaging sessions, we first saved the tdTom and bright-field images and switched to the NIR channel for DopaFilm activity measurements. Green fluorescent protein–based sensors and tdTom-positive axons were excited using a light-emitting diode light source (Thorlabs, LED4D067), and DopaFilm/nIRCats were excited using a 785-nm laser (Thorlabs, S4FC785) with 100-mW power at the source, giving ∼30 mW at the sample. For dopamine activity imaging experiments, 500 images were acquired with μManager (*56*), at ∼10 frames per second at room temperature (RT). To reliably evoke dopamine release, field stimulation (five pulses at 25 Hz, 1-ms pulse width) was applied to cultured neurons using custom-made platinum electrodes connected to a stimulus isolator (A385, World Precision Instruments) after 15 s of baseline activity were collected.

We performed analysis of dopamine imaging data as previously reported using a custom-built MATLAB code (*28*), available at: https://github.com/JaneliaSciComp/nnmf/commits/main/. For analysis, raw image files were first convolved with a 2D Gaussian (σ = 0.5 pixels). To obtain baseline intensity values at each pixel (i.e., *F*_0_), we used a leaky cumulative minimum and applied repeated low pass filtering to converge on a smooth *F*_0_. We then computed ∆*F* as *F* − *F*_0_. Next, we applied NNMF to Δ*F* with sparsity and contiguity constraints to partition the image into correlated components. NNMF is a dimensionality reduction method that segments the image into correlated regions (independent components) that best approximate the underlying image. This effectively groups pixels with similar activity into components, and these components spatially colocalize with the fluorescence hotspots detected on DopaFilm. NNMF masks were then used to compute ∆*F*/*F* traces corresponding to each hotspot. For clustering analysis related to Fig. 2 data (and presented as a summary in Fig. 2, H to J), we first binarize the tdTom channel and classify active and quiescent varicosities based on their overlap with ∆*F* channel. We then compute the pairwise distances between tdTom pixels at ∆*F* hotspots (active), nonhotspots (quiescent), or over all tdTom pixels (i.e., all boutons). Proximity analysis for an individual region was performed by taking the distance transform of a binarized MAP2 image. The distance from MAP2 as a function of normalized Δ*F* is plotted, with error bars showing 95% CI.

### Proximity and OR analysis in mixed-culture datasets

We used three coregistered 2D TIFF images (single field per analysis), including (i) tdTom labeling dopamine axons, (ii) D1 or D2 dopamine receptor immunofluorescence, and (iii) Δ*F*/*F* images reporting dopamine release. Images were converted to grayscale and intensity normalized. Background was reduced with a morphological top-hat filter. Axons were segmented from the tdTom channel using adaptive thresholding, followed by small-object removal and hole filling. To minimize bias from axon thickness, we skeletonized the axon mask. Receptor puncta were detected from a Laplacian-of-Gaussian response image (σ ≈ 0.8 to 2.0 pixels) thresholded on *z*-scored intensity (typically, *z* > 2 to 4), constrained by local maxima, and then cleaned by area filtering (to remove small and large objects). For robustness to the point-spread function and minor misregistration, we optionally dilated the receptor mask by 2 pixels before distance computation. Release regions (Δ*F*/*F*) were segmented by adaptive thresholding minimum area filtering (remove large and small objects).

We computed a Euclidean distance map to the nearest receptor pixel. Distances were sampled over axon pixels and partitioned into release^+^ (axon ∩ Δ*F*/*F*, active) and release^−^ (axon ∩ not-Δ*F*/*F*, quiescent). We summarized the distributions with empirical CDFs. For OR calculations, for radii *r* spanning 0.3 to 2.0 μm, we defined proximity as distance ≤ *r*. For each *r*, we tabulated axon pixels within versus outside *r* for release^+^ and release^−^ groups and computed the OR as fraction (or percentage) of active varicosities that are at ≤*r* divided by fraction (or percentage) of quiescent varicosities that are at ≤*r*. OR values close to 1 indicate no difference in enrichment. We estimated 95% CIs for OR by bootstrapping log(OR) with resampling within each group (4000 iterations) and exponentiating the 2.5th to 97.5th percentiles. We visualized OR versus *r* with corresponding bootstrap CIs; values above 1 indicate enrichment of D1R proximity among release^+^ axon pixels.

### Acute slice imaging

Acute coronal brain slices containing DMS or amygdala were prepared as described previously, and extracellular dopamine dynamics was recorded using nIRCats (*29*). Briefly, acute slices were labeled with dopamine nanosensors in solution phase via passive incubation at RT (15 min) in ACSF containing the nanosensors (2 μg/ml). Slices were allowed to equilibrate in a submerged recording chamber under continuous perfusion of oxygenated ACSF (95% O_2_/5% CO_2_) and transferred to an imaging chamber under perfusion for activity recording. Dopamine release was monitored optically by imaging dopamine-evoked fluorescence transients in the FOV during local electrical stimulation. A bipolar stimulating electrode was positioned on the slice surface within the imaging region under low magnification (×4). Then, a high-magnification objective (25×, Olympus) was used to select a FOV at a distance of ∼100 μm from the electrode for activity recording. Time-lapse imaging was acquired at 10 frames per second, with baseline collected for 15 s before stimulation; each acquisition lasted 500 frames. Single-pulse electrical stimuli (0.3 mA, 1 ms) were delivered to evoke dopamine release using WPI A365 Stimulus Isolator.

### Post hoc immunocytochemistry, confocal, and Airyscan imaging

Following activity recording, the imaging buffer (ACSF) was aspirated, and the specimens were fixed with 4% PFA at RT for 10 min. Fixative was then aspirated, and cells were washed 3× with DPBS. After blocking with blocking buffer [2% bovine serum albumin (BSA) in 0.1% Triton X-100 in phosphate-buffered saline (PBS)] for 1 hour at RT, tissues were incubated with primary antibodies diluted in antibody dilution buffer (1% BSA in 0.1% Triton X-100 in PBS) overnight at 4°C. The samples were then rinsed multiple times with PBS and incubated with secondary antibodies diluted in antibody dilution buffer for 3 hours at RT. To probe the ALFA-tag in fixed cultured cells, we performed an extra heat induced epitope retrieval step using 5% urea in 100 mM tris buffer (pH 9.5) for 10 min at 80°C. Standard confocal and Airyscan images were acquired on a Zeiss LSM980 inverted microscope equipped with a Plan-Apochromat 40× or a 63× (1.2 NA water or 1.4 NA oil, respectively) objective.

### Immunohistochemistry on brain slices

Transgenic adult mice were anesthetized with isoflurane and perfused transcardially, sequentially with cold PBS and 4% PFA (50 ml each). After perfusions, isolated brains were sectioned using a vibratome (Leica VT1000 S) to get 50-μm-thick coronal brain slices. After blocking with blocking buffer for 1 hour at RT, tissues were incubated with primary antibodies (rabbit anti-TH, 1:500; and human anti-ALFA, 1:250) diluted in antibody dilution buffer overnight at 4°C. The samples were then rinsed multiple times with PBS and incubated with secondary antibodies diluted in antibody dilution buffer for 3 hours at RT. Tissue sections were mounted onto glass slides (Fisher Scientific, catalog no. 12-550-15), and #1.5 glass coverslips (EMS, catalog no. 63793-01) were applied using VECTASHIELD mounting medium (Vector Laboratories, part no. H-1400-10). Confocal imaging was carried out using a spinning disk confocal microscope [Nikon CFI Plan Apo Lamda 60× oil/1.4 numerical aperture (NA)]. Images were denoised, deconvoluted, and stitched using the in-built NIS-Elements software.

### Data acquisition and analysis of EASI-FISH volume

Experimental procedure for EASI-FISH experiments was adapted from a previously published protocol (*36*). Briefly, *Slc6a3* x Ai9 mice were anesthetized with isoflurane and perfused transcardially with 15 ml of ribonuclease-free PBS followed by 50 ml of ice-cold 4% PFA. After overnight postfixation in the same fixative, brains were sectioned coronally into 300-μm slices. Slices containing amygdala or the striatum were selected for the study. We used TREx-1000 gel for the study, and the gel recipe is published elsewhere (*57*). Hybridization probes for *Drd1* and *Drd2* were used at a concentration of 1 μM and amplified using hairpins conjugated to AF488 and JF669 at a final concentration of 3 μM. The endogenous tdTom signal (i.e., dopamine axons) was amplified by IHC using the anti–red fluorescent protein (RFP) primary antibody (1:500) followed by an anti-rabbit secondary antibody conjugated AF594 (1:200). The gels were imaged with a Zeiss Light Sheet 7 microscope.

EASI-FISH image analysis was performed in 3D using a cell-indexed workflow that enabled cellwise extraction of receptor signals and dopamine axon-associated fiber/bouton features. Nuclei were segmented from the DAPI channel using Cellpose to generate an indexed 3D label mask. To approximate cell-associated volumes around each nucleus, the indexed mask was then expanded by 8 μm in 3D using an anisotropic, label-preserving distance-transform dilation method. During dilation, background voxels within the dilation radius were assigned the label of the nearest nucleus, preventing higher-index labels from overwriting lower-index labels during expansion.

Using the resulting expanded cell index ID mask, the *Drd1*, *Drd2*, and axon channels were clipped in 3D on a per-cell basis. For each cell ID, maximum-intensity projections (MIPs) were generated at an image size of 180 × 180 pixels. Two MIP versions were produced: a two-channel MIP containing only *Drd1* and *Drd2* signals, and a three-channel MIP containing *Drd1*, *Drd2*, and the axon channel. Only the two-channel MIP version was used for machine learning training, and the trained classifier was applied for four-class separation.

To train the classifier, we manually selected 25 cell-ID MIPs for each of four classes: *Drd1*-only positive, *Drd2*-only positive, *Drd1* and *Drd2* both positive, and *Drd1* and *Drd2* both negative. For machine learning classification of these two-channel MIP datasets into the four experimental classes, we used a lightweight convolutional neural network (CNN) implemented in PyTorch. Each input image was loaded in RGB (not including the axon channel), converted to a tensor, and intensity normalized. The CNN (“SmallCNN”) consisted of three convolutional blocks with 3 × 3 kernels (padding of 1) and increasing channel depth (16 → 32 → 64). Each convolution was followed by a ReLU nonlinearity and 2 × 2 max pooling. Features were flattened and passed through a fully connected layer (128 units, ReLU) followed by a final linear layer producing four output logits corresponding to the classes: *Drd1*, *Drd2*, Both_Negative, and Both_Positive. For inference, model weights were loaded from a saved PyTorch state dictionary (.pth), and classification was performed in evaluation mode with gradient computation disabled. For each image, class probabilities were computed using a softmax operation over the four logits, and the predicted class was assigned as the argmax of the probability vector. The associated confidence score was defined as the maximum softmax probability for the predicted class. Model performance was evaluated using an 80/20 stratified train/validation split. On the held-out validation set (*n* = 20), the model achieved 95% accuracy (macro-average precision of 0.96, recall of 0.95, F1 of 0.95). Classwise F1-scores were: both negative, 1.00; both positive, 0.89; *Drd1*, 0.91; and *Drd2*, 1.00. To facilitate downstream analysis and manual review, images were automatically organized into class-specific output folders, and each file was renamed to embed the prediction confidence and predicted class.

Axon fiber and bouton analyses were then performed within each cell ID’s 3D index mask (using the 8-μm dilated mask). Entire axon fiber light-microscopy data were segmented in 3D using a direction-selective local-thresholding method. The segmented fiber volume was clipped by the 8-μm dilated cell index mask to assign fiber structures to individual cells. For axon fiber quantification, 3D skeletons were created, and short terminal spurs were pruned while preserving the main trunk; these short terminal spurs were treated as artificial branches arising from reduced *z* optical resolution. Fiber length was measured on the basis of skeleton length. For branch point counting, we counted three-branch intermediate points on the 3D skeleton.

Bouton structures were quantified from the axon channel within each cell-ID mask as follows: A MIP of the axon stack was generated, and an automatic intensity threshold was estimated on the MIP using ImageJ (“Default dark,” 16-bit). The resulting lower threshold was tightened for striatum data (final cutoff of 1.4× lower) and applied to the full 3D synapse volume to generate a binary bouton candidate mask. The mask was denoised using outlier removal and converted into an indexed 3D object map by connected-component labeling (minimum size of 3 voxels), yielding bouton candidate volumes.

To estimate fiber geometry, a 3D Euclidean distance transform (EDT) was computed, and a diameter threshold was set to 0.1874 μm for amygdala and 0.002 μm in striatum. The bouton mask was skeletonized in 3D; skeleton endpoints and branch points were annotated, and short terminal spurs were pruned (truncation of 1). Bouton objects were further classified in amygdala using a skeleton-thickness profile matching method. Specifically, a 3D EDT volume was used as a local radius/thickness estimate, and skeleton endpoints and branch points were identified in the skeleton mask. From each endpoint, the algorithm traced the skeleton voxel by voxel in 3D (26-neighborhood), propagating until reaching a branch point, while recording along the traced path: (i) the EDT-derived thickness values and (ii) the corresponding connected-component bouton index value at each voxel (with a 3 × 3 × 3 neighborhood mode fallback when the center voxel index was 0). For each bouton candidate (connected-component ID), the collected thickness sequence was summarized into a compact “shape” by averaging thickness over three equal-length bins, generating a low-dimensional thickness-profile vector. This vector was compared to predefined reference templates representing bouton-like versus neurite-like profiles using cosine similarity. Final bouton calls were made by combining the average thickness and the profile similarity scores (bouton templates versus neuron template).

In the striatum, a 3D EDT was computed to estimate local thickness, and per-object thickness statistics (mean and maximum diameter) were aggregated for each connected-component ID. In preliminary filtering, objects with mean diameter ≈ maximum diameter were treated as trunk-like structures (uniform thickness, consistent with neurite shafts) and were removed first. However, in the striatum, many true bouton-like structures were very thin and could still exhibit near-uniform thickness, causing them to be mistakenly classified as trunk-like by diameter-based rules. Because diameter-based thresholding could not reliably separate striatal boutons from thin nonbouton structures without discarding valid boutons, we did not apply any diameter/thickness thresholding in striatum and instead retained all candidates that passed the upstream intensity thresholding. Objects classified as boutons were retained in a filtered bouton-only index volume for downstream quantification of bouton number, volume, and skeleton length. For each cell ID, we reported bouton number, axon volume (scaled by voxel volume), and axon skeleton length (scaled by pixel size).

### Modified RNAscope protocol for cultured neurons

RNA transcripts for *Drd1* and *Drd2* genes were probed according to the manufacturer’s recommended protocol with some modifications. These modifications include (i) milder target retrieval step (80°C for 5 min instead of 100°C for 15 min) and (ii) reduced time for Protease III digestion (5 min instead of 30 min). During the RNAscope protocol, tdTom signal becomes heavily attenuated, and we included a post-ICC step with rabbit anti-RFP antibody (1:1000) and amplified using an anti-rabbit secondary antibody conjugated to AF594 (1:2000).

### LC-MS/MS–based protein quantification and data analysis

To assess the KD efficiency of presynaptic proteins, cortical neurons were cultured in six-well plates at a density of 1 × 10^6^ cells per well. Following shRNA-mediated gene silencing using lentiviral transduction at DIV 5 to 6 (three wells per protein target), cells were lysed at DIV 15 to 16 in ice-cold 1× radioimmunoprecipitation assay (RIPA) buffer supplemented with protease inhibitor (200 μl per three wells). Cells were detached using a cell scraper and transferred into prechilled 2-ml Eppendorf tubes, gently vortexed, and incubated on ice for 20 min. Lysates were centrifuged at 13,000*g* for 20 min at 4°C, and the resulting supernatants were collected and stored at −20°C. The whole-cell lysates (supernatants) from each strain were precipitated with cold acetone at 1:4 (v/v) ratio, −20°C for overnight. Protein pellets were centrifuged and washed with cold acetone. Residue acetone was then air dried, and the proteins were redissolved using 0.25% RapiGest according to the manufacturer’s protocol. Afterward, proteins were reduced with 5 mM dithiothreitol (65°C, 30 min) and alkylated using 11 mM iodoacetamide (IAA) at ambient temperature in dark for 30 min. Trypsin was added at a protein-enzyme ratio of 50:1 for overnight digestion at 37°C. The digestion was quenched by adding 10% trifluoroacetic acid to pH between 1 and 2 and further incubated at 37°C for 45 min. The solutions were then centrifuged at 14,000 rpm for 10 min, and the supernatant peptides were collected. Desalting of the peptides was performed using C18 ZipTip (Millipore, ZTC18M096), and the eluents were dried using SpeedVac (Thermo Fisher Scientific). The samples were stored at −80°C before being resuspended in 0.1% formic acid for LC-MS/MS analysis.

To quantify dopamine receptor levels in WT, D1-ALFA, and D2-ALFA mice, animals (P60 to P90) were deeply anesthetized with isoflurane and decapitated, and the brains were rapidly extracted into ice-cold ACSF. The striatum was quickly dissected, placed into a 1.5-ml protein low-bind tube, and immediately snap-frozen in liquid nitrogen. Each frozen striatal sample was cut into small pieces and homogenized in 1 ml of high-SDS RIPA buffer [50 mM tris-HCl (pH 7.4), 150 mM NaCl, 0.25% deoxycholic acid, 1% NP-40, 1 mM EDTA, 1% SDS, and 1× Halt Protease Inhibitor Cocktail in water] using 1.5-mm Benchmark TriplePure Zirconium Beads (D1032-15). The SDS concentration was then diluted to 0.2% with SDS-free RIPA buffer, and samples were probe-tip sonicated for 15 min at 30% power. Whole-protein lysates were isolated by ultracentrifugation at 100,000*g* for 1 hour at 4°C. The supernatant was carefully collected, and protein concentration was determined using the Qubit BR assay. Lysates were then cleaned up using Sera-MagSpeedBeads, with proteins aggregated onto the beads and digested on-bead.

LC separation was performed on a Vanquish Neo System (Thermo Fisher Scientific) with an IonOptik Aurora Ultimate C18 column (15-cm length by 75-μm inner diameter by 1.7-μm particle size) at 35°C and a flow rate of 300 nl/min. Mobile phase A consisted of 0.1% formic acid in water. Mobile phase B consisted of 0.1% formic acid in 80% ACN. Eluting peptides were ionized by electrospray ionization and then analyzed by an Orbitrap Ascend Tribrid mass spectrometer (tune version 4.2.4321, Thermo Fisher Scientific). Ion transfer tube temperature was set to 275°C. Source positive ion voltage was set to 1950 V. For single-shot proteomics with data-independent acquisition (DIA), the MS1 scan resolution was set to 120,000 [at 200 mass/charge ratio (*m*/*z*)]. MS1 scan range was 400 to 980 *m*/*z*, AGC target was 225%, and maximum injection time mode was set to auto. Precursors were isolated with an isolation width of 12 *m*/*z* covering 145 to 1450 *m*/*z*. Precursors were fragmented by HCD at an NCE of 26%. MS2 scans were acquired by Orbitrap with a resolution of 15,000. Maximum injection time was 59 ms for the Ascend.

For acquired DIA data, raw data were directly processed using DIA-NN (1.9.2) with *Mus musculus* (Mouse, UP000000589) proteome fasta file. A maximum of one missed cleavage was allowed and cysteine carbamidomethylation (+57.0215 Da) as the fixed modification. Oxidation (M) and acetylation (N-term) were selected as variable modifications. In addition, precursor FDR was set to 1%. The mass accuracy and MS1 accuracy tolerance window were optimized by DIA-NN during the processing. Match-Between-Runs was selected, and the quantification matrix was generated after processing. All statistical analyses were performed using R (version 4.3) with the following packages: tidyverse for data manipulation; readxl and writexl for Excel file handling; limma for differential expression analysis; pheatmap and ComplexHeatmap for heatmap visualization; ggplot2 with ggrepel for publication-quality plots; clusterProfiler and enrichplot for GO enrichment; fgsea and msigdbr for GSEA; UpSetR for set intersection visualization; and dendextend for dendrogram customization. Differential expression magnitudes were visualized using log_2_ fold change metrics relative to WT controls. All analysis scripts are provided as the Supplementary Materials.

Raw protein abundance data were processed using the R statistical computing environment (v4.3). To account for missing values characteristic of label-free proteomics, typically representing peptides below the limit of detection, we applied a columnwise minimum imputation strategy. Specifically, missing values were replaced with 10% of the minimum observed intensity value within each respective sample column

$${\text{Impute}}_{\text{Val}}=0.1\times {\text{Min}}_{\text{sample}}$$

Following imputation, intensities were log_2_ transformed with a pseudocount of 1 [log_2_(*x* + 1)] to stabilize variance and normalize the distribution for downstream statistical analysis.

#### Quality control and exploratory analysis

Data integrity and sample reproducibility were evaluated using a comprehensive multifaceted approach.

#### Correlation analysis

Sample similarity was assessed using Pearson correlation coefficients calculated on log_2_-transformed data. Correlation matrices were visualized as heatmaps using the pheatmap R package. Within-condition correlations were calculated to evaluate technical reproducibility among biological replicates.

#### Dimensionality reduction

Principal components analysis was performed on centered and scaled data to visualize global sample separation and identify potential batch effects. Variance explained by each principal component was calculated and visualized using scree plots. Multidimensional scaling was conducted using Euclidean distances as a complementary approach to verify group clustering patterns.

#### KD efficiency and specificity analysis

KD efficiency for target synaptic proteins was quantified by calculating the ratio of mean intensity in KD samples relative to WT controls. Percentage KD was defined as

$$\%\text{Knockdown}=100-\left({\text{Mean}}_{\text{KD}}/{\text{Mean}}_{\text{WT}}\times 100\right)$$

### TEM sample preparation and imaging

After activity imaging, imaging medium (ACSF) was aspirated, and cells were rinsed twice with 0.1 M phosphate buffer at RT. Next, 1 ml of warm EM fixative [2% glutaraldehyde and 2 mM CaCl_2_ in 0.1 M SC buffer (pH 7.2)] was gently added, and cells were incubated on ice for 1 hour. Fixative was then aspirated and neutralized with ice-cold glycine (20 mM) in 0.1 M SC buffer. Cells were thoroughly rinsed with ice-cold 0.1 M SC buffer, followed by addition of DAB (0.5 mg/ml) and 10 mM H_2_O_2_ in ice-cold 0.1 M SC buffer. Cells were incubated on ice for 25 min and washed thoroughly with fresh ice-cold 0.1 M SC containing 2 mM CaCl_2_. EM processing began with postfixation in 2% OsO_4_, 1.25% K_4_Fe(CN)_6_, and 3 mM CaCl_2_ in 0.15 M sodium cacodylate for 30 min on ice. After rinsing with 0.1 M SC buffer containing 2 mM CaCl_2_, cells were incubated with 1% uranyl acetate in water as the positive stain for 2 days at 4°C. Uranyl acetate solution was gently aspirated, and cells were rinsed three times with ice-cold Milli-Q water. Serial ethanol dehydrations were carried out using ice-cold solutions (30, 50, 70, and 90% ethanol; 7 min each). Cells were brought to RT in 90% ethanol and further dehydrated with three exchanges of 100% ethanol (7 min each) at RT.

Cells were infiltrated with Epon resin in ethanol at 1:2, 1:1, and 2:1 (v/v) ratios for 90 min each at RT. Cells were then incubated overnight in 100% Epon resin containing a reduced amount of accelerator. The next day, cells underwent three exchanges of 100% Epon resin with the manufacturer-recommended accelerator amount, at 2-hour intervals. Excess Epon was removed from the dish, and coverslips were gently detached and placed cells up onto an Aclar sheet (EMS, catalog no. 50426-10) supported by a heat-resistant base. A labeled BEEM capsule (Ted Pella, product no. 130) filled with Epon resin was very carefully inverted over the region of interest (ROI) on the coverslip. Polymerization was proceeded at 60°C for 48 hours. Postpolymerization, coverslips were separated from the BEEM capsule by alternating brief submersion in liquid nitrogen and hot water. Grid markings etched onto the block face facilitated locating the region and neurons of interest. Blocks were trimmed and serially sectioned (50 nm) using a Leica UC6 ultramicrotome, with thin sections collected on formvar coated slot grids. The ROIs were mapped to the activity patterns via low-magnification imaging (80 keV) on a Tecnai G2 Spirit BioTWIN transmission electron microscope using a Gatan OneView camera (4k × 4k resolution). Higher-magnification TEM images were acquired for detailed analysis. Automatic detection and quantification of synaptic vesicle parameters were performed in Python 3.12 using a published algorithm available at https://github.com/Imbrosci/synaptic-vesicles-detection. Small FOVs that include DAB^+^ and DAB^−^ boutons (8-bit TIFF images) were analyzed, and statistical tests were performed in Prism10 (GraphPad software).

### Volume EM and organelle segmentation

After activity imaging, cultured cells were rinsed twice with 0.1 M phosphate buffer at RT. Then, 1 ml of warm EM fixative [2% glutaraldehyde and 2 mM CaCl_2_ in 0.1 M SC buffer (pH 7.2)] was gently added, and cells were incubated for 2 hours on ice. After washing the sample twice with ice-cold 0.1 M SC buffer, secondary fixation was carried out with 2% OsO_4_ and 1.25% potassium ferrocyanide in 0.1 M SC buffer for 30 min on ice. Cells were washed again with ice-cold 0.1 M SC buffer and fixed with 1% OsO_4_ in 0.1 M SC buffer for 30 min on ice. After rinsing thoroughly with 0.1 M SC buffer, cells were incubated with 1% uranyl acetate in water overnight at 4°C. Next day, cells were washed with ice-cold water and dehydrated using a series of ethanol dilutions (30, 50, 70, and 90%) for 7 min each on ice. Samples were brought to RT and treated with 100% ethanol 3× for 10 min (with molecular sieves). Next, cells were infiltrated with Eponate 12 resin (Ted Pella, 18012) through a graded series of Epon:ethanol mixtures—1:2 for 1 hour, 1:2 for 1.5 hours, and 2:1 for 1.5 hours—and lastly polymerized in 100% Epon for 24 hours at 60°C. The ROI was trimmed and mounted on a copper stub with Durcupan (Sigma-Aldrich, MO) polymerized for 24 hours at 60°C. The vertical sample post was trimmed to the ROI (84.4 μm by 105.7 μm) and a depth of 83.0 μm in the direction of the ion beam. The trimming was guided by x-ray tomography data obtained by a Zeiss Versa XRM-510 and optical inspection under a microtome. All samples were imaged by a Zeiss Merlin FIB-SEM system and generated volumes composed of isometric 6 nm–by–6 nm–by–6 nm voxel sizes. Dataset was segmented by Ariadne.ai and proofread by the Janelia project pipeline support team. Synaptic vesicles were manually annotated as 40-nm spheres in neuroglancer for visualization purposes.

### ExM sample preparation and data analysis

Tissue preparation and gel recipe for ExM were performed according to a previously published procedure (*49*). Briefly, ALFADoR1 and ALFADoR2 mice were anesthetized with isoflurane and perfused transcardially with an ice-cold solution of 4% PFA and 10% acrylamide in 1× PBS. After overnight postfixation in the same fixative, brains were sectioned coronally into 100-μm slices, and excess PFA was quenched with 100 mM glycine. Tissue sections containing prefrontal cortex, striatum, and amygdala were selected for the study. After the first round of polymerization, tissue-gel pieces were probed using rabbit anti-TH and human anti-ALFA antibodies at 1:300 dilutions at 4°C overnight with gentle shaking. Donkey anti-rabbit CF633 and goat anti-human AF546 secondary antibodies were used at 1:300 dilutions, and probing were carried out overnight at 4°C. ExM images were collected on Nikon spinning disk microscope, using a 40× water immersion objective (1.25 NA).

Typical imaging volumes included FOVs that were ∼375 μm by 375 μm in the *xy* plane and 50 to 100 μm in the *z* direction, taken at 0.5-μm *z*-stacks. Each FOV contained two channels: a dopamine axon channel (dopamine axons with varicosities) and an ALFA-tagged receptor channel (D1-ALFA or D2-ALFA). Images were analyzed in MATLAB using a custom pipeline designed to quantify nanoscale apposition between dopamine varicosities and receptor clusters in 2D. Images were converted to double precision and normalized. A light Gaussian smoothing step was applied to stabilize segmentation, using 0.5 sigma in pixels. The dopamine and receptor channels were processed independently up to the point of apposition analysis.

Dopamine axons were first segmented to define an axonal domain that constrained subsequent varicosity identification. The dopamine channel was enhanced to emphasize elongated axon-like structures and then thresholded to generate an initial binary axon mask. Morphological cleanup steps were applied to close small gaps, fill holes, and remove small, isolated components. Varicosities were then segmented using a watershed-based approach, which improved granularity and avoided merged varicosity objects in densely labeled axons when compared with other segmentation methods that we tried. Within the axon mask, the dopamine channel was smoothed and thresholded to identify bright candidate regions. To separate adjacent bright structures, watershed segmentation was applied to a topography derived from the intensity image such that local intensity peaks were resolved into distinct objects. The resulting objects were filtered by minimum size, producing a final varicosity mask. For each segmented varicosity, geometric descriptors including centroid position, area, and perimeter were extracted using connected-component labeling and region properties. These per-varicosity measurements were used for both spatial apposition quantification and downstream distributional analyses.

Receptor clusters were segmented from the ALFA channel using a complementary watershed-based puncta pipeline. The receptor image was smoothed, a candidate bright mask was generated by thresholding, and watershed was applied to separate adjacent puncta. An *h*-minima suppression step was used to reduce oversegmentation by removing shallow intensity minima before watershed. Segmented puncta were then filtered by a minimum area threshold. To control stringency and suppress dim detections, puncta were optionally filtered by requiring that each object’s peak intensity exceed a user-defined fraction of the maximum image intensity. For each punctum, centroid position and morphological features (including area, major axis length, minor axis length, eccentricity, and perimeter) were quantified.

Apposition between dopamine varicosities and receptor puncta was quantified using a perimeter-based minimum distance criterion designed to reflect near contact between the boundaries of segmented structures. In our analysis, we defined apposition as proximity of less than 0.125 μm, which corresponded to 3 pixels in the expanded gel images, but additional appositional analysis was performed at larger threshold distances of 0.25, 0.35, and 0.5 μm, corresponding to camera pixels of 6, 9, and 13 in expanded gels, as shown in Fig. 7G. A distance transform was computed from the receptor puncta mask to the nearest receptor pixel. For each varicosity, the analysis extracted the varicosity perimeter and computed the minimum distance from any perimeter pixel to the receptor mask. Varicosities were classified as “apposed” at a given threshold if this minimum distance was less than or equal to the threshold (in pixels). In addition to binary classification, a fractional contact metric was computed for each varicosity at each threshold, defined as the fraction of varicosity perimeter pixels lying within the threshold distance to the receptor mask. These per-object values were aggregated across varicosities to generate apposition fractions per imaging FOVs.

To determine whether the observed apposition exceeded what would be expected from receptor density and spatial heterogeneity alone (i.e., appositional encounters by chance alone), we implemented a conservative null model on the basis of toroidal shuffling. For each permutation, the receptor puncta mask was translated by a uniformly random (*x*, *y*) offset with wraparound at image boundaries. Shifting (*x*, *y*) values were randomly chosen such that 0 < *x*_shift_ < ImagePixelSize in the *x* direction, and the same approach was used for shifts in the *y* direction. This procedure preserves the number, size, and internal geometry (for example, noise and autocorrelation) of puncta, as well as any broad spatial inhomogeneities in receptor labeling, while disrupting spatial alignment to dopamine axons and varicosities. For each shuffled run, the same varicosity-to-receptor perimeter distance analysis was repeated and the resulting apposition metrics computed and stored. Null means and CIs were computed from the permutation results, and *P* values were reported by comparing the experimental values to the shuffled distribution. These shuffled controls were used throughout to benchmark “apposition by chance” expectations and to support our hypothesis that proximity reflects structured coupling rather than random overlap.

Several complementary spatial summaries were computed from the same segmented objects. Nearest-neighbor distance distributions were evaluated by measuring, for each varicosity centroid, the distance to the nearest receptor punctum centroid, and, reciprocally, for each receptor punctum centroid, the distance to the nearest varicosity centroid. These distances were used to generate CDFs for each FOV and to compare distributions across brain regions and receptor types (as shown in Fig. 7, M and N).
