# Supplementary material for: Synapse-like specializations at dopamine release sites orchestrate efficient and precise neuromodulatory signaling
Source: Sci Adv. 2026 Oct 2;12(40):eaei0203. doi: 10.1126/sciadv.aei0203 (PMC13632744; doi:10.1126/sciadv.aei0203)
Supplement: Supplementary file 1 [file sciadv.aei0203_sm.pdf]

Science Advances
AAAS

Supplementary Materials for

Synapse-like specializations at dopamine release sites orchestrate efficient and precise neuromodulatory signaling

Chandima Bulumulla *et al.*

Corresponding author: Abraham G. Beyene, beyenea@janelia.hhmi.org

Sci. Adv. **12**, eaei0203 (2026)
DOI: 10.1126/sciadv.aei0203

The PDF file includes:

Tables S1 to S4
Legends for movies S1 and S2
Figs. S1 to S18

Other Supplementary Material for this manuscript includes the following:

Movies S1 and S2

Supplementary Tables

Table S1. List of chemical reagents.

Chemical	Abbreviation	Vendor	Identifier
Single-walled carbon nanotubes	SWCNT	NanoIntegris	HR35-141
5'-GTG TGT GTG TGT-3'	$(GT)_6$	IDT	N/A
Dopamine hydrochloride	DA	Sigma-Aldrich	H8502
(3-Aminopropyl) triethoxysilane	APTES	Sigma-Aldrich	440140
Ethanol	EtOH	Fisher Scientific	A405P
32% Paraformaldehyde	PFA	EMS	15714
Glycine	N/A	Sigma-Aldrich	G7126
Triton X-100	N/A	Fisher Scientific	BP151
Tween-20	N/A	Sigma-Aldrich	P7949
NbActiv4	N/A	Transnet/ Brain Bits	NC1477957
Acrylamide	AA	Sigma-Aldrich	A9099
Sodium acrylate	SA	AK Scientific	R624
N,N'-Methylenebisacrylamide	Bis	Sigma-Aldrich	M7279
Ammonium persulfate	APS	Bio Rad	1610700
Sodium bicarbonate	$NaHCO_3$	Oakwood Chemicals	099274
N,N,N,N'-Tetramethylethylenediamine	TEMED	Bio Rad	161-0800
Sodium azide	N/A	Sigma-Aldrich	71289
Glycidyl glycerol ether	TGE	Polysciences Europe GmbH	09221
Glycidyl methacrylate	GMA	TCI	G0497
Sodium chloride	NaCl	Fisher Scientific	190383
4,6'-diamino-2-phenylindole dihydrocholride	DAPI	Sigma-Aldrich	D9542
4-(2-Hydroxyethyl)piperazine-1-ethanesulfonic acid	HEPES	Sigma-Aldrich	H3375
20X Sodium-saline citrate buffer	20X SSC	Invitrogen	AM9763
(Tris(hydroxymethyl)aminomethane)	1M Tris pH 8	Invitrogen	AM9856
D-Glucose	N/A	Acros Organics	41095
Sodium phosphate monobasic	NaH_2PO_4	Sigma-Aldrich	P9791
Sodium cacodylate trihydrate	SC	Sigma-Aldrich	C0250
Osmium tetraoxide	OsO_4	Electron microscopy Sciences	19150
Potassium ferrocyanide	$K_4Fe(CN)_6$	Macron	6932-04
Uranyl acetate	N/A	Ibi Labs	U73493-25-ACS
Eponate 12™ kit		Ted Pella	18012
(3-(N-Morpholino)propane sulfonic acid)	MOPS	Fisher Scientific	BP308
Melphalan	N/A	Cayman Chemicals	16665
(6-((acryloyl)amino)hexanoic acid, succinimidyl ester)	AcX	ThermoFisher Sci.	A20770
Isoflurane	N/A	Fisher Scientific	50-304-2993
poly-L-lysine	N/A	Pelco	18026
Poly-D-lysine	N/A	Sigma-Aldrich	P6407
Bovine serum albumin	BSA	Sigma-Aldrich	A9418
Calcium chloride	$CaCl_2$	Acros Organics	349615000
Magnesium sulfate	$MgSO_4$	Fisher Scientific	M65
Potassium chloride	KCl	Fisher Scientific	P217
Glutamine	N/A	Fisher Scientific	25-030-149
Heat inactivated fetal bovine serum	FBS	Fisher Scientific	SH30071.03

MEM media	N/A	Invitrogen	51200-038
Transferrin	N/A	Sigma-Aldrich	616420
Insulin	N/A	Sigma-Aldrich	I-6634
Papain enzyme	N/A	Worthington Biochemical	PAP2
Drd1 and Drd2 HCR Probes	N/A	Molecular Instruments	N/A
RNAScope probes (Drd1/2) and reagents	N/A	Advanced Cell Diagnostics	N/A
Opal 520 and Opal 690	N/A	Akoya Biosciences	N/A
Water	N/A	Fisher Scientific	W6-4
Acetonitrile	ACN	Fisher Scientific	A9554
Methanol	N/A	Fisher Scientific	A454SK-4
Formic acid	N/A	Pierce	PI28905
Acetone	N/A	Fisher Scientific	A949-1
RapiGest SF surfactant	N/A	Waters	186001861
Dithiothritol	DTT	Thermo Scientific	R0861
Trifluoroacetic acid	TFA	Sigma-Aldrich	302031
Iodoacetamide	IAA	Sigma-Aldrich	407710
Ammonium bicarbonate	N/A	Sigma-Aldrich	40867
Trypsin	N/A	Promega	V5113

Table S2. List of primary antibodies

Target	Vendor	Identifier	Host species	Dilution factor
Tyrosine hydroxylase (TH)	Aves Labs	TYH 67677979	Chicken	1:1000
Tyrosine hydroxylase (TH)	Sigma-Aldrich	AB152	Rabbit	1:1000
Bassoon (Bsn)	Abcam	Ab82958	Mouse	1:1000
RIM1/2	Synaptic Systems	SySy 140 205	Guinea pig	1:1000
Munc13-1	Synaptic Systems	SySy 126 103	Rabbit	1:1000
Synaptophysin1 (Syp)	Synaptic Systems	SySy 101 308	Guinea pig	1:1000
Synaptobrevin2 (Vamp2)	Synaptic Systems	SySy 104 318	Guinea pig	1:1000
ALFA antibody	NanoTag Biotech	N1586	Human	1:300
ALFA nanobody Atto643	NanoTag Biotech	N1502-At643-L	Alpaca	1:200
RFP	ThermoFisher	600-401-379	Rabbit	1:1000
MAP2	Novus biologics	NB300-213	Chicken	1:1000
GFAP	Sigma-Aldrich	G3893	Mouse	1:1000
Neuroligin2 (NL2)	Synaptic Systems	SySy 129 511	Mouse	1:1000

Table S3. List of secondary antibodies

Target	Host	vendor	Identifier	Fluorophore	Dilution factor
Chicken IgY (H+L)	Donkey	Jackson Immuno.	703-675-155	BV 421	1:1000
Chicken IgY (H+L)	Goat	ThermoFisher Sci.	A78948	AF Plus 488	1:2000
Chicken IgY (H+L)	Goat	ThermoFisher Sci.	A32933TR	AF Plus 647	1:2000
Mouse IgG/IgM (H+L)	Goat	ThermoFisher Sci.	A10680	AF 488	1:2000
Mouse IgG (H+L)	Goat	ThermoFisher Sci.	A32728TR	AF 647	1:2000
Rabbit IgG (H+L)	Goat	ThermoFisher Sci.	A11034	AF 488	1:2000
Rabbit IgG (H+L)	Goat	ThermoFisher Sci.	A11037	AF 594	1:2000
Rabbit IgG (H+L)	Goat	Abberior	STRed-1002	STAR Red	1:2000
Rabbit IgG (H+L)	Donkey	Biotium	#20125	CF 633	1:2000
Guinea pig IgG (H+L)	Goat	ThermoFisher Sci.	A11073	AF 488	1:2000
Guinea pig IgG (H+L)	Goat	Abberior	STRed-1006	STAR Red	1:2000
Human IgG (H+L)	Goat	ThermoFisher Sci.	A21089	AF 546	1:300

Table S4. Forward and reverse primer PCR sequences of Drd1 and Drd2 genes

Gene	Target	Forward	Reverse
Drd1	5’-junction	TGGAGCACTGAACCCAGAAG	CCAGTCTCATCCATGGTAGA
	3’-junction	CTAAGCCACCGGAAGTGCTT	CGTGGAGCACATGATGTCAA
Drd2	5’-junction	GAAAGAATGGATGAGTTAGCTC	GATCATCATCGTACCAGGAC
	3’-junction	GAAAGAATGGATGAGTTAGCTC	AGTGTGGCCACCAGAAGATC

Supplementary Movies:

Movie S1: Dopamine axons innervate a putative soma in a DA:C co-culture and release dopamine.

Movie S2: FIB/SEM of dopamine axons innervating a soma.

Supplementary Figures:

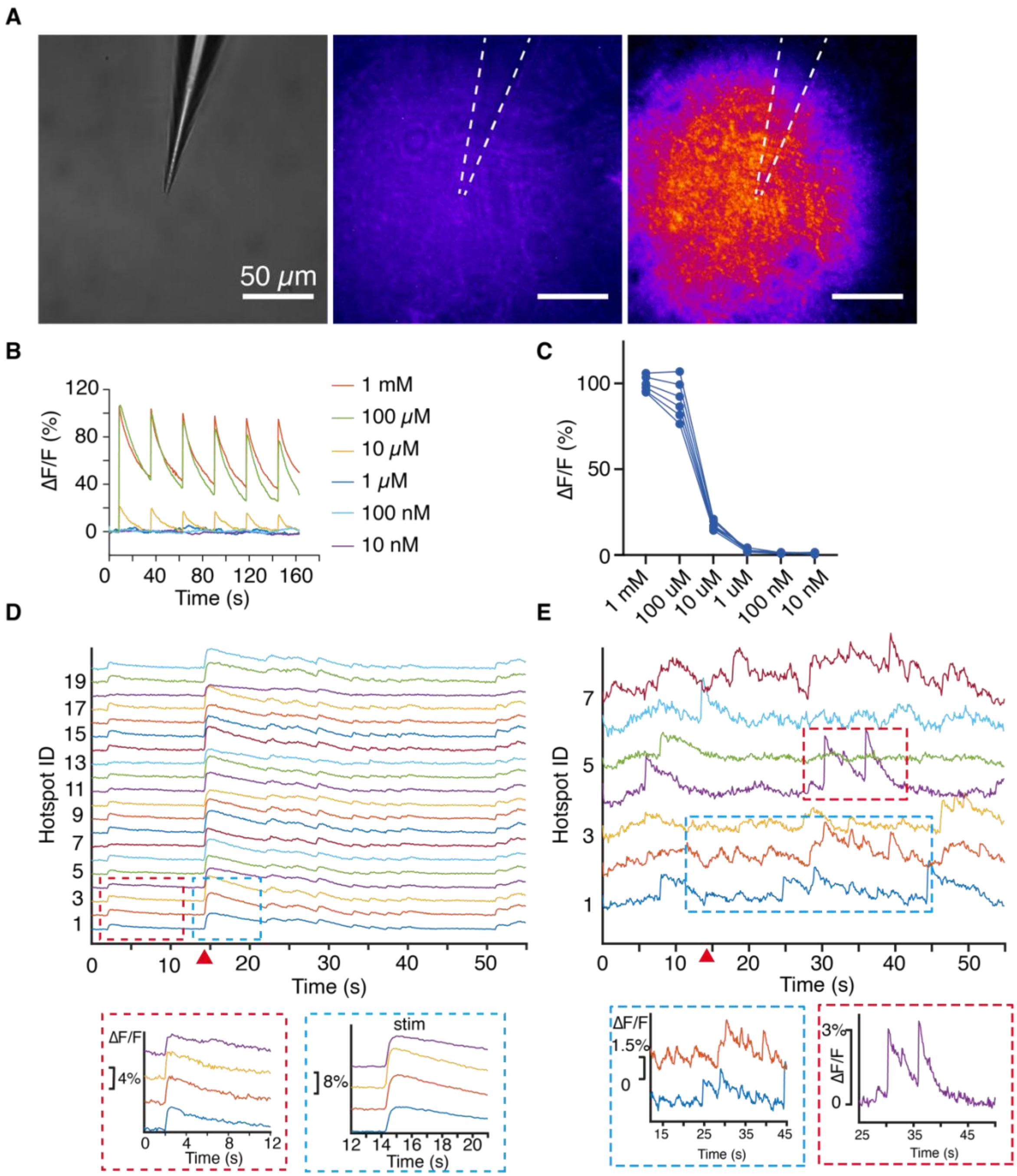

Figure S1. Calibration and sensitivity of DopaFilm.

(A) Transient dopamine puff application elicits a spatially uniform fluorescence response across DopaFilm, demonstrating sensor uniformity.
(B) Representative temporal responses to six 50-ms dopamine puffs from pipettes placed ~1–2 µm from the film surface, delivered over a range of dopamine concentrations. DopaFilm exhibits rapid, reversible, and concentration-dependent fluorescence responses.
(C) Peak ΔF/F responses as a function of dopamine concentration. Each trace represents response from six repeated puff applications performed on a single DopaFilm preparation.

(D) Representative activity traces showing both spontaneous (self-autonomous, red box) and electrically evoked (blue box) dopamine release events detected by DopaFilm. Red wedge indicates time of field stimulation.
(E) Tetrodotoxin (TTX, 100 nM) abolishes electrically evoked dopamine transients while spontaneous dopamine release events remain detectable, demonstrating that DopaFilm possesses sufficient sensitivity to detect low-amplitude spontaneous vesicle fusion events. Red wedge indicates time of field stimulation.

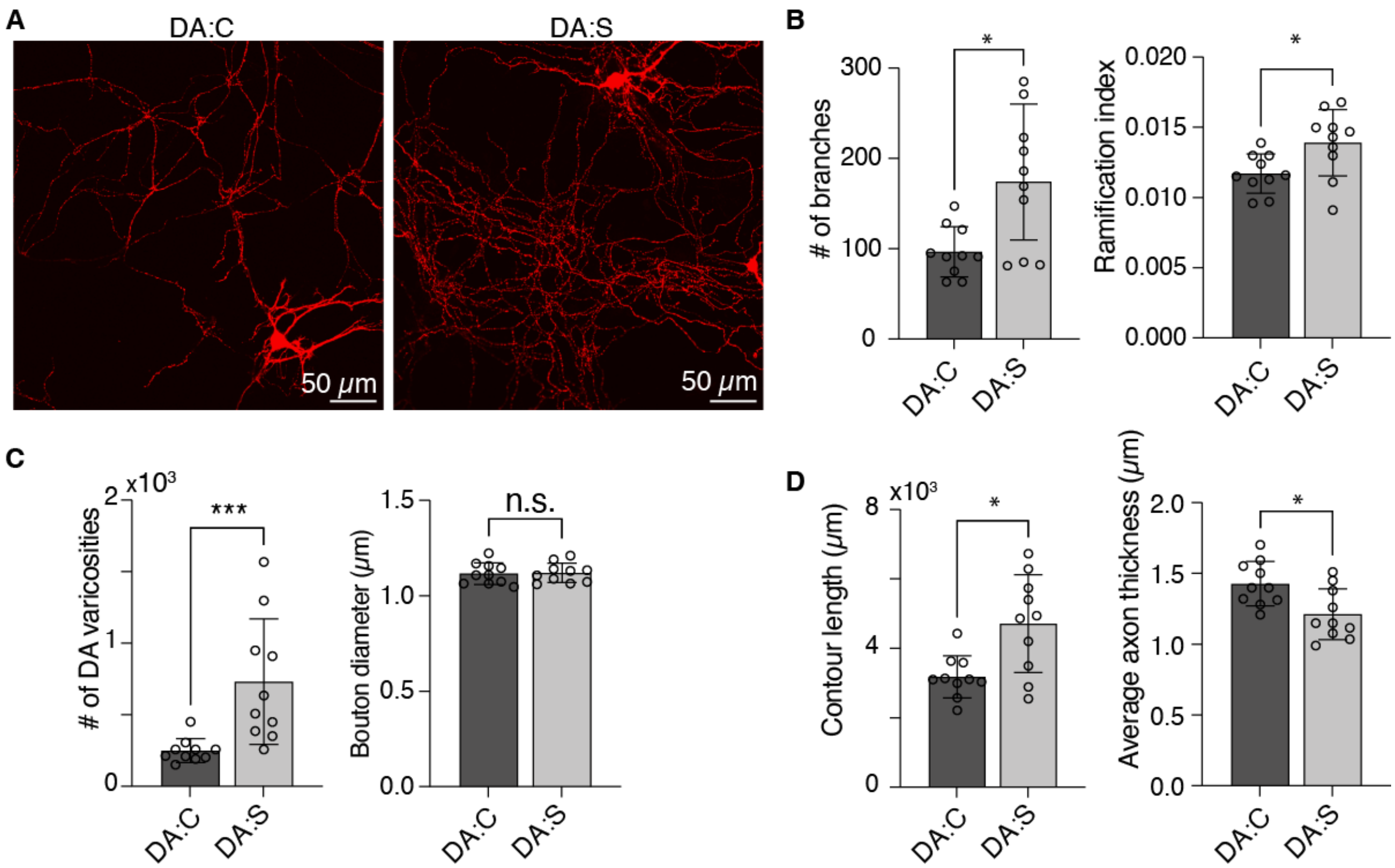

Figure S2. DA:C and DA:S cultures exhibit morphological differences that mirror reported in vivo characteristics.

(A) Representative dopamine axonal arborization in an imaging field of view corresponding to DA:C and DA:S co-cultures. Note that DA:S cultures generate highly ramified dopaminergic axonal processes.

(B–D) Analysis of arborization (B), varicosity (C) and axonal morphometrics (D). Each data point represents average across an independent FOV belonging to one dopamine neuron. Mean ± SD bars are shown. Test of statistical significance: Mann-Whitney test. p-values: n.s. = not significant, * < 0.05, *** < 10^{-3}. Ramification index = # of branches per unit axon length.

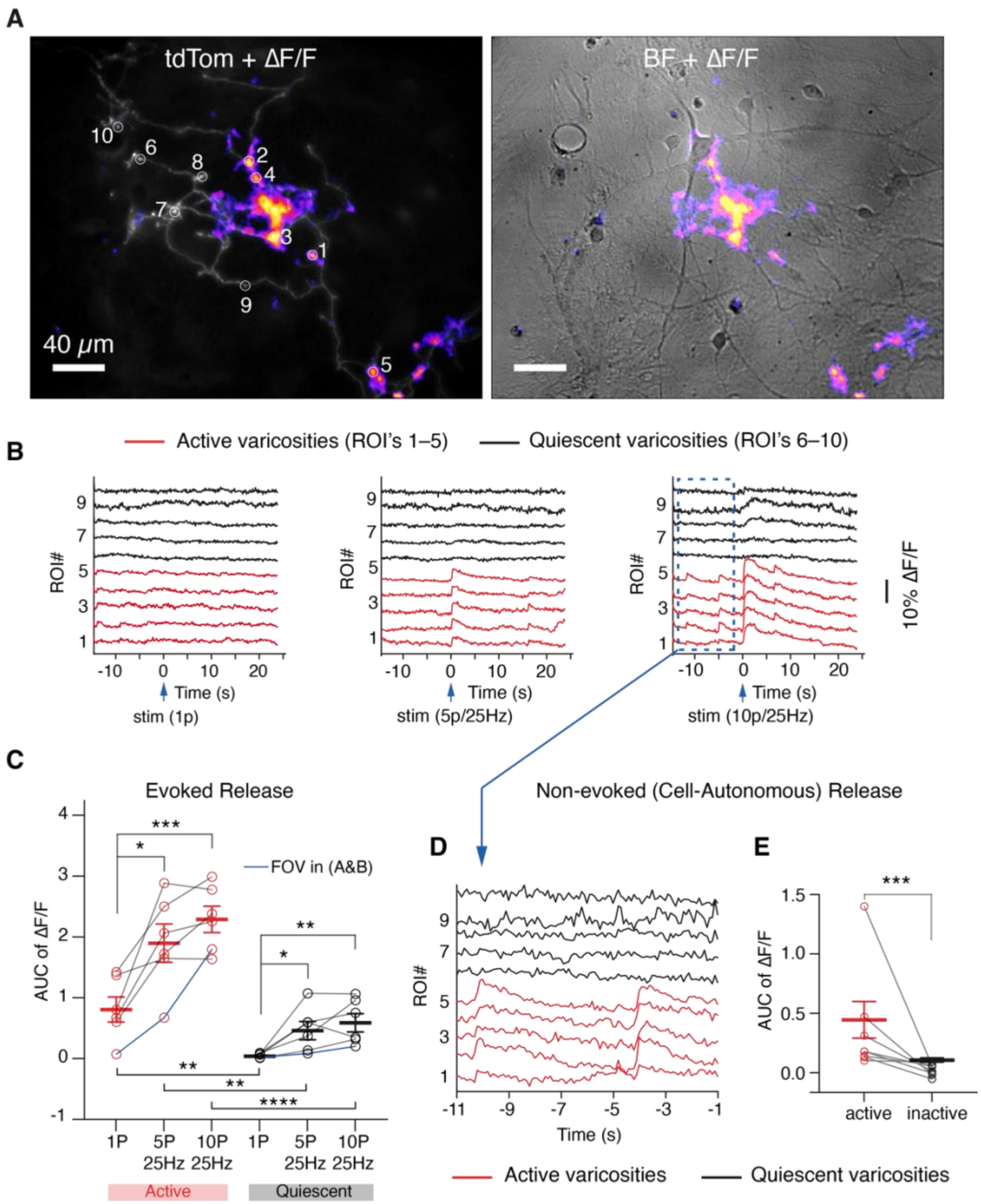

Figure S3. Characterization of active and quiescent varicosities.

(A) An imaging field of view showing dopamine axons (tdTom) overlaid with dopamine release activity heatmap (ΔF/F). Right: brightfield (BF) image of the same FOV shown on the left.

(B) Activity traces corresponding to the varicosities (ROI's 1–10) depicted in (A) corresponding to single pulse (left), 5-pulse (middle) and 10-pulse (right) electrical field stimulation.

(C) Analysis of area under the curve (AUC) of ΔF/F trace over a 3-second window after stimulation. Each data is an average across a field of view of imaging from n = 10 randomly sampled active and quiescent varicosities; mean±SEM bars are shown. The blue trace represents data from the field of view/data shown in (A&B). Test of statistical significance: Kolmogorov-Smirnov (KS) test. p-values: * < 0.05, ** $< 10^{-2}$, *** $< 10^{-3}$, **** $< 10^{-4}$

(D) Close-up of non-evoked release window corresponding to the 10-pulse stimulation event shown in (B). Note that quiescent varicosities remain release-incompetent during non-evoked release events.

(E) Analysis of AUC of non-evoked release events such as that shown in (D). Each point is an average from an independent field of view, and mean ± SEM bars are shown. Test of statistical significance: KS test. p-value: *** < 10^{-3}

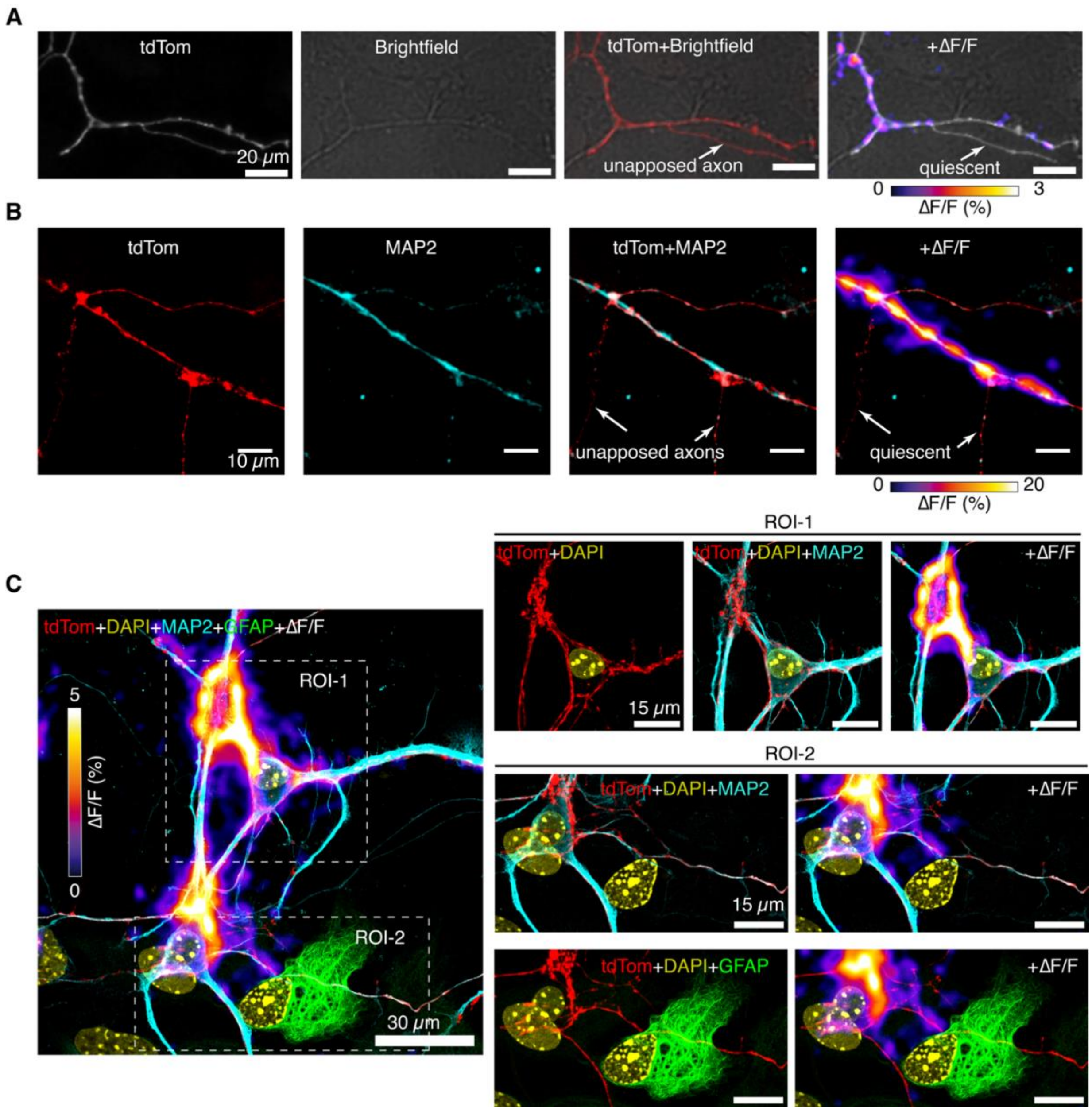

Figure S4. Apposition to neuronal processes is necessary for release-competence.

(A) Live-cell imaging: a dopamine axonal process (tdTom) and corresponding bright field image, overlay between tdTom and brightfield, and overlay with dopamine release (ΔF/F) are shown. Notice the process in brightfield with which the dopamine axon is associating/co-mingling. A non-apposed axonal branch does not participate in release.

(B) Immunocytochemistry staining for MAP2: only dopamine axons (tdTom) that are dendritically apposed participate in release. Notice that non-apposed axonal branches do not participate in release.

(C) Immunocytochemistry staining for MAP2 and GFAP: MAP2 apposed axonal processes participate in release whereas GFAP-associating axons remain quiescent.

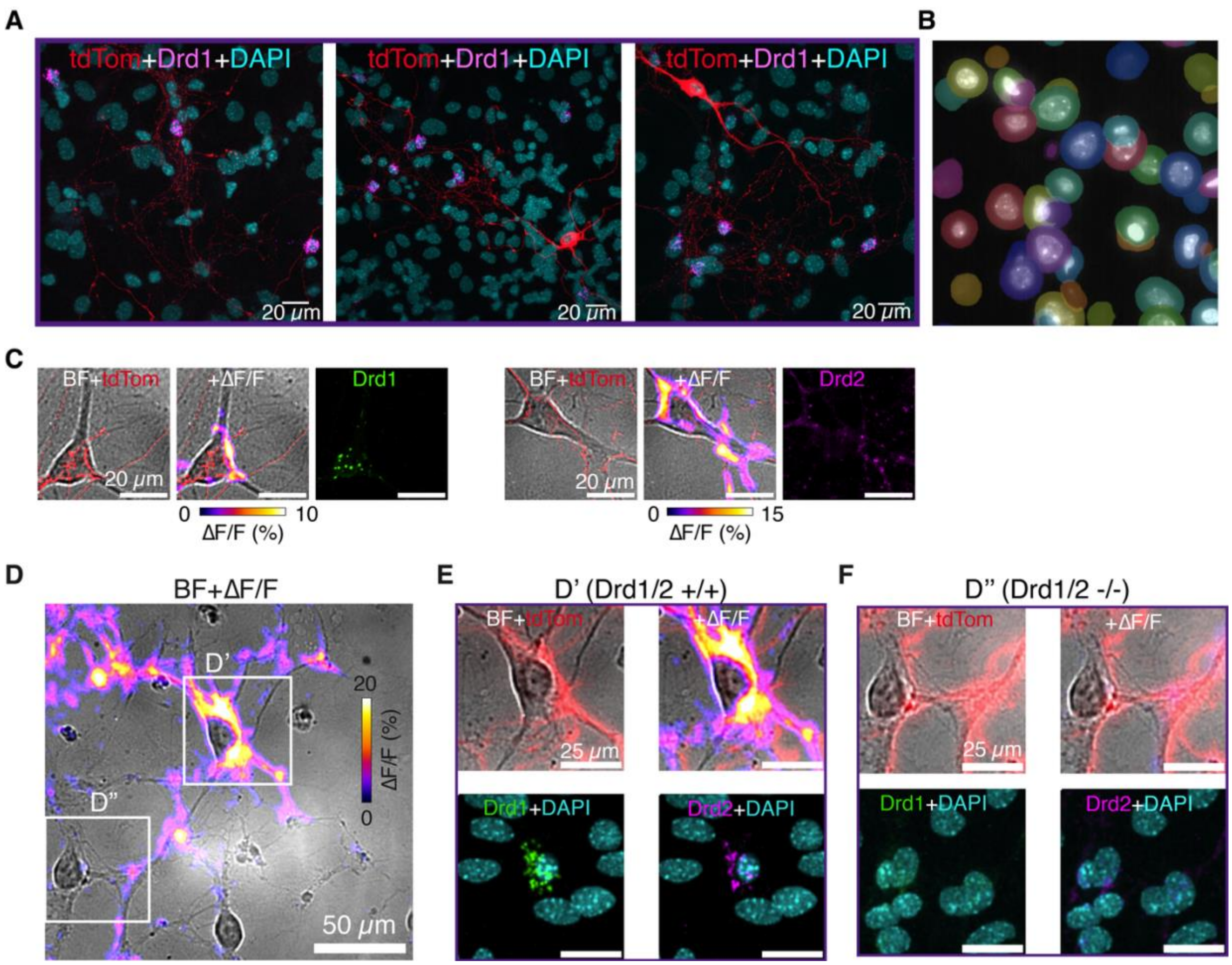

Figure S5. Transcriptomic profiling of dopamine receptors in culture and tissue shows tropism of dopamine axons towards Drd1 or Drd2-positive nuclei.

(A) Three example fields of view showing dopamine axons (red, tdTom) preferentially innervating DAPI nuclei (blue) that are positive for Drd1 (magenta).

(B) An example of a FOV containing Cellpose-segmented DAPI-nuclei used in analysis of tropism in dopamine axons in tissue.

(C) Example Drd1 and Drd2 soma in brightfield, overlayed with Drd1 (green puncta) and Drd2 (magenta puncta) readout from RNAScope. Dopamine release heatmap shows secretion of dopamine onto soma.

(D) A FOV of imaging showing brightfield image (BF) and dopamine release (ΔF/F).

(E, F) Close up of D' and D" (from panel D) showing tdTom, ΔF/F heatmaps, DAPI stain and readout from RNAScope. Neuron in D' is positive for both Drd1 and Drd2 whereas the neuron in D" is negative for both. Note the absence of dopamine release in F" despite some innervation by dopaminergic axons.

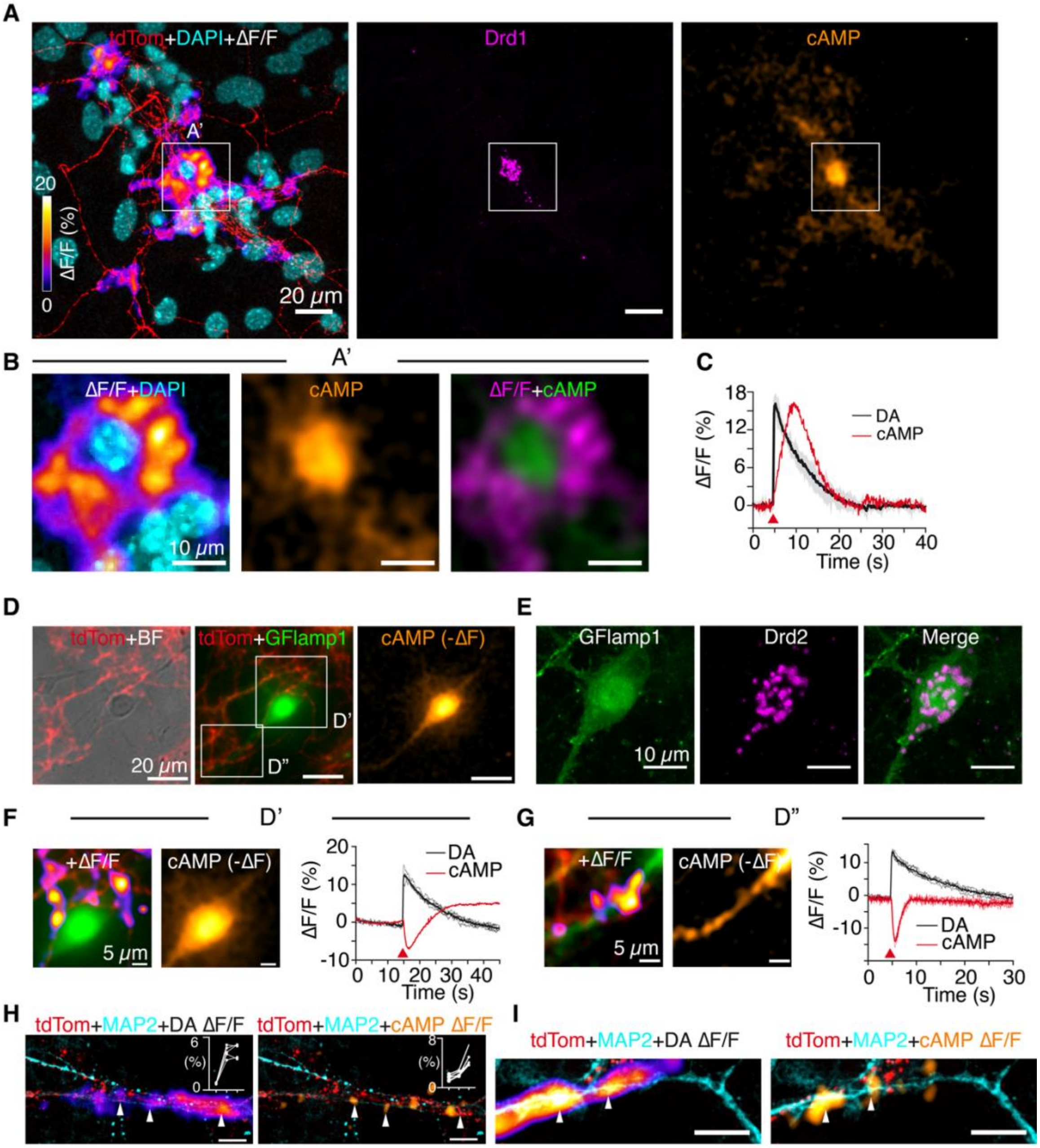

Figure S6. Dopamine release rapidly modulates cAMP levels in target neurons.

(A) A three-panel figure showing tdTom, DAPI and ΔF/F, Drd1 RNA transcripts probed by RNAScope, and cAMP ΔF/F frame. Dopamine and cAMP peak ΔF/F images are shown, which occur at different time points after application of stimulus (see panel C). Note that this field of view is identical to one used in Fig. 3A–C.

(B) Magnified view of A' depicted in the solid white box in (A). Overlaid images of ΔF/F and DAPI, and cAMP ΔF frame (orange), and a merge of dopamine ΔF/F (magenta) and cAMP ΔF (green) are shown. An extracellular

dopamine “corona” elicits a vigorous intercellular cAMP activity.

(C) Temporal traces of dopamine (black) and cAMP (red) for images shown in (B) are shown. Dopamine is presented as an average trace of identified hotspots and cAMP activity is averaged over the soma.

(D–E) A representative example of a dopaminergic axonal innervation of a cortical neuron exhibiting negative cAMP modulation at the soma and dendritic shafts. The cAMP pseudo color is inverted for display purposes. The neuron was confirmed to be positive for Drd2 transcripts using post hoc RNA FISH as shown in (E).

(F–G) Zoomed-in images of white box ROIs depicted in (D), along with temporal traces showing dopamine and cAMP activity.

(H–I) cAMP activity hotpots generated by dopamine hotspots in distal dendritic processes in DA:C co-cultures. Note that dopamine activity and cAMP activity are spatially co-located. Insets in (H): DA and cAMP peak ΔF/F scale with stimulation (2 pulses, 5 pulses and 10 pulses from left to right, all at 25 Hz).

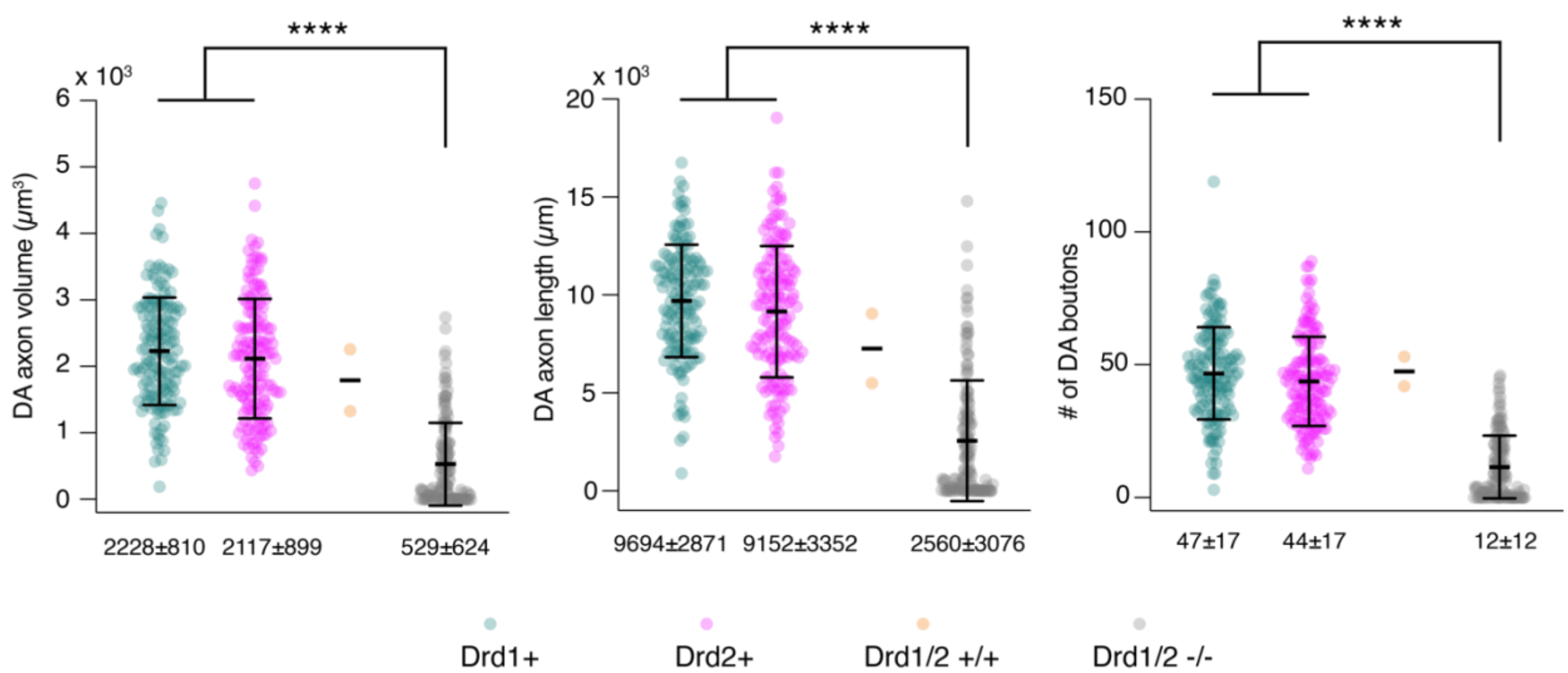

Figure S7. Analysis of EASI-FISH volume in the dorsal striatum.

Total axon volume, length and number of boutons are evaluated. Each point (n = 150 for three groups) is a DAPI+ nucleus and dopamine axons that associate with the soma (approximated by the nucleus with some dilation, see Methods) are segmented and analyzed. Mean ± SD bars and values are shown at the bottom. A two-sample KS-test was used to evaluate significance of differences between transcript-positive vs. negative groups (group-wise); p-value: **** < 10^{-4}. See Methods for more on analysis of EASI-FISH volume.

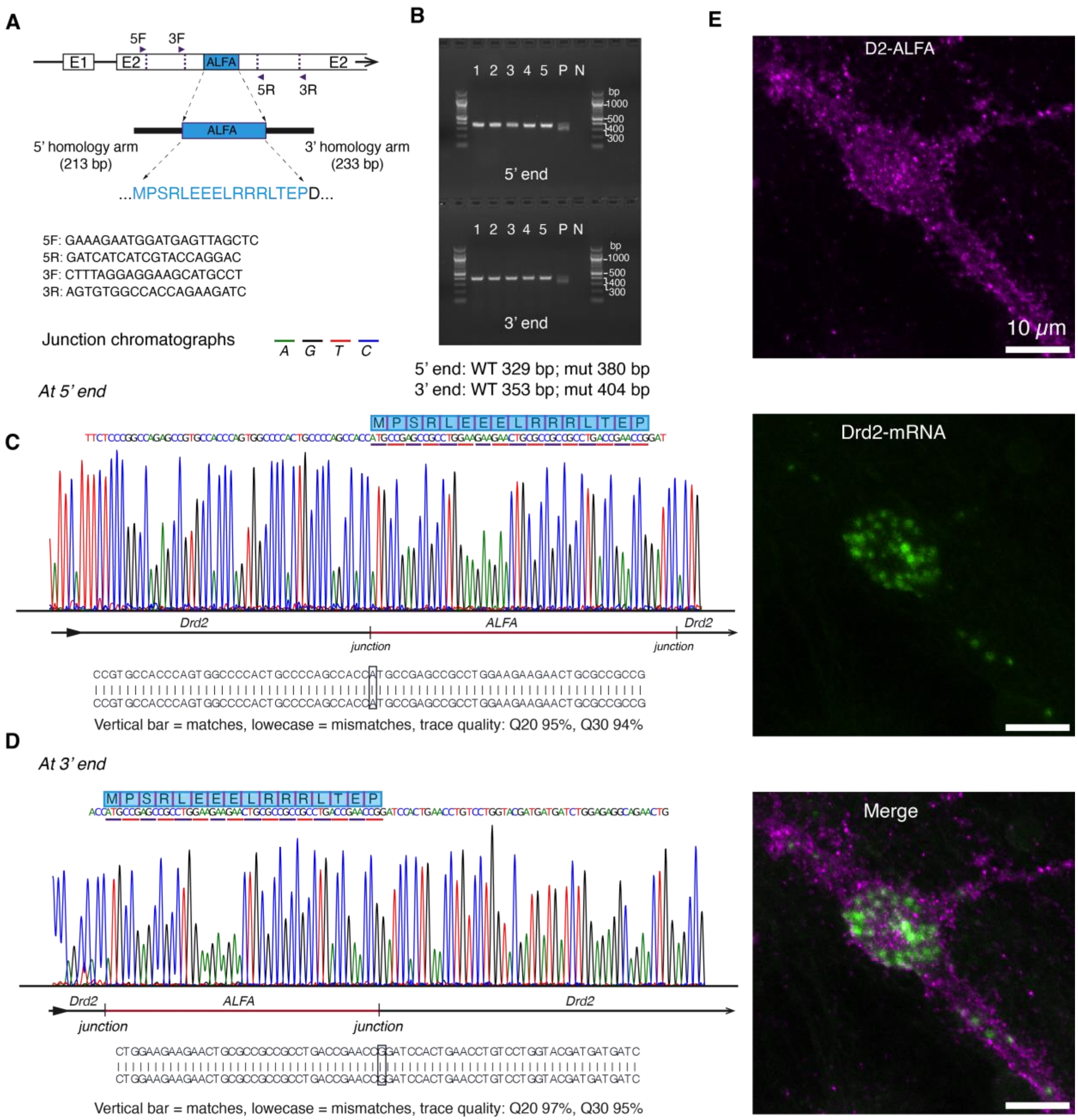

Figure S8. Design and validation of ALFA-tag knock in into the Drd2 gene locus.

(A) The design schematic of ALFA-tag insertion into the second exon (E2) of Drd2 gene, with 213 bp and 233 bp homology arms on the 5' and 3' ends respectively. 5' and 3' genotyping primers are shown.

(B) Gel electrophoresis of PCR products confirming correct integration at both the 5′ and 3′ junctions. Five biological replicates are shown. The positive control (P), containing a mixture of wild-type (WT) and knock-in alleles (mut), displays two closely migrating bands corresponding to the WT and mut products. The negative control (N) lane, lacking template DNA, shows no detectable amplification, confirming specificity and absence of contamination.

(C–D) Representative Sanger sequencing chromatograms spanning the 5′ and 3’ junctions of the ALFA-tag knock-in at the Drd2 gene locus. Traces show high-quality, well-resolved base calls across the genomic sequence upstream of the insertion site, the ALFA-tag coding sequence, and the downstream genomic sequence. Individual base calls are aligned below the chromatograms, with vertical bars indicating exact matches to the reference sequence. No insertions, deletions, or sequence ambiguities were detected at the junctions. Phred quality metrics are shown for the displayed window (Q-scores) confirming high-confidence base calling.

(E) Representative RNAScope readout of Drd2 mRNA transcripts shows ALFA-tag expression is observed in neurons that are enriched in Drd2 mRNA puncta. Drd2 mRNA puncta were detected in n=34 of 35 cells that expressed the ALFA-tag.

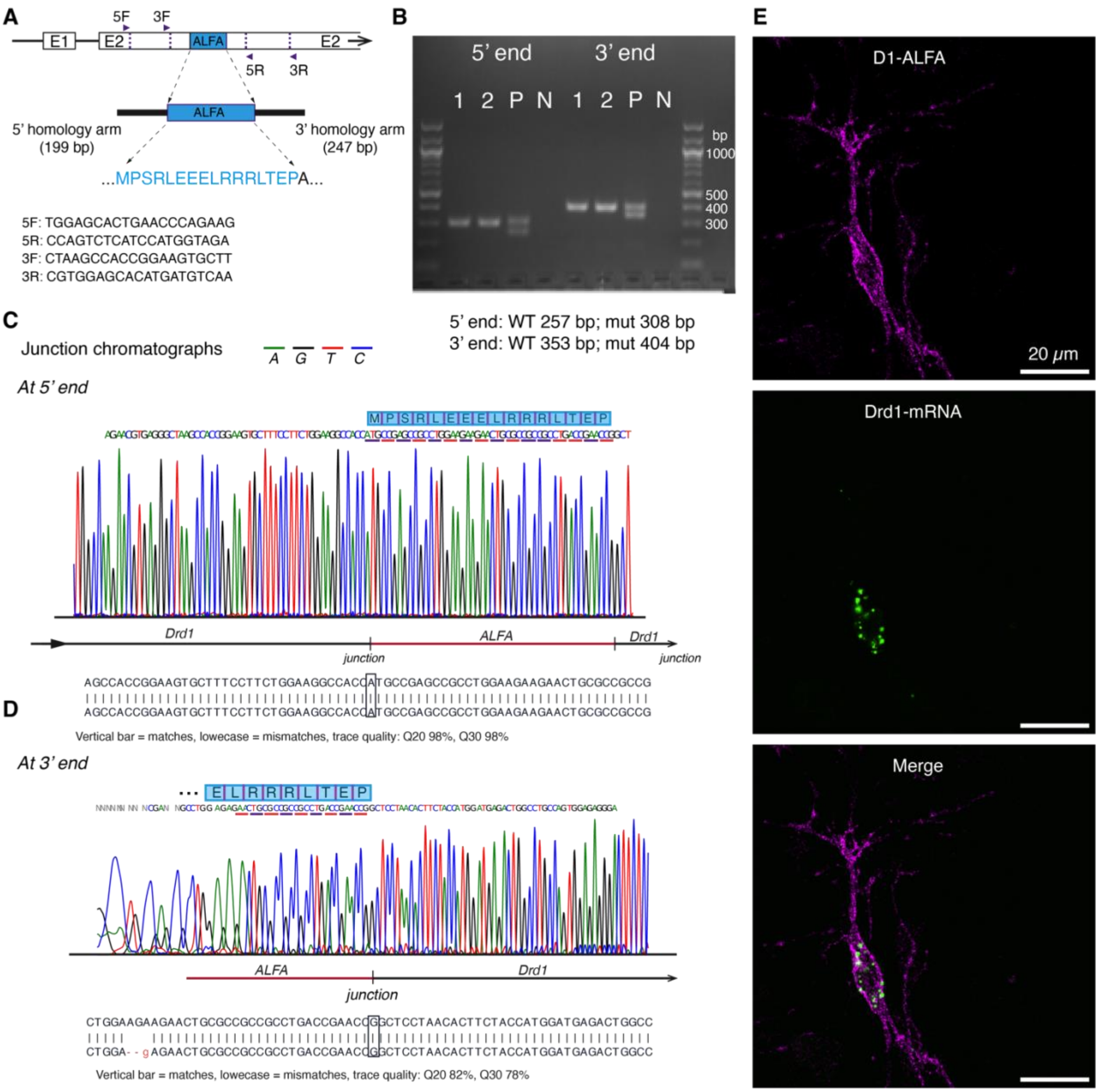

Figure S9. Design and validation of ALFA-tag knock in into the Drd1 gene locus.

(A) The design schematic of ALFA-tag insertion into the second exon (E2) of Drd1 gene, with 199 bp and 247 bp homology arms on the 5’ and 3’ ends respectively. 5’ and 3’ genotyping primers are shown.

(B) Gel electrophoresis of PCR products confirming correct integration at both the 5′ and 3′ junctions. Two biological replicates are shown. The positive control (P), containing a mixture of wild-type (WT) and knock-in alleles (mut), displays two closely migrating bands corresponding to the WT and mut products. The negative control (N) lane, lacking template DNA, shows no detectable amplification, confirming specificity and absence of contamination.

(C–D) Representative Sanger sequencing chromatograms spanning the 5′ and 3’ junctions of the ALFA-tag knock-in at the *Drd1* gene locus. Traces show high-quality, well-resolved base calls across the genomic sequence upstream of the insertion site, the ALFA-tag coding sequence, and the downstream genomic sequence. Individual base calls are aligned below the chromatograms, with vertical bars indicating exact matches to the reference sequence. No insertions, deletions, or sequence ambiguities were detected at the junctions. Phred quality metrics are shown for the displayed window (Q-scores) confirming high-confidence base calling. Low read quality upstream of the ALFA sequence in (D) is compensated by high quality reads on upstream of the ALFA-tag sequence in (C).

(E) Representative RNAScope readout of Drd1 mRNA transcripts shows ALFA-tag expression is observed in neurons that are enriched in Drd1 mRNA puncta. Drd1 mRNA puncta were detected in n = 5 of 5 cells that expressed the ALFA-tag.

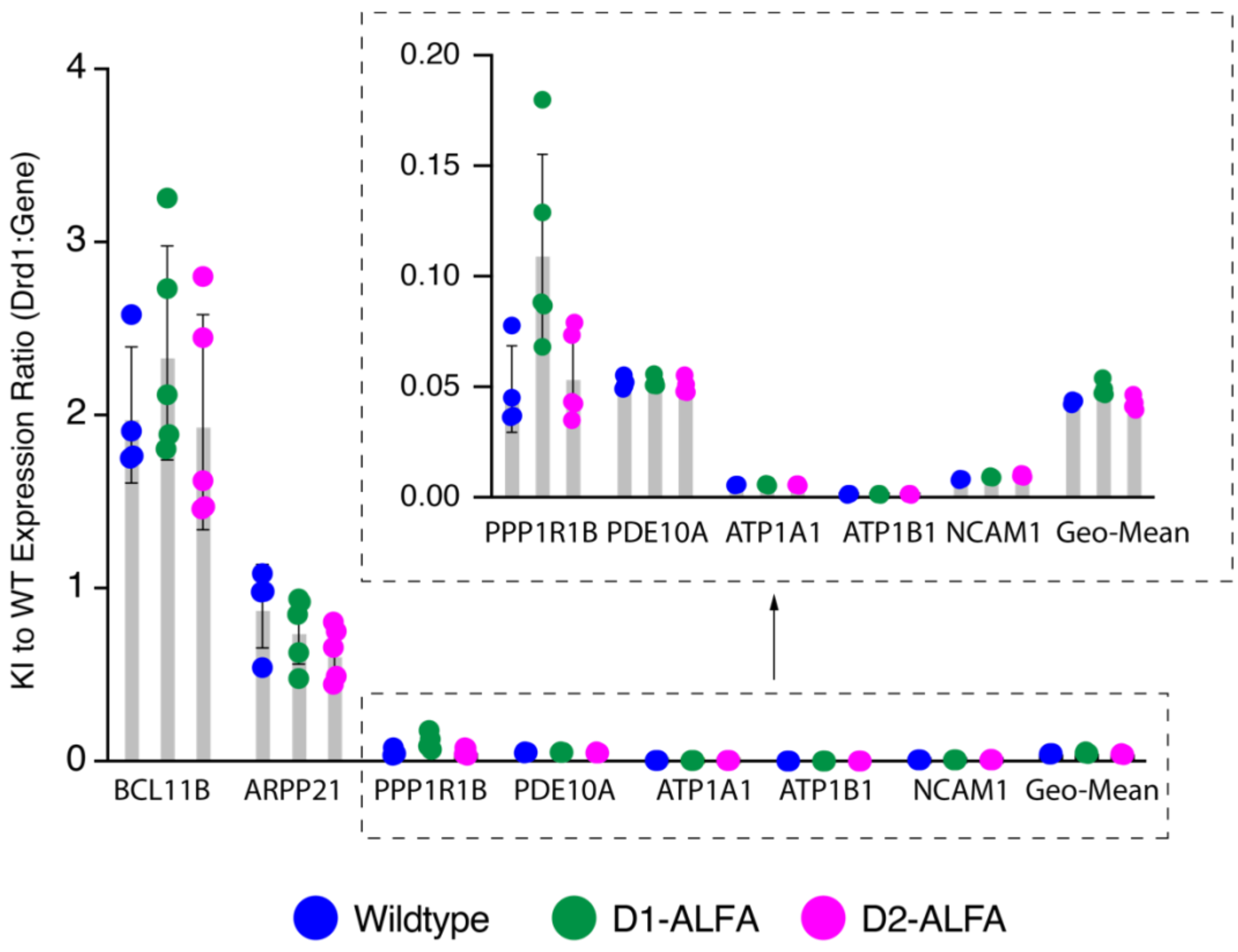

Figure S10. Quantitative proteomic validation of ALFADoR mice. Quantitative LC-MS/MS analysis of striatal lysates from wild-type (WT), ALFADoR1 (D1-ALFA), and ALFADoR2 (D2-ALFA) mice. D1 receptor abundance was comparable across all three genotypes following normalization to a panel of striatal control proteins, suggesting that ALFA-tag insertion does not measurably alter total D1 receptor expression. D2 peptides were not detected with sufficient confidence in whole-striatal lysates to permit similar quantitative analysis. Each point represents one technical replicate; mean ± SD bars are shown. Geo-Mean = geometric mean of the control genes. Statistics: pairwise comparisons (WT vs. D1-ALFA and WT vs. D2-ALFA) were performed for each protein using unpaired,

non-parametric Kolmogorov–Smirnov (KS) tests. All p-values were n.s., except: NCAM1 (p-value = 0.016 for both WT vs. D1 and WT vs. D2); and geo-mean (p-value = 0.016 for WT vs. D1).

Fixed sample imaging

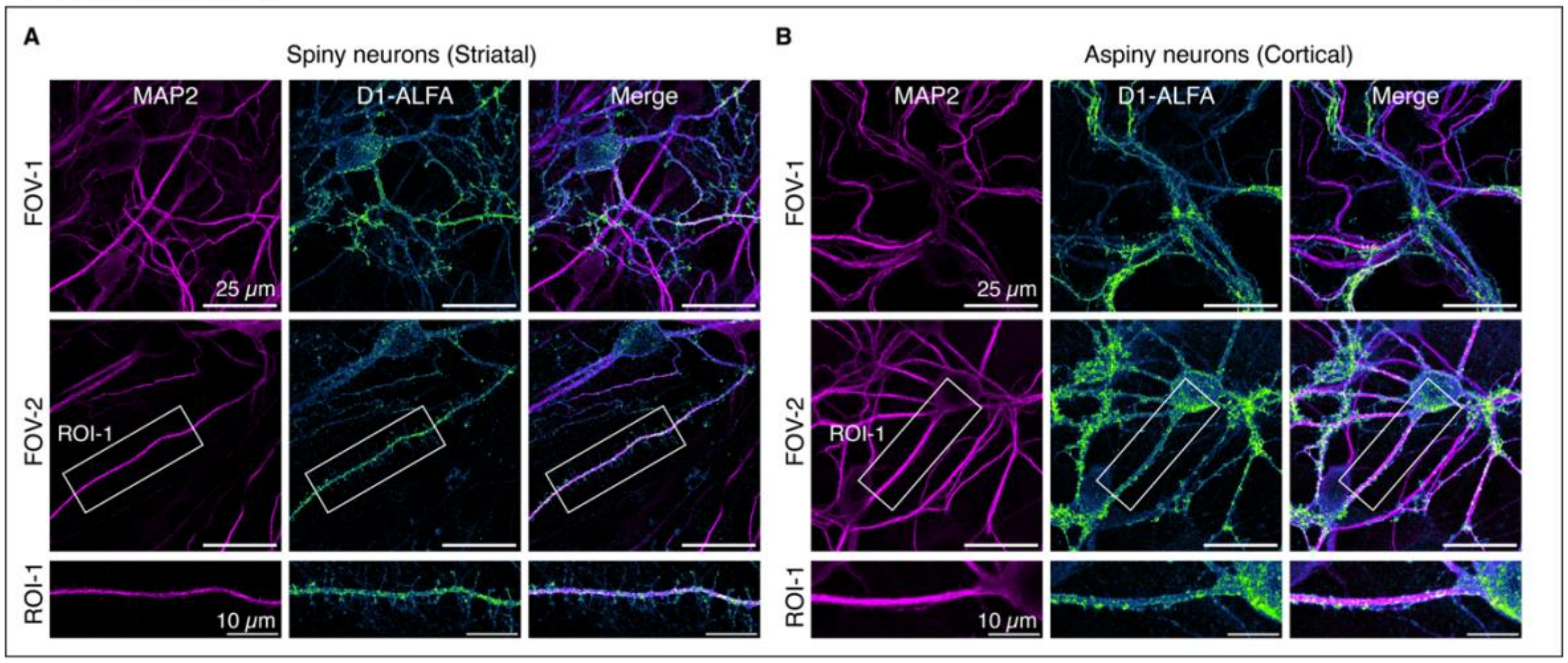

Live cell imaging

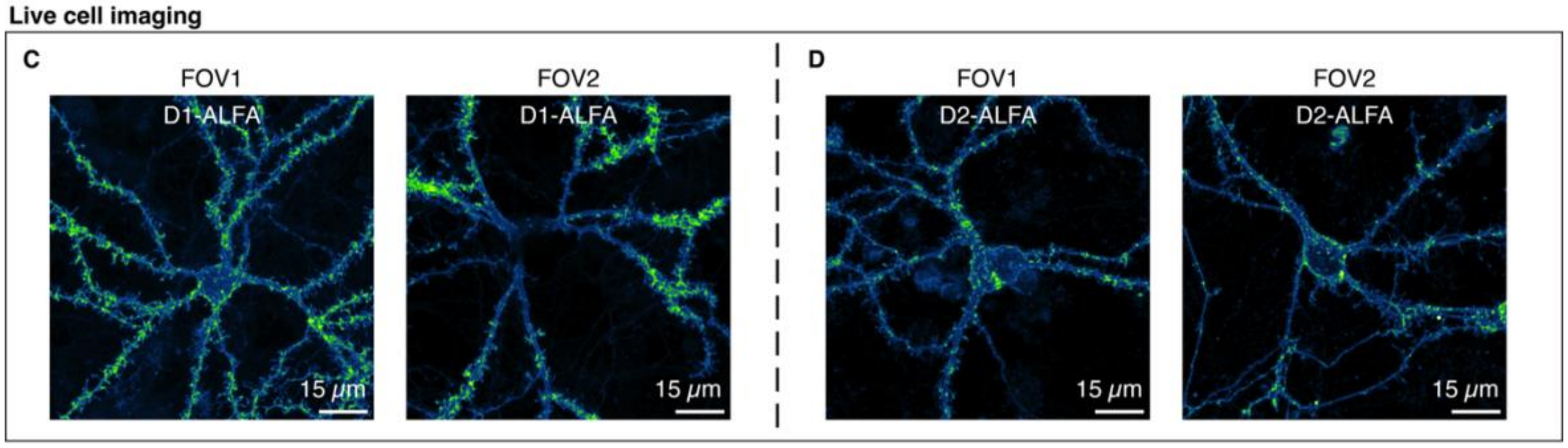

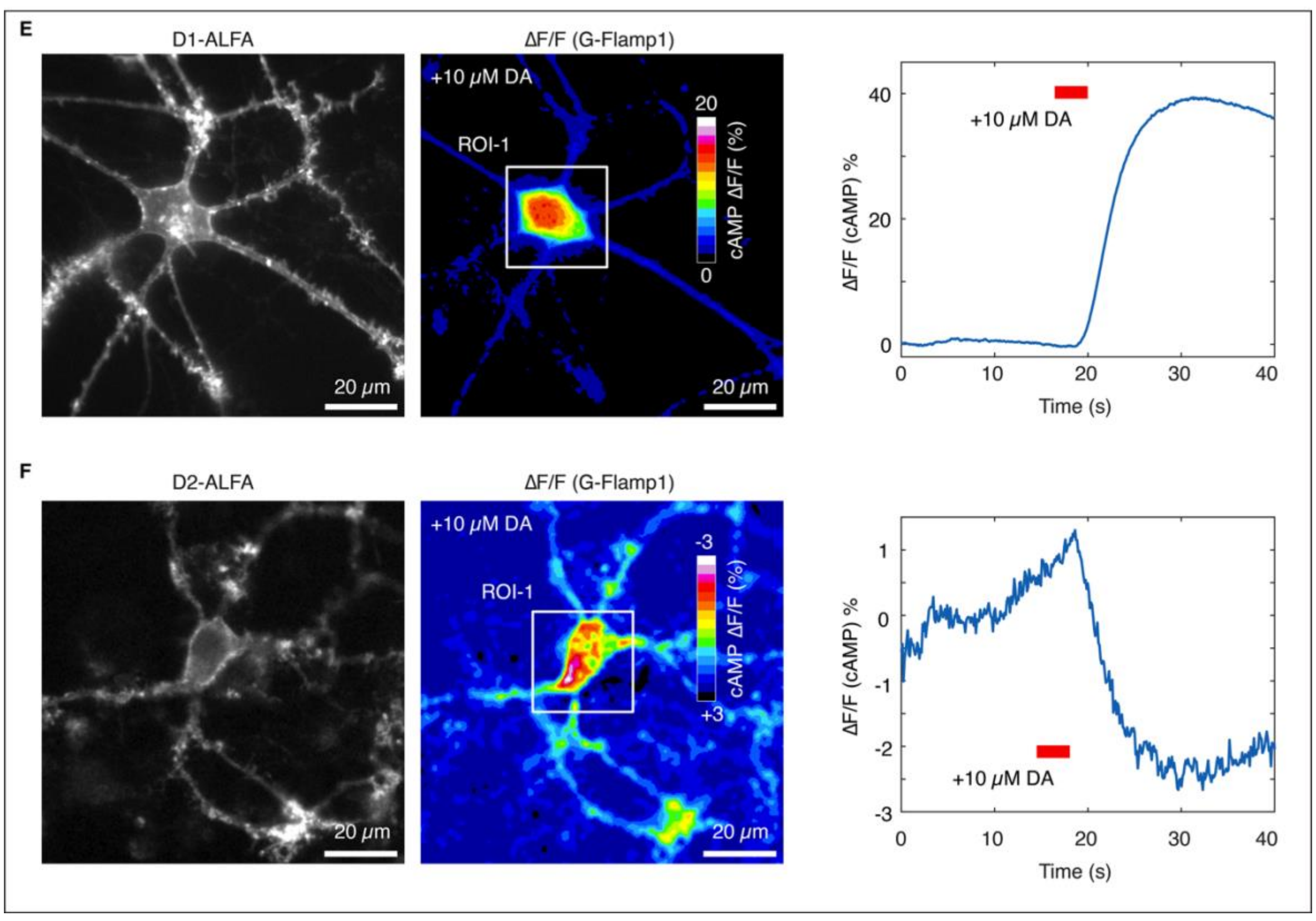

Figure S11. Visualizing D1-ALFA and D2-ALFA expression in fixed samples and live cells.

(A–B) D1-ALFA expression in spiny and aspiny fixed neurons, co-labeled with MAP2 to visualize soma and dendrites. D1-ALFA is present in soma, dendritic shafts and spines. Expression shows pronounced local non-homogeneity, appearing as a punctate distribution, as well as global inhomogeneity, with some dendritic segments exhibiting higher D1-ALFA levels than others.

(C–D) Maximum intensity projections of live-cell images of D1-ALFA and D2-ALFA expression using an anti-ALFA nanobody conjugated to Atto643 reveals punctate receptor localization, with differential enrichment in specific neuronal compartments (two representative examples are shown for D1-ALFA and D2-ALFA).

(E–F) Bath application of 10 µM dopamine (DA) to a G-Flamp1–expressing Drd1+ and Drd2+ neurons. Live-cell receptor imaging and cAMP activity imaging are shown. Note the non-uniform segregation of local cAMP modulations. Right side panels represent temporal traces of cAMP modulations.

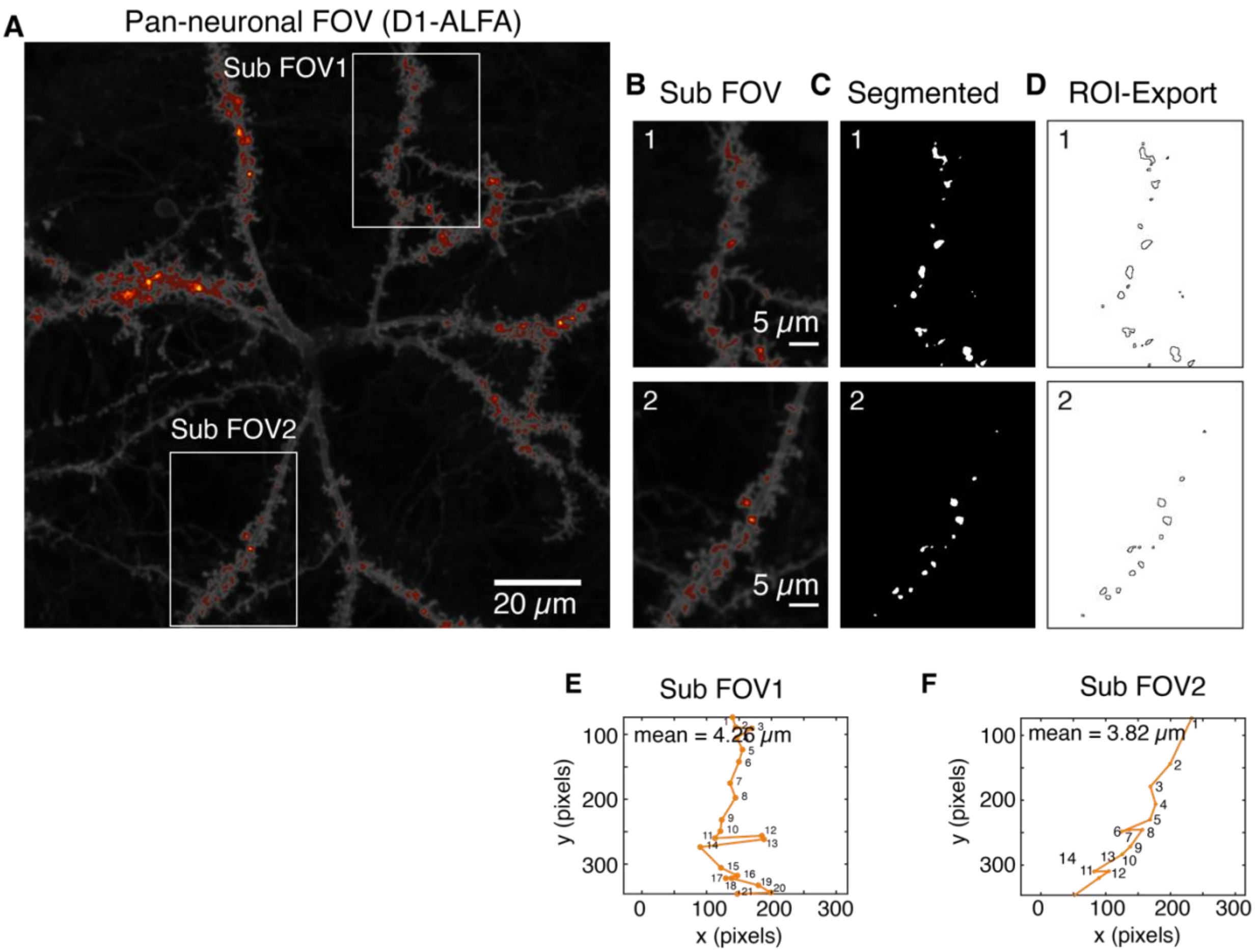

Figure S12. Interpuncta distance analysis of ALFA-tagged dopamine receptor puncta.

(A) Representative D1-ALFA signal across a whole neuron, illustrating heterogeneous receptor expression and the receptor-positive subregions selected for local interpuncta distance analysis.
(B–D) D1-ALFA puncta within selected subregions were segmented in Fiji, and the corresponding ROI sets were exported for analysis in MATLAB. ROI centroids were calculated from the segmented puncta.
(E–F) Euclidean distances between consecutive ROI centroids were calculated within each subregion, averaged across subregions, and then averaged across pan-neuronal fields of view to obtain one interpuncta distance value per neuron.

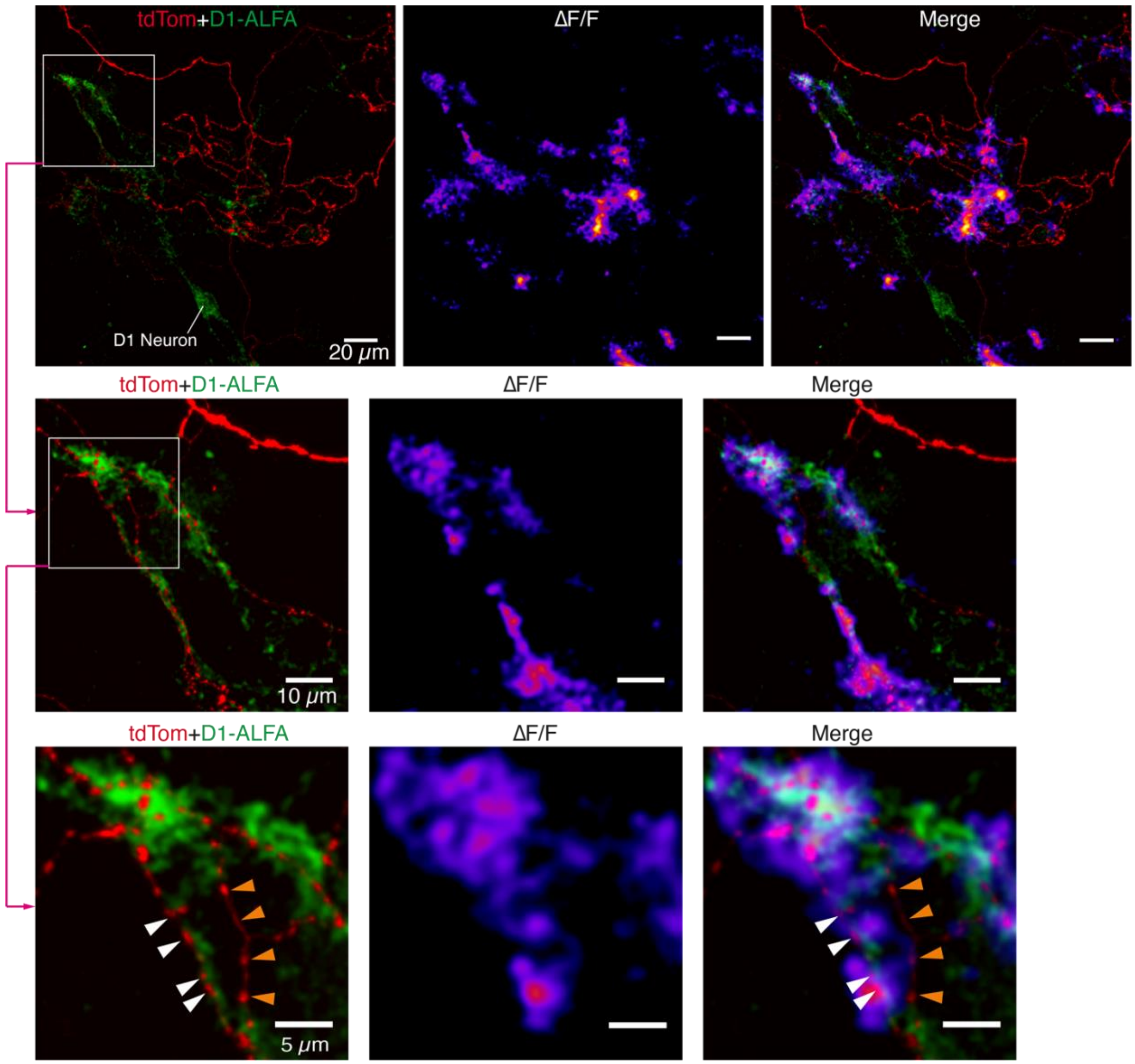

Figure S13. DopaFilm activity imaging in a dopaminergic axonal arbor arborizing in the vicinity of a D1-ALFA–tag expressing neuron.

DopaFilm signals are shown in a dopaminergic axonal arbor (tdTom, red) arborizing in the vicinity of a D1-ALFA–expressing neuron (green). Each row presents a progressively magnified view of a smaller region, as indicated by arrows and white boxes. Dopaminergic processes apposed to D1-ALFA–positive structures exhibit robust dopamine release, whereas non-apposed processes remain largely quiescent. Last row: white arrows indicate active varicosities, whereas orange arrows show quiescent ones.

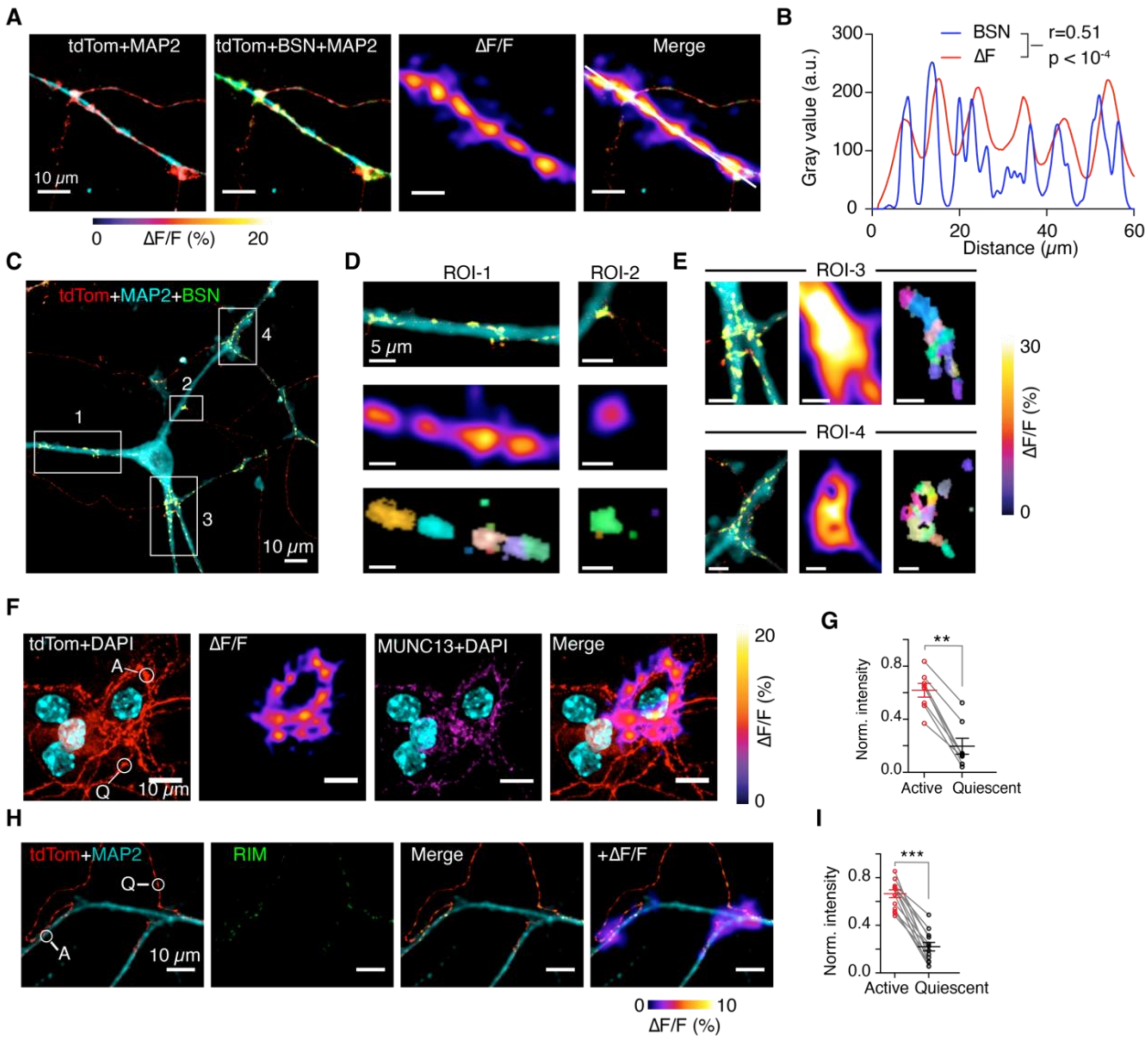

Figure S14. Synapse-like molecular enrichment at active dopaminergic varicosities.

(A) Dopaminergic axons apposed to dendrites (tdTomato, red; MAP2, cyan) exhibit robust dopamine hotspot formation (ΔF/F), whereas non-apposed axons remain largely quiescent. Bassoon (Bsn; green) is enriched at active varicosities, appearing as yellow where BSN overlaps with tdTomato. In contrast, quiescent varicosities show low BSN levels and appear predominantly red.

(B) Line profile of dopamine release (ΔF/F) and BSN expression demonstrates a strong correlation (Pearson correlation $r = 0.51$). The line used for the profile is indicated in the rightmost panel of (A).

(C) MAP2-stained cortical neuron (cyan) innervated by dopaminergic axons (tdTomato, red). Bsn puncta are visible as yellow overlap.

(D) Magnified views of ROI1 and ROI2 (white boxes in C). Bsn-enriched active varicosities are distributed along the dendritic process. Bottom panels show NNMF components, which group highly correlated pixels into distinct color-coded clusters, each corresponding to an individual dopamine hotspot detected by DopaFilm.
(E) Magnified views of ROI3 and ROI4 (white boxes in C). Multiple Bsn puncta are closely clustered. These “bunched” puncta give rise to clustered release sites and appear as merged signals (as ‘blobs’ instead of distinct

hotspots) in DopaFilm; however, NNMF decomposition resolves spatially distinct components corresponding to independent putative release sites (color codes, right panels).

(F–I) Enrichment of Munc13-1 and Rim at soma- or dendrite-apposed varicosities. Representative active (A) and quiescent (Q) varicosities are shown. Statistical significance was assessed using a Kolmogorov–Smirnov (KS) test (**$p < 10^{-2}$; ***$p < 10^{-3}$). Each point is an average over a biological replicate (typically a FOV containing hundreds of active and inactive varicosities). Mean and SEM bars are shown.

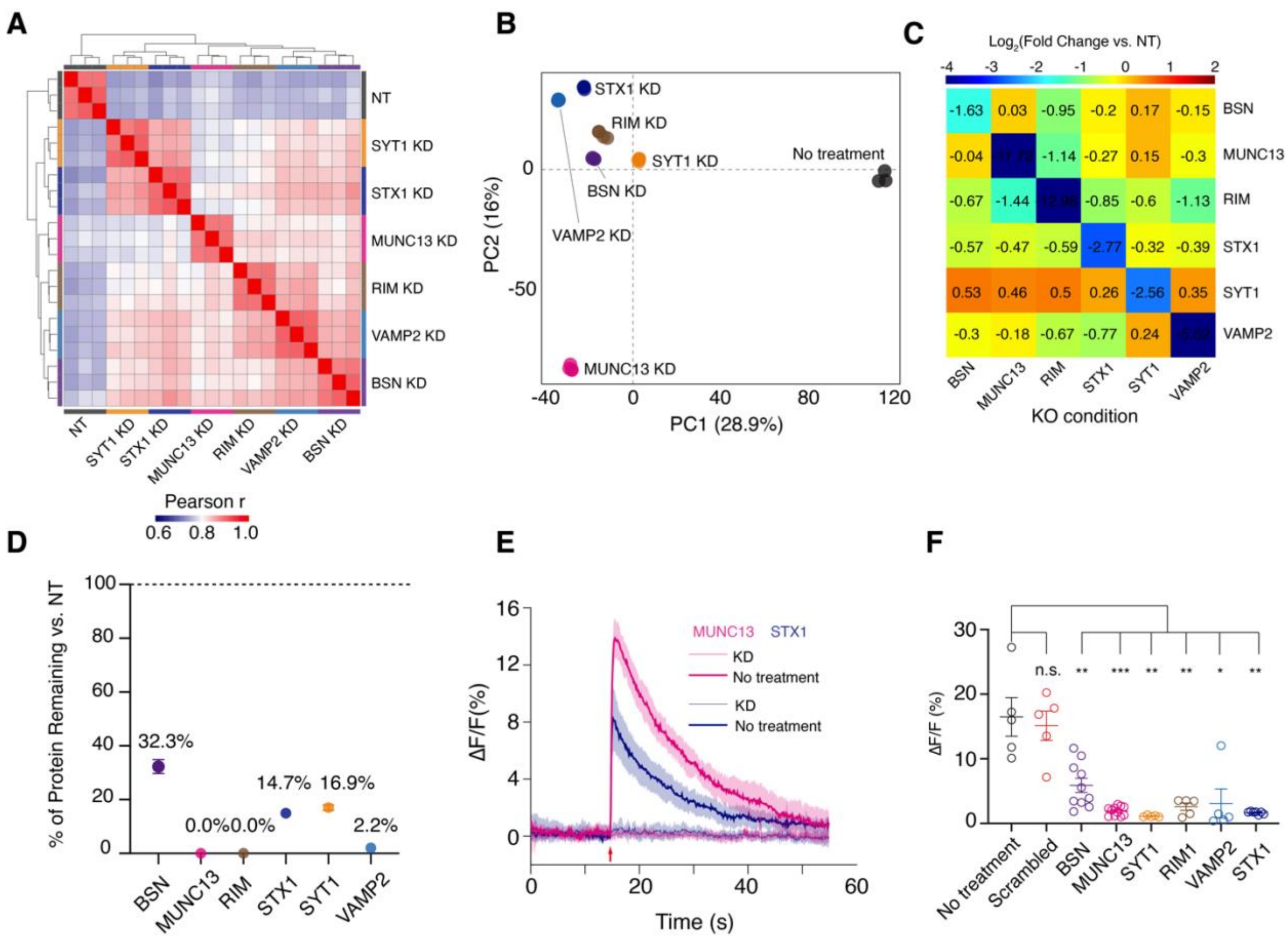

Figure S15. Assessment of knockdown (KD) efficacy using quantitative proteomics.

(A) Correlation heatmap (Pearson's *r*) across six KD conditions and no-treatment (NT) control demonstrates highly reproducible technical replicates (n = 3 per condition) and clear separation between experimental groups.

(B) Principal component analysis (PCA) shows tight clustering within each treatment group and strong spatial separation between the NT control and KD conditions, indicating robust proteomic differences following knockdown.

(C) Fold-change heatmap (KD conditions relative to NT) reveals efficient target protein depletion following treatment, while also highlighting cross-depletion relationships among different KD conditions.

(D) Log2 fold-change values were converted to percent protein remaining, illustrating near-complete depletion for Munc13-1 and Rim knockdowns and overall strong depletion efficiency across most KD conditions.

(E) Representative dopamine activity imaging traces from a KD condition and an NT control. Munc13-1 and Stx1 are shown as an example.

(F) Quantification of peak ΔF/F responses for NT controls, scrambled controls, and KD treatments. Each point represents averaged ΔF/F activity from a biological replicate. Statistical significance was assessed using a Kolmogorov–Smirnov (KS) test (n.s., not significant; p-values: * < 0.05; ** $< 10^{-2}$; *** $< 10^{-3}$). Mean ± SEM bars are shown.

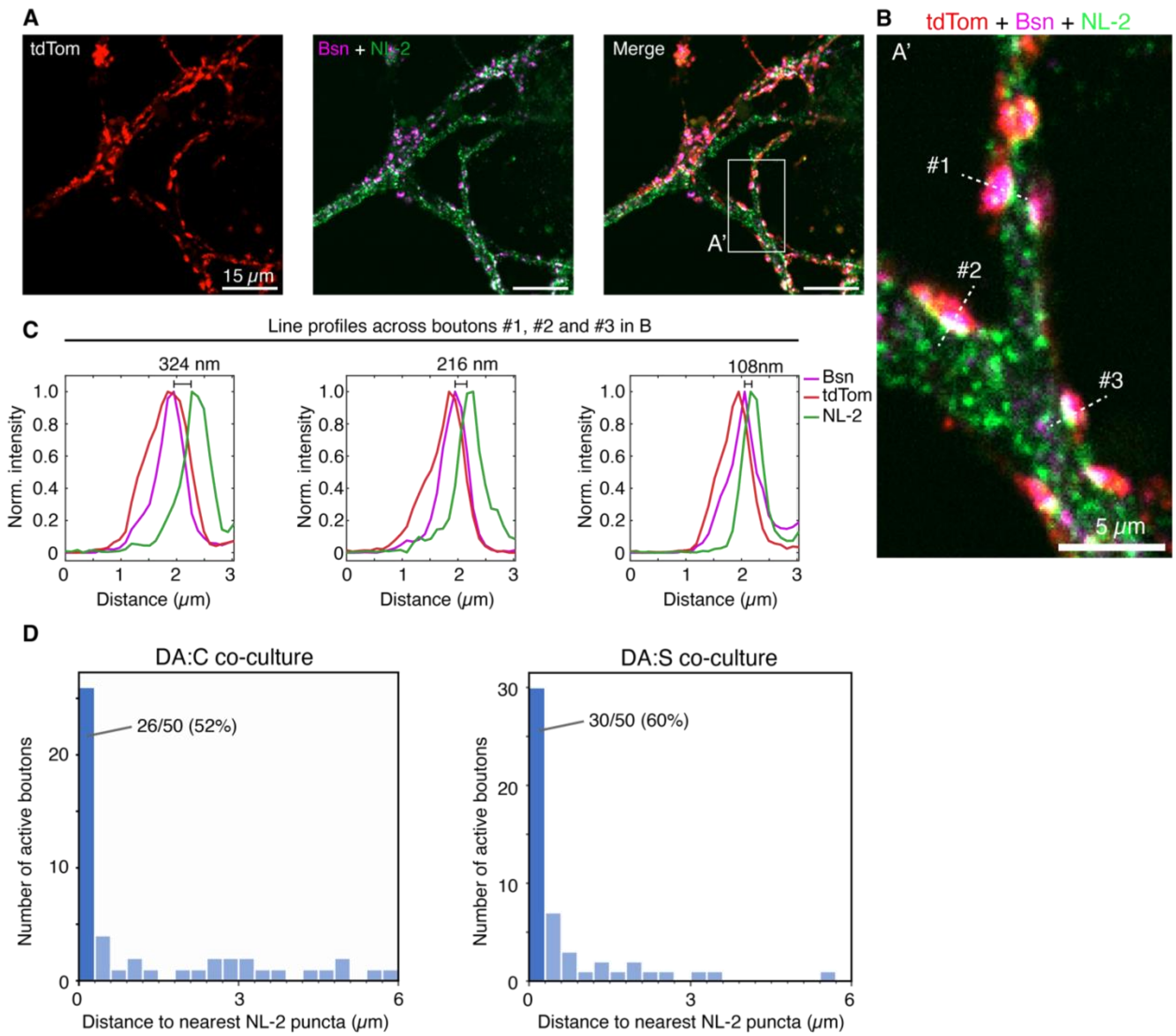

Figure S16. Bassoon-positive dopaminergic boutons are associated with Neuroligin-2 (NL-2)-positive structures.

(A–B) Representative DA:S co-culture showing dopaminergic axons (tdTom, red), Bassoon (Bsn, magenta), and Neuroligin-2 (NL-2, green). Merged image illustrates frequent apposition of Bsn-positive dopaminergic varicosities with NL-2 positive puncta.
(C) Representative line intensity profiles across three Bassoon-positive varicosities (depicted in B) demonstrating close spatial alignment of tdTom, Bsn, and NL-2 signals.

(D) Post hoc ICC shows the majority of active varicosities are apposed to NL-2 positive puncta in the target processes. Histogram of distances between active varicosities and the nearest NL2 punctum (measured from diffraction limited image analysis such as shown in (C).

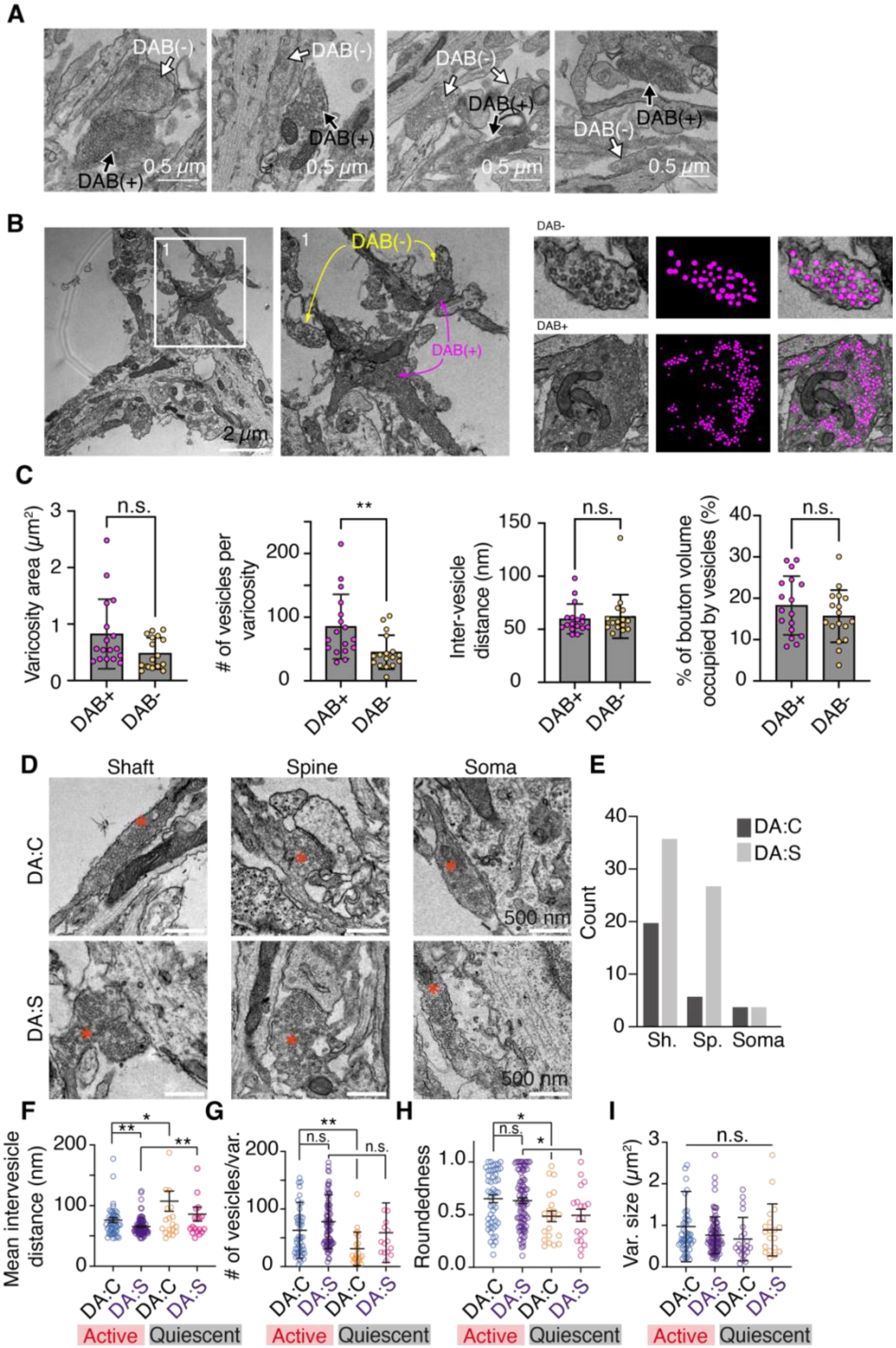

Figure S17. APEX2-mediated DAB deposition for ultrastructural analysis of dopaminergic (DAB+) and non-dopaminergic varicosities (DAB-).

(A) Representative transmission electron microscopy (TEM) thin sections showing DAB-positive (DAB+) and DAB-negative (DAB-) varicosities.

(B) A thin section containing DAB+ and DAB- varicosities. ROI1 (white box) is magnified to show DAB+ and DAB- varicosities within the same field of view. Right panels: DAB+ and DAB- varicosities, synaptic vesicle masks (magenta) and overlaid images are presented.

(C) Quantitative comparison of vesicle density and vesicle morphology between DAB+ and DAB- varicosities. Statistical significance was assessed using a Kolmogorov–Smirnov (KS) test (n.s., not significant; ** < 10^{-2}). Mean ± SD are shown.

(D) Representative ultrastructural images of DAB+ varicosities in DA:C and DA:S co-cultures, forming appositional contact with dendritic shafts (Sh.), spines (Sp.), and soma.

(E) Number of appositional contacts of each category visualized in survey of TEM thin sections.

(F–I) Comparison of active and quiescent varicosities (both DAB+). Each point represents average statistic over a varicosity. Mean ± SEM bars are shown in F, G, H and mean ± SD bars are shown in I. Roundedness in H refers to vesicle. Significance was evaluated with a nonparametric two-sample KS test; p-values: * < 0.05, ** < 10^{-2}, n.s. = not significant.

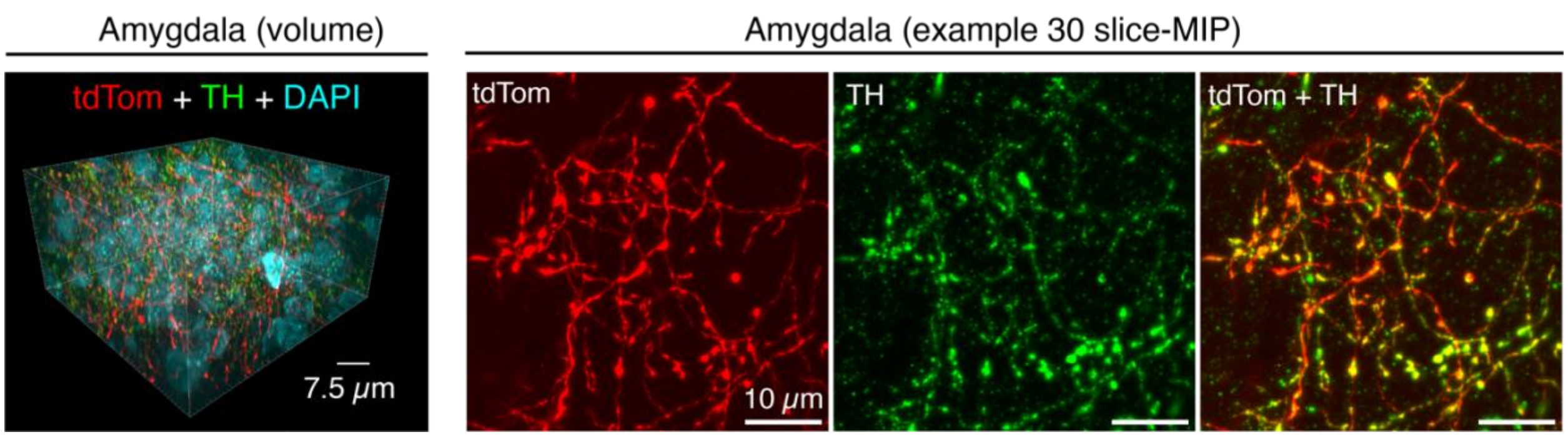

Figure S18. Validation of TH expression in dopaminergic axons in expanded amygdala tissue.

Expansion microscopy (ExF ≈ 4) was performed on amygdala tissue in which dopamine neurons expressed tdTomato (tdTom), followed by immunostaining for TH and DAPI. Representative 30-slice maximum intensity projections show colocalization between tdTomato and TH signals. Quantification showed that 65.9% of TH-positive varicosities were also tdTomato-positive.
